# Development and research trends of stay-green biology in legumes: a bibliometric and visual analysis over three decades

**DOI:** 10.3389/fgene.2026.1800699

**Published:** 2026-04-24

**Authors:** Shubh Pravat Singh Yadav, Upama Adhikari, James R. Myers, Lyle T. Wallace

**Affiliations:** 1 Department of Agricultural Sciences and Engineering, Tennessee State University, Nashville, TN, United States; 2 Department of Horticulture, Oregon State University, Corvallis, OR, United States

**Keywords:** bibliometric analysis, functional genomics, leaf senescence, molecular breeding, stay-green trait, stress resilience, visualization analysis

## Abstract

**Background:**

The stay-green trait, which manifests as a delayed leaf senescence in plants, is increasingly viewed as a valuable target for improving crop resilience, quality and yield stability. While most of the progress in this area has been made in cereals, research in legumes remains less consolidated, despite their importance for nutrition and sustainable agriculture.

**Methods:**

Bibliometric and structured literature reviews were combined to examine the evolution, thematic structure, and research frontiers of stay-green research in legumes over the past 3 decades. Using the Web of Science Core Collection and Dimensions database, 157 relevant articles published between 1993 and 2025 were identified following PRISMA guidelines and analyzed using VOSviewer and the Bibliometrix R framework to assess publication trends, collaboration networks, thematic evolution, and to classify reported stay-green phenotypes into functional and non-functional categories.

**Results:**

The results show a steady rise in publications with a growth rate of 8.6% per year, involving 883 authors across 96 journals, and a strong pattern of international collaboration. Most publications were original research articles, with only 11 review articles, indicating a lack of integrative work in this field. Foundational work by Thomas and colleagues remains highly influential, while recent studies increasingly emphasize molecular genetics and functional analyses in soybean, common bean, pea, and other grain legumes. The keyword analysis highlighted five main research hotspots: drought tolerance, molecular regulation of senescence, photosynthesis related mechanisms, trait mapping and genomics, and pathological stay-green syndromes. Research emphasis has shifted from descriptive physiology toward molecular breeding applications, with increasing focus on distinguishing functional from non-functional stay-green.

**Conclusion:**

This is the first comprehensive study to apply bibliometric approaches to analyze the trends and research frontiers of stay-green traits in legumes, offering valuable insights and reference points for advancing future research and breeding applications.

## 1 Introduction

Legumes, members of the Leguminosae or Fabaceae family, have long served as a foundation of human nutrition and sustainable agriculture due to their nutrient-dense seeds and unique ability to fix atmospheric nitrogen. The vast diversity of approximately 20,000 legume species is largely attributed to their adaptability, especially to low nutrient environments, largely due to their ability to biologically fix atmospheric nitrogen through symbiosis with *Rhizobiaceae* bacteria (Stoddard, 2017), and their ability to be easily integrated into crop rotation (Vaz Patto et al., 2015). Among them, the most extensively grown legumes include common bean (*Phaseolus vulgaris*), lentil (*Lens culinaris*), faba bean (*Vicia faba*), pea (*Pisum sativum*), cowpea (*Vigna unguiculata*), chickpea (*Cicer arietinum*), pigeon pea (*Cajanus cajan*) (Kumari et al., 2024), soybean (*Glycine max*) (Hamza et al., 2024), mungbean (*Vigna radiata*) (Diatta et al., 2024) and adzuki bean (*Vigna angularis* var. *angularis*) (Pandiyan et al., 2021). They are widely accessible, affordable, and considered safe for consumption, making them a mainstay of diets for millions of individuals worldwide (Martín-Cabrejas, 2019). Globally, legume production covers oilseed, dry grain, and vegetable forms, reflecting both their dietary and economic importance. Table 1 shows worldwide production statistics for major legume crops, classified by FAOSTAT commodity categories. Despite their importance, legume productivity can be impacted by various biotic and abiotic stresses, including heat, drought, salinity, and nutrient deficiencies (Dasgupta et al., 2025).

**TABLE 1:** Worldwide production of major legume crops classified as oilseed, dry grain, and vegetable types according to Food and Agriculture Organization of the United Nations (2021), with percentage change over the last decade.

Type	Crop	Species	Production MT	Change in production in last decade (%)
Oilseed	Soybeans	*Glycine max*	371,693,593	42
Oilseed	Groundnuts, excluding shelled	*Arachis hypogaea*	53,926,894	31
Vegetable	Peas, green	*Pisum sativum*	20,529,759	21
Vegetable	Broad and horse beans, green	*Vicia faba*	1,725,396	7
Vegetable	String beans	Predominantly *Phaseolus vulgaris* but may include *V. unguiculata* 'yardlong' types	1,310,002	−32
Vegetable	Other beans, green	Mainly *Vigna unguiculata* and *V. mungo* but may include other *Vigna* spp. and *Phaseolus vulgaris* and *P. lunatus* when used in green form	23,411,173	16
Dry	Chickpeas, dry	*Cicer arientinum*	15,871,846	35
Dry	Peas, dry	*Pisum sativum*	12,403,522	17
Dry	Cowpeas, dry	*Vigna unguiculata*	8,986,191	80
Dry	Broad beans and horse beans, dry	*Vicia faba*	5,964,384	30
Dry	Lentils, dry	*Lens culinaris*	5,610,104	28
Dry	Pigeon peas, dry	*Cajanus cajan*	5,477,111	31
Dry	Beans, dry	Predominantly *Phaseolus vulgaris* but may include various *Vigna* spp. other than *V. unguiculata*	27,715,024	15
Dry	Bambara beans, dry	*Vigna subterranea*	239,607	60
Dry	Vetches	*Lathyrus sativus* and *Vicia* spp. other than *Vicia faba*	626,255	−33
Dry	Other pulses*	*Dolichos* spp.*, Canavalia* spp.*, Psophocarpus tetragonolobus, Cyamopsis tetragonoloba Stizolobium* spp. and *Pachyrrhizus erosus*	4,687,855	34

Source: FAOSTAT, database (2021), *Not elsewhere classified. Note: In the FAOSTAT, database, legumes are categorized based on end use, where “dry” refers to harvested grain, “green” denotes shell-out forms and “string beans” refers to snap or fresh pod types.

In this context, the stay-green (SG) trait has attracted increasing attention in plant breeding. Stay-green mutations are classified into two types: functional and non-functional stay-green (Latif et al., 2020). Functional stay-green is characterized by prolonged photosynthesis and the accumulation of assimilates in harvestable tissues, making them a promising target for crop improvement. In contrast, non-functional stay-green maintains green foliage without photosynthesis, often due to either disrupted chlorophyll breakdown or impaired nitrogen translocation from leaves to seeds (Christopher et al., 2016). Non-functional stay-green has proven to be economically important for fruit and seed quality in some crops.

Although the stay-green trait is increasingly recognized as an important target for crop improvement, much of the progress so far has been made in cereals and model plants, while research on legumes remains relatively fragmented. Moreover, the small number of review articles highlights a clear gap in bringing together and contextualizing what is known in this topic. To bridge this gap, this study combines a bibliometric analysis with a literature review to provide a broad, yet detailed picture of stay-green research in legumes. We examine publication trends, citation patterns, leading journals, collaboration networks, and thematic hotspots to understand how the field has evolved. At the same time, we synthesize physiological, genetic, and molecular insights into stay-green, with a focus on its potential to sustain photosynthesis, improve yield stability, and strengthen stress resilience in legumes. In doing so, this review not only maps the progress made but also points to emerging directions, research gaps, and opportunities that can guide future legume breeding programs.

### 1.1 STAY-GREEN trait

Stay-green refers to plant mutants, cultivars or transgenic lines that maintain their green coloration for a longer duration compared to wild-type plants (Kusaba et al., 2013). It is a heritable trait, which is marked by delayed leaf senescence in plants (Thomas and Ougham, 2014), and/or by altering the chlorophyll degradation process (Myers et al., 2018).

Functional stay-green is associated with extended leaf greenness during seed filling which enables plants to sustain photosynthetic activity for an extended period, ensuring balance of demand and supply of nitrogen at critical stage of seed filling (Borrell et al., 2001). Stay-green plants maintain CO_2_ uptake with leaves that remain photosynthetically active (Farooq et al., 2017). It also ensures prolonged biomass production (Clifton-Brown et al., 2002) and may contribute to a better harvest index. The functional stay-green trait is therefore considered a promising target for improving crop resilience, resource-use efficiency, and productivity, especially under stressful environmental conditions.

Functional stay-green is divided into type A (late initiation of senescence) and type B (slow progression of senescence) (Latif et al., 2020). The physiological advantages of functional stay-green (SG) traits are very significant in addition to prolonged photosynthetic activity, enhanced drought resistance, and disease resistance (Thomas and Smart, 1993), more efficient nutrient utilization, and increased crop productivity (Farooq et al., 2017). In both cereals and legumes, these traits have exhibited potential to enhance yields, particularly under stress or sub-optimal growing conditions.

The non-functional stay-green trait is associated with green foliage persisting after senescence and the cessation of photosynthesis. Non-functional stay-green is divided into type C (cosmetic), type D (pseudo stay-green), and type E (hyper green) (Hörtensteiner, 2009; Latif et al., 2020; Thomas and Ougham, 2014). Among these, cosmetic stay-green (type C) is characterized by delayed chlorophyll degradation without sustained photosynthetic activity or yield advantage. Despite this, cosmetic stay-green has proven economically important in several grain and vegetable legumes due to its effects on seed, pod, or foliage appearance and quality (Myers et al., 2018).

### 1.2 Molecular mechanisms of stay-green traits

In studies in legumes, a substantial proportion of experimentally characterized stay-green phenotypes corresponds to non-functional forms with demonstrated stability and utility, whereas evidence for genetically stable and agronomically robust functional stay-green remains comparatively limited. While the genetic and physiological mechanisms underlying the stay-green trait varies notably across different species (Thomas and Howarth, 2000), the stay-green trait is considerably associated with the leaf chlorophyll content since its reduction leads to noticeable senescence symptoms. Because chloroplasts hold most of the essential nutrients, they play a central role in the carbon and nitrogen cycles of plants (Zhao et al., 2022). Therefore, stay-green traits may arise from continuous chlorophyll synthesis or delayed chlorophyll degradation where the latter has been the most explored mechanism (Thomas and Ougham, 2014).

While chlorophyll a and b are present in green photosynthetic tissues of nearly all green algae and land plants (Fernández-Marín et al., 2018), chlorophyll degradation is initiated with the conversion of chlorophyll b to chlorophyll a. It involves the reduction of the 7-formyl group of chlorophyll b to a hydroxymethyl group, carried out by chlorophyll b reductase (Kusaba et al., 2007), which is then converted to chlorophyll a, catalyzed by 7-hydroxymethyl chlorophyll a reductase (HCAR). Subsequently, degradation of chlorophyll a begins with the removal of the Mg2+ ion by a metal chelating agent, resulting in the formation of pheophytin. The enzyme pheophytinase (PPH) then removes the phytol tail, producing pheophorbide a, which is then converted into a chlorophyll catabolite through the actions of pheide an oxygenase and chlorophyll catabolite reductase. Ultimately, the resulting chlorophyll catabolite is moved into the vacuole (Balazadeh, 2014).

Initially, STAY-GREEN (SGRs) homologs were thought to act as a recruiter protein in chloroplast senescence, facilitating chlorophyll degradation by associating with five core chlorophyll catabolic enzymes (CCEs); NYC1, PAO, PPH, HCAR and RCCR within the light-harvesting complex II (LHCII). A breakthrough in 2016 revealed that SGR homologs functioned as Mg-dechelatases in *Arabidopsis* and *Chlamydomonas reinhardtii*, directly facilitating the removal of magnesium from chlorophyll a (Jiao et al., 2020). Mutations in SGR genes disrupt this step of chlorophyll degradation and typically result in a non-functional stay-green phenotype, in which chlorophyll persists in older tissues without sustained photosynthetic activity. Homologs of SGR have been discovered and experimentally confirmed in legumes such as soybean (Nakano et al., 2014; Shi et al., 2016), pea (Armstead et al., 2007), faba bean (Chen et al., 2023), chickpea (Sivasakthi et al., 2019), common bean (Davis et al., 2010), alfalfa (Zhou et al., 2011), where mutation in those genes had been found to confer non-functional stay-green phenotypes. The expression and activity of SGR genes are mediated by complex regulatory networks such as transcription factors, hormonal pathway and various environmental stimuli. A wide range of stay-green (SGR) mutants have been identified across different plant species, most of these mutants result from mutations in specific genes, including SGR1, ORE9 and ORE10 (Shi et al., 2016). In addition to natural and induced mutants, transgenic approaches to attain the stay-green trait include elevating endogenous cytokinin levels, suppressing abscisic acid (ABA) synthesis, promoting chlorophyll production, and inhibiting its degradation process (Munaiz et al., 2020).

### 1.3 Stay-green and abiotic and biotic stress tolerance

Plants with functional stay-green have been found to be associated with better tolerance to abiotic stress including drought stress (Thomas and Smart, 1993), salinity stress (De Luche et al., 2015) and heat-stress (Abdelrahman et al., 2017), resulting from protection of photosynthetic structures against reactive oxygen species, such as peroxide and superoxide (Tian et al., 2012). The mechanism for functional stay-green being associated with stress tolerance is through the maintenance of photosynthetic activity (Thomas and Ougham, 2014).

In plants, seed development relies mainly on nitrogen absorbed from the soil during the grain-filling period and nitrogen remobilized from the plant’s vegetative tissues (Ta and Weiland, 1992). Studies suggest that functional stay-green plant types obtain greater proportion of nitrogen through continuous uptake from soil (Borrell et al., 2001). Under abiotic stress conditions like drought or nitrogen deficiency, the remobilization of nitrogen (Ta and Weiland, 1992) and assimilated carbon (Farooq et al., 2017) from vegetative tissues becomes especially crucial for supporting grain development. Moreover, stay-green loci have been reported to influence root structure and enhance water uptake during grain filling in water-deficit field environments (Manschadi et al., 2006). This abiotic stress also impacts reproductive activity of plants. During drought or heat stress, premature floret abortion mainly occurs due to deficiency of starch caused by decreased photosynthetic activity (Ji et al., 2011). In addition to this, decreased photosynthesis induced by abiotic stress reduces carbohydrate reserves and causes rapid breakdown of starch in the ovaries, triggered by amylase activity as ovary growth begins. This can eventually result in seed abortion (Ruan, 2014). Thus, under stress conditions, steady supply of carbohydrates to the anthers and ovaries is crucial for meeting energy demands and maintaining the viability of pollen and ovules (Abdelrahman et al., 2017). This may be achieved through functional stay-green traits, which prolong photosynthetic activity (Dolferus et al., 2011; Jagadish et al., 2015). In this context, Ismail et al. (2000) reported that in cowpea (*V. unguiculata*), the delayed leaf senescence (DLS) trait, which is closely associated with the stay-green trait, played an important role in enhancing plant survival and enabling a second flush of pod production under prolonged and stressful growing conditions. Supporting this, Muchero et al. (2009) found that the expression of the stay-green trait in cowpea is strongly associated with increased grain and biomass yield under drought conditions. In plants, functional stay-green trait maintains active chloroplasts, leading to sustained photosynthesis, which in turn ensures the continuous production of metabolites required in vital processes like carbon fixation, fatty acid synthesis, and nitrogen assimilation into amino acids (Nunes-Nesi et al., 2010). This chloroplast based, light driven pathways can influence plant defense responses (Delprato et al., 2015). Stay-green plants are also found to show improved disease tolerance (Thomas and Smart, 1993).

In specific instances, the stay-green trait may confer biotic stress resistance. For functional stay-green, Wang et al. (2019) found that a loss of susceptibility mutation in *CsSCR* gene resulted in a stay-green phenotype that delayed chlorophyll degradation while maintaining leaf integrity and cellular homeostasis, thereby preventing chlorosis and conferring durable, broad spectrum disease resistance. This resistance was attributed to the suppression of reactive oxygen species over accumulation and phytotoxic chlorophyll catabolite buildup, distinguishing it from cosmetic chlorophyll retention. Another example of a loss-of-susceptibility mutation in stay-green (SGR) genes is non-functional stay-green in soybean lines carrying double mutation in *GmSGR1* and *GmSGR2* retained chlorophyll under exposure to *Fusarium virguliforme* phytotoxins but exhibited necrosis and reduced photosynthesis (Chang et al., 2019).

### 1.4 Advances in molecular breeding for stay-green trait

Breeding efforts have focused on identifying functional stay-green, particularly where delayed senescence is associated with improved biomass accumulation and yield stability. In cowpea, QTL mapping and association analyses identified major stay-green loci (Dro-1, Dro-3, Dro-7) that show positive pleiotropic effects on biomass and grain yield under drought, providing effective targets for marker-assisted selection (Muchero et al., 2013). Similarly, GWAS in common bean detected multiple genomic regions controlling functional stay-green under terminal drought, implicating genes related to photosynthesis, carbohydrate metabolism, antioxidant activity, and hormone signaling (Labastida et al., 2023). In soybean, transcriptomic, physiological, and mutant-based studies have further supported functional stay-green by linking delayed senescence with sustained photosynthetic efficiency and yield performance (Bawa et al., 2021; Wang et al., 2021a).

Alongside these efforts, non-functional stay-green has been characterized at the molecular level, particularly through mutations affecting chlorophyll degradation and plastid dismantling pathways. In soybean, early genetic and physiological studies demonstrated that recessive mutations at *d1* and *d2* loci inhibit chlorophyll and thylakoid membrane degradation during senescence, resulting in persistent greenness (Guiamét and Giannibelli, 1994; Guiamét et al., 1999). Moreover, subsequent molecular analyses revealed that *cytG*, encoding a chloroplast protein (*PsbM*), disrupts chlorophyll breakdown through frameshift or insertion mutations, leading to cosmetic stay-green phenotypes while simultaneously increasing susceptibility to photoinhibition and reducing stress adaptability (Canfield et al., 1995; Kohzuma et al., 2017). Near-isogenic soybean lines carrying *d1d2* mutations retained chlorophyll during senescence but exhibited reduced stomatal conductance and yield reduction under field conditions (Luquez and Guiamét, 2001; Luquez and Guiamét, 2002). Non-functional stay-green has also been widely exploited for seed-specific and quality-related traits. In soybean, mutations or regulatory variation in *SGR* homologs result in green seed or green cotyledon phenotypes caused by impaired embryo degreening during maturation (Delmas et al., 2013; Teixeira et al., 2016). The genetic analyses in snap bean identified the persistent color (*pc*) gene family as a cosmetic stay-green trait affecting pod and seed color, though associated with reduced seed vigor and higher pathogen susceptibility (Cirak and Myers, 2021). Similarly, QTL mapping in pea linked green cotyledon color to variation in pigment loss during seed maturation rather than prolonged photosynthetic activity (McCallum et al., 1997). Wolabu et al. (2020) also reported prolonged greenness in *Medicago sativa* (Alfalfa) by knocking out MsSGR gene using CRISPR/Cas 9 approach. The additional mutant analyses identified genes such as *MtNYC1*, involved in chlorophyll *b* degradation, and transcriptional regulators such as *ABI4*, which impair plastid dismantling during seed maturation and reduce seed longevity, providing molecular markers for non-functional stay-green phenotypes (Wang M. et al., 2025; Zinsmeister et al., 2023).

### 1.5 Applications for crop improvement

Identifying and understanding genes and pathways involved in senescence can aid in crop improvement by modifying crops to better use plant tissues for energy (Munaiz et al., 2020). In legume forage crops, delayed senescence is particularly relevant because market value is closely linked to leaf color and visual quality. Bright green foliage is a key quality attribute of premium alfalfa hay, and cosmetic stay-green traits that delay chlorophyll degradation can therefore enhance forage appearance and chlorophyll retention, potentially increasing antioxidant capacity (Zhou et al., 2011). A Tnt1 (tobacco transposable element) tagged mutant of *Medicago truncatula* (NF2089), caused by disruption of the MtSGR gene, exhibited delayed chlorophyll degradation and retained green tissues in leaves, anthers, carpels, pods, and seeds (Zhou et al., 2011). This phenotype represents a cosmetic stay-green, primarily affecting visual appearance rather than extending photosynthetically active growth. Similarly, RNA interference-mediated silencing of MsSGR in alfalfa produced stay-green transgenic lines with enhanced chlorophyll retention during senescence, improved visual quality, and increased crude protein content. Likewise, CRISPR/Cas mediated genome editing has been successfully applied to modify several agronomically important traits in legumes, including *Glycine max* (Jacobs et al., 2015; Zhang et al., 2020), *V. unguiculata* (Ji et al., 2019), *P. sativum* (Li G. et al., 2023), and *C. arietinum* (Badhan et al., 2021). However, improving functional stay-green through genome editing remains challenging, as this trait is typically controlled by multiple loci with small effects. In contrast, single-gene mutations causing non-functional stay-green are well documented in legumes, including mutations in GmSGR1/GmSGR2 in soybean (Nakano et al., 2014; Fang et al., 2014), the cytG mutation in soybean (Guiamét et al., 2002; Kohzuma et al., 2017), and SGR homologs in pea and faba bean that confer green cotyledons (Bell et al., 2015; Chen et al., 2023). These examples indicate that genome editing is currently more suitable for modifying visual or quality related stay-green traits than for enhancing functional stay-green in legumes. Recent advances in genomic tools have improved the manipulation of SGR and related single-gene targets, which are primarily associated with non-functional stay-green traits affecting chlorophyll retention and visual quality. Accordingly, breeding for functional stay-green legumes is likely to require quantitative approaches, such as QTL based selection and genomic prediction, rather than single-gene marker-assisted selection or genome editing.

### 1.6 Limitations and trade-offs of stay-green trait

Despite its potential benefits, the stay-green trait may have some undesirable effects on some crops, primarily grain and vegetable legumes (Myers et al., 2018). Because delayed senescence does not always reflect sustained photosynthetic activity, stay-green results from impaired chlorophyll degradation such as cosmetic or non-functional stay-green type do not confer yield or other agronomic advantages. In snap bean (*P. vulgaris*), one seed coat color phenotype used in cultivation is the persistent color (PC) type (Myers et al., 2018), a member of the stay-green gene family (Davis et al., 2010), that keeps seeds, pods and foliage green (Myers et al., 2018). However, a drawback of the persistent color trait is that cultivar expressing it show lower germination and field emergence than both white and colored-seed type (Al-Jadi et al., 2016). The reduction in fitness is associated with a thinner, more fragile testa as well as bleached cotyledons upon germination (Cirak and Myers, 2021). In grain soybeans, green seed color due to stay-green trait is undesirable as it lowers oil quality and seed viability. Due to the persistent chlorophyll and its derivatives, reactive oxygen species are generated causing rancidity and off flavors (Smolikova et al., 2017). On the other hand, green cotyledons are a desirable trait in vegetable soybeans. Type C stay-green may also limit the recycling of nitrogen, metal ions, and other nutrients necessary for growth, potentially reducing yield in crops with high sink demand compared to species with weak sink activity (Thomas and Ougham, 2014).

Functional stay-green can also involve trade-offs, particularly when delayed senescence interferes with timely maturity, assimilate remobilization, or crop dry-down. In chickpea, study performed on combined heat and drought stress found that genotype with longer reproductive duration and stay-green trait did not increase productivity. Delayed maturity was negatively correlated with yield under combined stress, therefore extended stay-green can be agronomically disadvantageous for chickpea in those specific environmental conditions (Gurumurthy et al., 2024). Studies have reported that the expression of the stay-green trait during late reproductive stages can be negatively associated with grain yield in cereals as well. In rice, evaluation of a double-haploid population derived from an indica × stay-green japonica cross revealed that delayed senescence was accompanied by yield reductions, likely due to inefficient assimilate remobilization during grain filling (Jiang et al., 2004). Likewise, Naruoka et al. (2012), also observed an inverse relationship between stay-green expression and grain yield in wheat grown under wet and cool conditions. Prolonged stay-green can further delay biomass dry-down at maturity, which interferes with mechanical harvesting and can increase the risk of field losses due to lodging or rotting (Yang et al., 2010). This is particularly relevant for large-scale production of soybean, dry beans, peas, lentils, faba beans, and chickpea, where timely dry-down is essential for efficient harvest (Haddad et al., 1988). This emphasizes the need for careful evaluation of stay-green effects in leguminous crops where distinct source-sink dynamics and nitrogen remobilization patterns may differentially influence yield and quality.

## 2 Materials and methods

### 2.1 Literature search procedures and selection of studies

The bibliometric analysis was conducted using the two databases, Web of Science (WoS) core collection and Dimensions due to their reliability and extensive coverage of peer-reviewed scientific literature. The data collection was performed using a combination of curated search strings and a search strategy targeting articles specifically related to stay-green in legumes. The search formula was divided into four focus areas: “General searches in legumes”, “Molecular breeding”, “Genomic tools”, and “Trait-targeted queries”, producing four respective search string constructs, including both scientific and common names of major legume species (Supplementary Material 1, Supplementary Table S1). This search strategy was designed to allow for a focused and efficient collection of information (Martínez-Saldarriaga et al., 2025).

**FIGURE 1:**
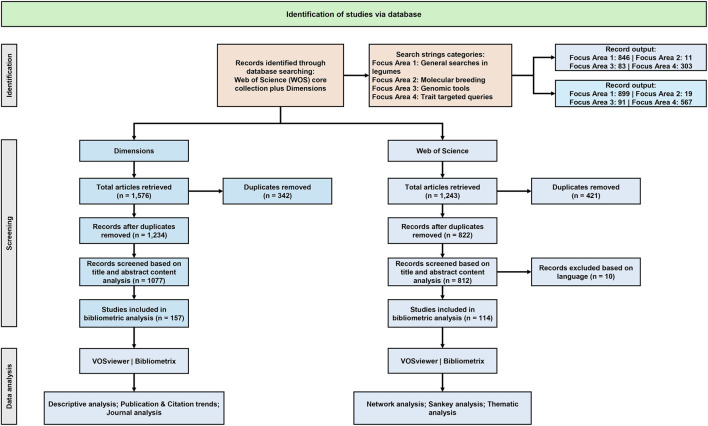
PRISMA flow diagram for study selection on stay-green research in legumes.

The Boolean search strings (using “AND” and “OR”) incorporated synonyms and variant gene names such as *“STAY-GREEN gene”, “stay-green”, “stay-green”, “SGR”, “SGR gene”, “SGR1”, “STG gene”, “SGR homolog”, “functional stay-green”, “non-functional stay-green”, “cosmetic stay-green”, “persistent green”, “persistent color”, “green cotyledon”, “green seed”, “seed coat color”* and their associations with relevant crops and research focus area. The search fields targeted the Title, Abstract, and Author Keywords, in the WoS core collection (Editions: All), while in the Dimensions database, the search was restricted to Title and Abstract field only, ensuring broad but relevant coverage across both databases. The search strategy also included defined exclusion criteria, such as limiting results by publication type, including only original and review articles. Wildcard symbols were used in the search terms. From the dimensions database, a total of 1,576 articles were retrieved. After removing 343 duplicated, 1,234 unique records remained. These were further screened based on title and abstract content (n = 1,077), resulting in 157 records. In case of WoS, a total of 1,243 articles were retrieved, of which 822 were unique after removing 421 duplicates. Further, language filtering was applied, retaining only English language articles (n = 812), filtering 10 articles. Lastly, for retrieving the most relevant articles, final screening through abstract content analysis was performed resulting in 114 articles. Further, the final set of studies identified from the WoS dataset was also represented within the Dimensions database. The entire screening and selection process followed the PRISMA guidelines for transparent and reproducible reporting of literature selection (Figure 1) (Page et al., 2021).

### 2.2 Data extraction and bibliometric tools

All the available bibliographic information was exported in plain text and CSV formats from both WoS and Dimensions platform (Supplementary Material 2). In this study, two widely used tools including VOSviewer (Ver. 1.6.20, Centre for Science and Technology Studies, Leiden University, Netherlands) (Van Eck and Waltman, 2010) and Rstudio (Ver. 2025.05.1, R Studio Inc., Boston, MA, USA) with the Biblioshiny interface from the “bibliometrix” R package (Aria and Cuccurullo, 2017) were used to perform the bibliometric analysis. Specifically, the dimensions dataset was used for descriptive analysis to capture broader publication coverage, whereas WoS dataset was used primarily for advanced bibliometric mapping due to its more standardized and structured metadata, thus producing accurate network analysis. The detailed network metrics and visualizations obtained from VOSviewer and bibliometrix related analysis are provided in the Supplementary Material for transparency and reproducibility (Supplementary Material 1, Supplementary Tables S1–S14; Supplementary Material 3; Supplementary Figures S1–S25).

### 2.3 Data analysis and visualization

The bibliometric analysis included trend analysis, country and institutional productivity analysis, journal analysis, keyword co-occurrence analysis, co-authorship network analysis and thematic mapping. The trend analysis was performed to evaluate the temporal distribution of scientific output on the stay-green research in legumes (Abdelwahab et al., 2024). This analysis provides insight into how scholarly attention toward the topic has evolved and helped to identify the most active periods, countries, and authors in the research. Further, the geographical distribution and institutional affiliation of authors were analyzed to determine the global research landscape. Keyword co-occurrence analysis was performed in VOSviewer to explore the conceptual structure of the field by mapping frequent and co-occurring keywords (Ardie et al., 2025; Krismawati et al., 2024). Co-authorship network analysis was performed to evaluate scientific collaboration at three levels, authors, organizations and countries (Ardie et al., 2025; Krismawati et al., 2024). The unit of analysis in each case was the co-authorship relation among entities, and full counting method was applied so that each co-authored publication counted equally for all contributing authors/institutions/countries. Likewise, the thematic analysis and diagram were performed using Biblioshiny to map the keyword clusters into thematic categories based on their development and relevance (Çelik, 2024). Further, a three-field analysis (Sankey diagram) was conducted to visualize the relationships between keywords, journals and countries (Zheng, 2024).

### 2.4 Classification of stay-green phenotypes in legumes

Following bibliometric screening, a content-based biological classification was performed to categorize the stay-green phenotypes reported in the final dataset. A total of 157 peer reviewed articles were subjected to this classification step. Each article was evaluated manually using its title and abstract, without relying on keyword based automation, to avoid misclassification caused by superficial mentions of the term *“stay-green”* (Supplementary Material 4). The articles were classified into three categories based on established physiological definitions of stay-green phenotypes.

1. Functional stay-green (F): Studies were classified as functional stay-green when delayed senescence was associated with maintenance of photosynthetic capacity and sustained carbon assimilation, and in some cases improved nitrogen remobilization, abiotic stress tolerance or positive effects on biomass accumulation or yield (Thomas and Howarth, 2000; Hörtensteiner, 2009).
2. Non-functional stay-green (NF): Studies were classified as non-functional (cosmetic) stay-green when the retention of green coloration resulted primarily from disruption of chlorophyll degradation pathways, without evidence of prolonged photosynthetic activity, stress resilience, or yield advantage (Hörtensteiner, 2009; Thomas and Howarth, 2000).
3. Cannot be classified: The articles were assigned to this category when the information provided in the title and abstract was insufficient or inappropriate to determine whether the stay-green phenotype was functional or non-functional. The articles involved pathological stay-green conditions (either pest infestation or viral infection), descriptive or agronomic scoring without physiological evidence, molecular or genetic studies lacking plant senescence evaluation, color trait focused studies unrelated to senescence or reviews and methodological papers where stay-green was mentioned only as background.

## 3 Results and discussion

### 3.1 General information of stay-green research in legumes in WoS

**FIGURE 2:**
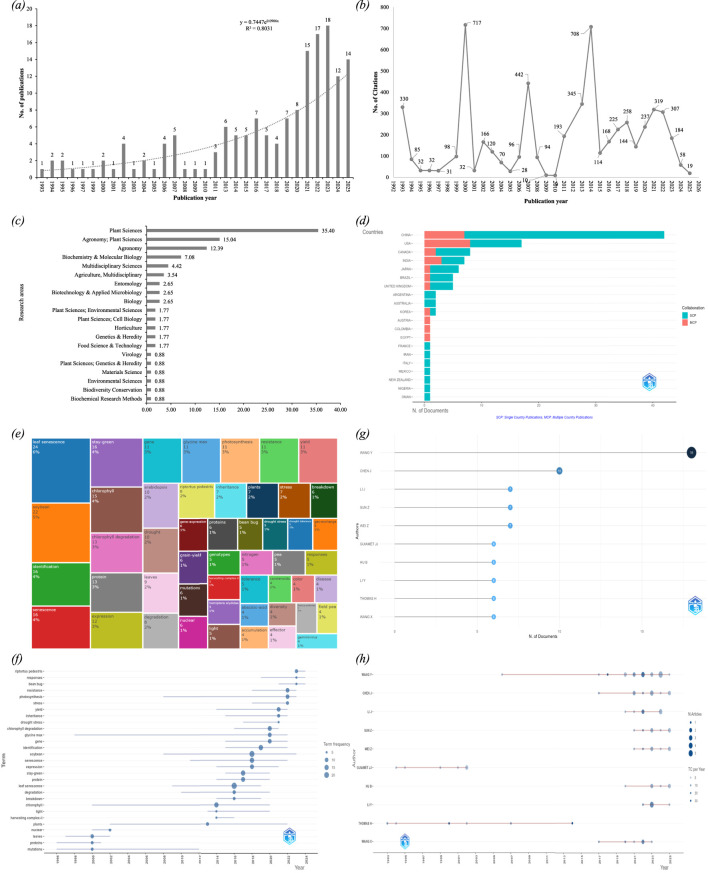
Bibliometric analysis of stay-green research in legumes including global trends, research areas, and key contributors. **(a)** Annual scientific production, shows the number of publications per year from 1993 to 2025, where vertical bars indicate yearly output and a dashed exponential trendline demonstrates a steady increase over time; **(b)** Citation counts by publication year, displays a line graph in which the x-axis represents the year of publication, y-axis shows the number of citations received by works published in that year and peaks highlight years of influential studies; **(c)** Research area distribution, research fields are shown on the y-axis, with horizontal bars reflecting their frequency in the dataset; **(d)** Country wise distribution of publications showing domestic and international collaboration patterns, where blue bars (SCP) represent single country publications, while the orange bars (MCP) indicate multi country publications; **(e)** Treemap visualization of the most frequent keywords; **(f)** Trend topics over time, maps the evolution of keyword term usage across years, horizontal lines show the lifespan of a term, while dot size indicates its peak frequency in the corresponding year; **(g)** Most relevant authors based on publication numbers, ranks top contributors by document count using dots aligned along the x-axis, larger and darker circles indicate higher output; **(h)** Authors’ production over time, charts the active publication years of key authors as horizontal lines, with size of the circle corresponding to the number of articles published by that author in that specific year, while the shade of blue reflects the total number of citations those publications received.

The bibliometric analysis comprised of 157 documents published between a time span of 1993–2025, across 96 journals, with an annual growth rate of 8.6%. Table 2 presents the descriptive statistics of the bibliometric analysis of the selected studies. The average document age was 9.23 years, and the field exhibited strong academic influence, with an average of 36.12 citations per document and a total of 5671 citations. Further, a total of 883 authors contributed to the dataset, with no single-authored publications, highlighting a strong collaborative nature. The average number of co-authors per paper was 7.5, and 22.93% of the publications involved international collaboration, reflecting the global interest in stay-green research in legumes. In terms of document types, original articles dominated (93%), with only eleven review articles identified. This suggests the field is largely driven by experimental studies. The descriptive metrics indicate a growing and collaborative field with high impact and diverse thematic focus, but also a notable gap in integrative review efforts. The content analysis revealed a rich conceptual structure, with 495 Keywords Plus and 393 Author Keywords, showing the multidisciplinary scope of the field, including plant physiology, leaf senescence, stress tolerance, photosynthesis, and molecular breeding. The dominance of these themes is reflected in keyword frequency rankings (Figure 2e; Supplementary Table S2; Supplementary Figure S1) and visually summarized in the keyword word cloud (Supplementary Figure S2), indicating the multidisciplinary nature of stay-green research in legumes. In addition, the classification of the literature based on stay-green phenotype revealed that 22 (14.01%) studies were functional, 40 (25.47%) were non-functional, while 95 (60.50%) could not be confidently classified due to insufficient functional evidence. Relatively, most studies are based on physiological observations and molecular associations and very few studies include functional validation through genetic or field based experiments. Thus, this imbalance limits the clear classification of stay-green types, contributing to a large proportion of studies remaining unclassified due to insufficient functional evidence. This indicates a gap between molecular identification and physiological confirmation.

**TABLE 2:** Descriptive statistics of the bibliometric analysis of selected studies of stay-green in legumes.

Description	Results
Main information about the dataset	
Timespan	1993:2025
Journals/Sources	96
Documents	157
Annual Growth Rate %	8.6
Document Average Age (years)	9.23
Average citations per doc	36.12
References	5109
Total citations	5671
Document contents	
Keywords Plus (ID)	495
Author’s Keywords (DE)	393
Authors details	
Authors	883
Authors of single-authored docs	0
Authors collaboration	
Single-authored docs	0
Co-Authors per Doc	7.5
International co-authorships %	22.93
Document types	
Article	146
Review	11
Article classification	
Functional	22 (14.01%)
Non-functional	40 (25.47%)
Cannot be classified	95 (60.50%)

### 3.2 Publication and citation trends

The annual scientific output on stay-green research in legumes reveals a clear upward trajectory in publication volume from 1993 to 2025 (Figure 2a). In the early years (1993–2011), there was minimal activity, with some years having fewer than two papers published annually. However, after 2013, there is a noticeable acceleration in the number of studies, culminating in a peak of 18 publications in 2023. This trend is further supported by the temporal evolution of source productivity (Supplementary Figure S3), indicating sustained growth in research output. The exponential trendline fitted to the data, with an R^2^ value of 0.80, indicates a moderately strong growth trend. This increase is likely due to rising global awareness about climate change, food insecurity, and the importance of developing drought-resilient crops. The stay-green trait, being associated with delayed leaf senescence and sustained photosynthetic activity under stress, has gained prominence as a strategic trait for improving legume productivity under adverse conditions. The growing publication rate suggests that this is an emerging area of research with expanding interest from the plant science and crop breeding communities. This growth is accompanied by a methodological shift from descriptive physiological studies toward molecular and genomic approaches including gene expression analysis, mutant analysis, transcriptome profiling etc. However, relatively few studies integrate field based confirmation, which limits the direct translation of these findings into breeding programs. This trend is further supported by the research area distribution (Figure 2c), which shows that most studies fall under *Plant Sciences* (35.40%), *Agronomy; Plant sciences* (15.04%), and *Agronomy* (12.39%) highlighting the trait’s physiological basis and its application in crop improvement. The involvement of additional fields such as *Biochemistry*, *Molecular Biology*, and *Genetics* highlights the multidisciplinary nature of stay-green research, reflecting a convergence of basic and applied sciences aimed at enhancing legume resilience to climate stress.

In the context of citation counts, there are notable citation spikes in specific years, particularly 2000 (717 citations), 2014 (708 citations), 2018 (258 citations), and 2021 (319 citations), indicating the presence of highly influential studies published in those years (Figure 2b). These historical citation peaks are clearly captured through Reference Publication Year Spectroscopy (RPYS), which highlights the foundation of stay-green research in legumes (Supplementary Figure S4). The older publications represent methodological breakthroughs, foundational reviews, and widely adopted breeding strategies related to stay-green research. For example, the work *“Five ways to stay-green”* by Thomas and Howarth (2000), published in the *Journal of Experimental Botany*, has received 710 citations to date, with an average of 27.31 citations per year, clearly marking it as a cornerstone in the field. These and other key studies are summarized among the most cited articles (Table 3) and influential references identified through co-citation analysis (Supplementary Table S3; Supplementary Figure S5). Similarly, *“Crops that stay-green”* (Thomas and Smart, 1993) and *“The stay-green trait”* (Thomas and Ougham, 2014) have accumulated 330 and 400 citations respectively, indicating both historical significance and contemporary relevance. The presence of these highly cited older articles not only validates the foundational status of early research but also shows that such work continues to underpin recent advancements. In contrast, more recent publications, especially those post-2014, such as Wang et al. (2018) with 231 citations, show relatively lower total counts. However, when adjusted for time, the publication maintains respectable annual citation rates of 28.88, showing promising influence despite limited time since publication. This trend aligns with expected bibliometric patterns, where newer articles naturally require time to accumulate citations.

**TABLE 3:** List of top 15 most cited articles related to the stay-green research in legumes, based on total citations, citations per year, journal source, and publication year.

Rank	Article title	Journal	Times cited	Publication year	TC per year	References
1	Five ways to stay green	Journal of Experimental Botany	710	2000	27.31	Thomas and Howarth (2000)
2	The stay-green trait	Journal of Experimental Botany	400	2014	33.33	Thomas and Ougham (2014)
3	Crops that stay green	Annals of Applied Biology	330	1993	10.00	Thomas and Smart (1993)
4	Parallel selection on a dormancy gene during domestication of crops from multiple families	Nature Genetics	231	2018	28.88	Wang et al. (2018)
5	Mendel’s green cotyledon gene encodes a positive regulator of the chlorophyll-degrading pathway	Proceedings of the National Academy of Sciences of the United States of America	209	2007	11.00	Sato et al. (2007)
6	Cross-Species Identification of Mendel’s I Locus	Science	190	2007	10.00	Armstead et al. (2007)
7	Improvement of crop yield in dry environments: benchmarks, levels of organisation and the role of nitrogen	Journal of Experimental Botany	163	2014	13.58	Sadras and Richards (2014)
8	ABI3 controls embryo degreening through Mendel’s I locus	Proceedings of the National Academy of Sciences of the United States of America	159	2013	12.23	Delmas et al. (2013)
9	From Model to Crop: Functional Analysis of a STAY-GREEN Gene in the Model Legume Medicago truncatula and Effective Use of the Gene for Alfalfa Improvement	Plant Physiology	144	2011	9.60	Zhou et al. (2011)
10	Development of cowpea cultivars and germplasm by the Bean/Cowpea CRSP	Field Crops Research	120	2003	5.22	Hall et al. (2003)
11	Mass Exodus from Senescing Soybean Chloroplasts	Plant and Cell Physiology	98	1999	3.63	Guiamét et al. (1999)
12	Genetic Architecture of Delayed Senescence, Biomass, and Grain Yield under Drought Stress in Cowpea	PLOS ONE	96	2013	7.38	Muchero et al. (2013)
13	Stay-green protein, defective in Mendel’s green cotyledon mutant, acts independent and upstream of pheophorbide a oxygenase in the chlorophyll catabolic pathway	Plant Molecular Biology	94	2008	5.22	Aubry et al. (2008)
14	What stay-green mutants tell us about nitrogen remobilization in leaf senescence	Journal of Experimental Botany	92	2002	3.83	Thomas et al. (2002)
15	Concerted evolution of D1 and D2 to regulate chlorophyll degradation in soybean	The Plant Journal	84	2014	7.00	Fang et al. (2014)

### 3.3 Journal analysis

The bibliometric analysis of journals publishing research on the stay-green research in legumes reveals both the breadth and impact of scholarly dissemination across diverse publication outlets. Table 4 ranks the top 15 journals based on the number of publications related to stay-green research in legumes. *Frontiers in Plant Science* emerged as the most prolific journal, publishing ten articles related to stay-green research (Supplementary Figure S6). However, despite its publication frequency, the journal accounted for only 289 total citations, resulting in a modest average of 28.90 citations per article. In contrast, the *Journal of Experimental Botany* (JXB), despite publishing only five relevant articles, exhibited the highest overall influence. The journal accumulated 1384 total citations, with a significant average of 276.8 citations per article, emphasizing its role in hosting foundational and highly cited research, particularly the seminal works of *Thomas and colleagues*. The journal’s strong impact factor of 5.7 and its frequent citation across top-ranked studies further establish JXB as a leading platform for conceptual and mechanistic research on senescence and stay-green phenotypes. This publication pattern is consistent with Bradford’s Law, which indicates that stay-green research in legumes is concentrated within a small core of journals that contribute most articles in this field (Supplementary Table S4; Supplementary Figure S7). The other journals demonstrating high citation impact per article include *Proceedings of the National Academy of Sciences of the United States of America* (PNAS) (124.33), *Plant and Cell Physiology* (65.0)*, The Plant Journal* (56.67)*, Plant Molecular Biology* (53.0). These journals, although publishing fewer articles, indicate a preference for publishing targeted, high quality research with long term scholarly value. Their strong performance is also reflected in competitive impact factors, such as 11.1 for PNAS and 5.7 for *The plant Journal*. This highlights their contribution to the genetic and molecular understanding of stay-green traits in legumes and other crop species. Therefore, this analysis indicates that while publication volume varies, journal prestige and subject alignment strongly influence citation performance. The journals that specialize in plant physiology, molecular biology, and crop genetics tend to host the most impactful stay-green related research.

**TABLE 4:** List of top 15 journals based on the number of publications related to stay-green research in legumes, alongside citation data, publisher details, and impact factors.

Rank	Journal	Publications	Citations	Average citations per article	Publisher	Publisher city	Impact factor
1	Frontiers in Plant Science	10	289	28.90	Frontiers Media SA	Lausanne	4.8
2	Agronomy	7	58	8.29	MDPI	Basel	3.4
3	Journal of Experimental Botany	5	1384	276.80	Oxford Univ Press	Oxford	5.7
4	Plant Physiology	5	219	43.80	Oxford Univ Press	Oxford	6.9
5	Crop Science	5	162	32.40	Wiley	Hoboken	1.9
6	Proceedings of the National Academy of Sciences of the United States of America	3	373	124.33	National Academy of Sciences	WashingtonD.C.	11.1
7	The Plant Journal	3	170	56.67	Wiley	Hoboken	5.7
8	The Crop Journal	3	130	43.33	Elsevier	Amsterdam	5.6
9	Physiologia Plantarum	3	112	37.33	Wiley	Hoboken	3.6
10	Canadian Journal of Plant Science	3	51	17.00	Canadian Science Publishing	Ottawa	1.3
11	Plant Cell & Environment	3	21	7.00	Wiley	Hoboken	6.3
12	Plant and Cell Physiology	2	130	65.00	Oxford University Press	Oxford	4.7
13	PLOS ONE	2	105	52.50	Public Library of Science (PLOS)	San Francisco	-
14	Plant Molecular Biology	2	106	53.00	Springer Nature	Dordrecht	4.6
15	South African Journal of Botany	2	79	39.50	Elsevier	Amsterdam	2.7

### 3.4 Co-authorship network analysis

**FIGURE 3:**
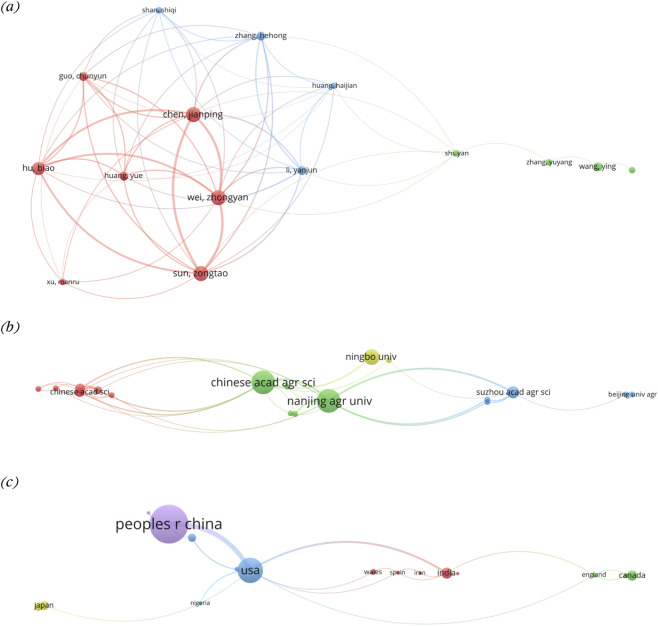
Co-authorship network analysis. The analysis was performed at three levels: **(a)** authors, **(b)** organizations, and **(c)** countries. The VOSviewer visualizations present clusters based on full counting method. For authors as the unit of analysis, the minimum number of documents per author was set to 2 (73 met the threshold; the largest set of connected items consists of 15 was depicted). For organizations as the unit of analysis, the minimum number of documents per organization was set to 2 (40 met the threshold; the largest set of connected items consists of 19 was depicted). For countries as the unit of analysis, the minimum number of documents per country was set to 2 (19 met the threshold; the largest set of connected items consists of 18 was depicted).

The co-authorship network analysis was performed to explore the collaborative relationships between authors, organizations, and countries involved in the field of stay-green research in legumes (Figures 3a–c). In each network diagram, each node (circle) represents individual author, organization, or country, with the node size corresponding to the number of publications associated with that entity. The connecting lines represent collaborative relationships, with the thickness of the lines indicating the degree of cooperation. The use of different colors differentiates various clusters of relationships.

The co-authorship network based on the author shows three prominent collaboration clusters (Figure 3a). The red cluster, led by Jianping Chen, includes key contributors like Zhongyan Wei, Zongtao Sun, Biao Hu, Yue Huang, Chunyun Guo and Manru Xu. This group forms a dense collaborative core with strong interconnections. Studies from this collaborative group have identified several mechanisms underlying pest induced stay-green symptoms. They identified multiple salivary effector proteins such as RpSP1 (Hu et al., 2025), Rp614 (Shan et al., 2023), Rp2155 (Huang et al., 2023) and RpSP27 (Wang G. et al., 2025) that interfere with soybean defense pathways and delay plant senescence. These effectors interact with host proteins and trigger immune responses, cell death and hormonal signaling changes (Hu et al., 2025; Huang et al., 2023; Shan et al., 2023), which ultimately leads to abnormal pod development and delayed maturation (Wang G. et al., 2025). The feeding by *Riptortus pedestris* suppresses flowering regulators such as FLOWERING LOCUS T (FT) gene family, linking pest infestation to the molecular regulation of plant development and senescence (Wei et al., 2023). These findings highlight how research on stay-green has moved beyond descriptive physiological observation towards mechanistic understanding of insect-plant interactions and molecular approaches for improving targeted pest resistance in legumes.

In terms of productivity, JP Chen further appears as the most prolific author with seven publications, and ZT Sun and ZY Wei follow with the same (Figure 2g). The highly cited and influential authors were identified through local citation analysis (Supplementary Figure S8), in which H. Thomas ranks highest, followed by JP Chen, ZT Sun and ZT Wei, further highlighting the central role of a limited number of researchers in shaping the field. JP Chen’s presence in the red cluster is emphasized by his continued activity from 2021–2025, with 2023 being his most productive year (citations >25) (Figure 2h). Likewise, H. Thomas, a foundational figure in stay-green research, was most active between 1993 and 2014, with peak citation years in 2000 and 2014 (>25 citations each). Further, the blue cluster is centered around Hehong Zhang, including collaborators such as Haijian Huang, Yanjun Li, and Shiqi Shan. Likewise, the green cluster involves Yuyang Zhang, Yan Shi and Ying Wang, representing a relatively smaller but well-connected group with independent research activity. The presence of dense intra-cluster collaboration (among JP Chen’s network) indicates strong thematic cohesion, often linked by shared research grants, institutions, or national projects. The bridging authors like JP Chen serve as key facilitators for cross-institutional and international collaboration, enabling the dissemination of methods and findings across groups.

In organizational context, the network map reveals domestic collaborations among key Chinese research institutions (Figure 3b). There are four prominent clusters: the green cluster with *Chinese Academy of Agricultural Sciences* (CAAS) and *Nanjing Agricultural University* (NAU) showing extensive collaboration other regional institutions; the blue cluster with NAU, maintains active partnerships with *Suzhou Academy of Agricultural Sciences*, *Beijing University of Agriculture* and more; the red cluster, centered around *Chinese Academy of Sciences*, *Shanxi Agricultural University, and Northwest A and F University* shows regionally focused collaboration within northwest China; and yellow cluster with Ningbo University, Henan Agricultural University, and Anhui Academy of Agricultural Sciences. This findings indicate a strong national collaborative framework within China, with CAAS and NAU acting as a central institution facilitating inter university research. Further, this collaborative dominance is further supported by publication output data, which shows that the NAU produced the highest number of articles (17), followed by the *CAAS*, contributing 14 publications (Supplementary Figure S9), with sustained research output over time (Supplementary Figure S10). The alignment between high research output and central positioning in the collaboration network suggests that these institutions not only act as research hubs but also drive the national research agenda in the field of stay-green research. This pattern reflects the dominance of Chinese institutions and highlights China’s leading role in stay-green research.

The country wise scientific production data further confirm China’s leading role in publication output, followed by the United States, Japan, and India (Supplementary Figure S11). The United States (USA) (blue cluster) and People’s Republic of China (purple cluster) are the most prolific countries in stay-green research, forming the largest bilateral collaboration. India, Japan, Wales, Nigeria, Canada, and England exhibit moderate co-authorships with either USA or China (Figure 3c). Nigeria and Japan appear as peripheral nodes, with limited but noteworthy collaborations. The strong USA-China axis is the hallmark of high-impact international research. This reflects both countries' investment in legume crop improvement and molecular breeding. This collaboration pattern is further supported by the spatial distribution and citation metrics of published research. China leads in total output, with 40+ publications, followed by the USA, Canada, and India (Figure 2d). China and USA’s strong performance reflect their investment in legumes research and crop improvement programs. However, when considering citation impact, the UK ranks highest with 1,304 citations, surpassing both China (881) and the USA (509) (Supplementary Figure S12), emphasizing UK’s foundational influence despite fewer publications. Therefore, the data suggests both developed and developing nations are engaged in stay-green research, but with especially high momentum in the Global South. However, the limited connections with countries like Nigeria and Japan point to opportunities for more inclusive and diversified collaboration, especially in legume producing regions of Africa and East Asia.

### 3.5 Keyword co-occurrence and hotspot analysis

**FIGURE 4:**
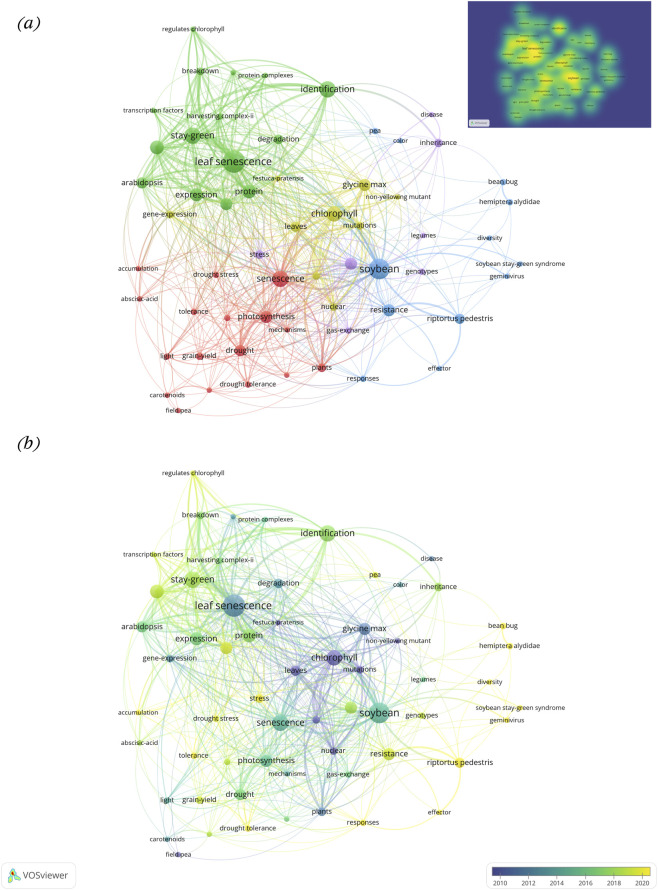
**(a)** Keyword co-occurrence network analysis; **(b)** Keyword co-occurrence overlay visualization depicting temporal evolution. For this analysis, the unit of analysis was selected as “All keywords,” which includes the author keywords and KeyWords Plus, and the counting method used was full counting. The minimum number of occurrences of a keyword was set to 4 (60 met the threshold). The top right image shows a keyword density visualization, where warmer colors (yellow to green) indicate regions with a higher concentration of frequently co-occurring keywords, while cooler colors represent less intensively studied areas.

Out of a total of 829 keywords, the top 10 dominant terms were “leaf senescence (24)”, “soybean (22)”, “stay-green (16)”, “identification (16)”, “senescence (16)”, “chlorophyll (15)”, “chlorophyll degradation (13)”, “expression (11)”, “protein (13)”, and “expression (12)” (Figure 2e; Supplementary Table S2). The Keyword co-occurrence analysis resulted in the formation of five distinct clusters, each color cluster representing a key research hotspot (Figure 4a). The supporting network metrics obtained from VOSviewer and bibliometrix are provided in the Supplementary Material (Supplementary Table S5; Supplementary Table S6; Supplementary Figure S13). The cluster information along with the research hotspots are described below:

1. The red cluster highlights drought tolerance and plant physiological responses, representing one of the primary research focuses on stay-green research in legumes. This cluster includes keywords such as drought, drought stress, growth, grain-yield, photosynthesis, nitrogen, senescence, tolerance etc. The research in this domain primarily explores how stay-green traits contribute to improved drought tolerance, particularly through delayed senescence and sustained photosynthetic activity (Thomas and Howarth, 2000). The physiological and agronomic effects of drought stress, including nitrogen assimilation, leaf water status, and biomass retention, are frequently studied. Further, the role of stay-green phenotypes in maintaining grain yield and productivity under abiotic stress has been especially emphasized in cereals like sorghum and maize (Borrell et al., 2014), with increasing interest in legumes like soybeans and beans. However, in legumes, most supporting evidence is obtained from controlled environment or adapted from cereal based systems which limit the field based validation under diverse agro-ecological conditions, restricting broader applicability.
2. The green cluster area highlights molecular regulation and leaf senescence mechanisms, another central research focus in stay-green research. The keywords in this cluster include stay-green, leaf senescence, transcription factors, chlorophyll degradation, genes, protein complex, breakdown etc. This body of research investigates the genetic and molecular pathways that regulate leaf senescence and chlorophyll retention. The regulatory networks involving transcription factors such as NAC, WRKY, and bZIP families play key roles in modulating leaf senescence in crops like wheat, maize, sorghum, and rice (Cao et al., 2023). Genes like *SGR* (Stay-Green), *NYC1*, and others have been identified as central players in chlorophyll catabolism (Sakuraba et al., 2012). This cluster highlights the growing interest in using reverse genetics, transcriptome profiling, and mutagenesis to dissect senescence related gene function in legumes. However, a significant proportion of research findings are based on model species or association studies that limit gene characterization in legumes directly.
3. Likewise, the yellow cluster area draws attention to the photosynthesis-related genetic and proteomic processes. This cluster includes keywords such as chlorophyll, proteins, nuclear, mutations, glycine max, gene expression, leaves etc. It represents studies focused on biochemical and genetic mechanisms underpinning chlorophyll synthesis and stability. The identification of stay-green mutants both functional and non-functional in model species like Arabidopsis and their extrapolation to crops like soybeans is a key theme (Thomas and Smart, 1993; Delmas et al., 2013). These studies aim to comprehend how photosynthetic capacity, pigment concentration, and thylakoid protein interactions contribute to prolonged greenness and productivity under stress. Though several studies fail to distinguish the basis of chlorophyll retention, whether it is linked with sustained photosynthetic efficiency or impaired degradation processes, showing the need for inclusive physiological and molecular validation approaches.
4. The purple cluster signifies the focus on trait mapping, genomic selection, and inheritance studies. The keywords such as genotypes, inheritance, disease, stress, and legumes, form the core of this cluster. This research focuses on mapping of stay-green associated quantitative trait loci (QTLs), expression profiling under biotic and abiotic stress, and gene discovery using tools such as genome-wide association studies (GWAS) and marker-assisted selection (MAS). Stay-green QTLs have been widely reported in sorghum, maize, and increasingly in legumes like soybean and chickpea (Sivasakthi et al., 2019; Faye et al., 2022; Rama Reddy et al., 2014; Kamal et al., 2019; Zhang M. et al., 2022). This hotspot also includes studies exploring how stay-green traits behave under both abiotic and biotic stress conditions, helping breeders identify stable lines with desirable senescence patterns.
5. The blue cluster highlights an emerging research theme focused on biotic stress interactions and disease associated stay-green syndromes, particularly in soybean. The keywords in this cluster include soybean stay-green syndrome, geminivirus, riprotus pedestris, bean bug, resistance, and responses. This research addresses the causes and effects of abnormal stay-green phenotypes induced by viral infections or insect pests. Soybean stay-green syndrome, often associated with geminivirus infection or bean bug feeding, leads to delayed senescence and leaf retention without a corresponding yield benefit, reflecting a non-functional stay-green condition (Li et al., 2019; Rolando et al., 2025; Wang et al., 2022; He et al., 2024). The studies in this cluster emphasize the importance of distinguishing between desirable (functional) and undesirable (pathological) stay-green phenotypes during cultivar selection and breeding.

### 3.6 Keyword co-occurrence temporal overlay analysis

In the case of keyword co-occurrence timeline analysis, the color scale indicates the average publication year for each keyword. The temporal trends include older topics (∼2010, blue), emerging topics (∼2012–2016, green) and very recent topics (∼2018–2020, yellow) (Figure 4b). The early stage research, indicated by blue toned keywords such as chlorophyll, nuclear, proteins, mutations, non-yellowing mutants etc., reflects foundational biological and genetical studies of stay-green traits. From around 2010 onwards (green toned), there is a noticeable shift toward terms like photosynthesis, senescence, growth, leaf senescence, disease, drought, drought stress, chlorophyll degradation, genotypes, yield etc. This shift signifies a methodological transition from biological studies to trait improvement. The presence of both trait-based and stress-related keywords suggests that the research trend is largely focused on understanding how stay-green contributes to stress tolerance and yield stability. Over time, the research focus has evolved towards the genetic and molecular regulation of stay-green, as evident in light green (2015 onwards) colored keywords like transcription factors, protein breakdown, chlorophyll degradation, gene expression, stay-green, protein complexes, regulates chlorophyll etc. These terms represent studies conducted predominantly in the last decade, where advanced tools such as QTL mapping, transcriptomics, and mutant analysis have been used to dissect the regulatory networks controlling chlorophyll retention and senescence delay. The similar keywords trends have been observed in keywords trend analysis (Figure 2f; Supplementary Table S7). The most recently emerging hotspots are marked in yellow on the overlay (2020 onwards), which include keywords such as soybean stay-green syndrome, riptortus pedestris, geminivirus, pea, bean bug, drought tolerance, stress, germination etc. These terms reflect newer investigations into stress related abnormal greenness, particularly in soybean, where virus or pest infection leads to pathological stay-green symptoms without agronomic benefits. This shift in research interest indicates a growing awareness of the need to distinguish between functional and non-functional stay-green expression, especially when integrating this trait into breeding programs. This transformation indicates maturity in the research field and a pursuit of more precise and scalable applications for legume breeding. The relevant publications related to functional and non-functional stay-green research are summarized in Table 5.

**TABLE 5:** Summary of relevant publications on stay-green research and associated molecular mechanisms in legumes and related crops extracted from bibliometric analysis.

Study type	Crop species	Trait/Gene or molecular target studied	Gene type or functional category	Types of mutation or genetic variation	Methods/Tools used	Stage of trait expression	Key findings/Trait associations	Application or implication	Publication year	Country*	References
Functional stay-green											
Transcriptome and physiological analysis	Soybean	GmSGR1, GmSGR2, GmSPS1, GmAGP1, GmUGP1-1, GmPDF1.2, GmJAR1.1	STAY-GREEN, Jasmonic acid (JA) pathway, photosynthesis	-	Gene expression, physiological assays, hormone profiling	Leaf senescence under heat and low light	Delayed senescence, improved photosynthesis and stress tolerance	Insight for improving heat-resilient soybeans via light modulation	2021	China	Bawa et al. (2021)
Gene expression profiling	Soybean	SGR3b and senescence-related transcription factors	SGR3b	Expression under sink-limitation (non-mutagenic)	RNAseq, gene ontology analysis	Post-flowering/senescence phase	Expression of stay-green gene SGR3b under sink-limitation	Understanding resource remobilization	2017	United States	Brown and Hudson (2017)
Mutant development, physiological and molecular analysis	Cowpea	Stay-green genotype, VunP5CS, VubZip09	Regulatory genes (transcription factors), stress-responsive genes	Gamma ray-induced mutations (mutant lines)	Gamma mutagenesis, physiological assays (chlorophyll, relative water content), biochemical assays (enzyme activities), RT-qPCR gene expression	Vegetative and drought stress stage	Identification of stay-green and early flowering drought-tolerant mutants; increased antioxidant enzyme activity and stress gene expression in tolerant lines	Genetic dissection of drought resilience for cowpea breeding programs	2024	Sénégal	Diallo et al. (2024)
Physiological drought response analysis	Soybean	Stay-green phenotype (physiological traits: CTD, chlorophyll)	Physiological trait-based (non-molecular)	-	Thermal imaging, chlorophyll measurement, canopy greenness indices	Reproductive stage	CTD and greenness correlated with seed yield under drought	Selection tool for drought-adaptive soybean genotypes	2017	India	Kumar et al. (2017)
Gene functional analysis and hormone signaling study	Soybean	At-ore1, GmNAC081, GmNAC065, GmNECD3, GmSAG39	Transcription factors, hormone biosynthesis and senescence-associated genes	Mutant lines (ore1-1, ore1-6), gene overexpression/suppression	Gene expression profiling, hormone assays, phenotyping, mutant analysis	Early vegetative stage to late senescence	ABA-induced early senescence via GmNAC081; IAA-induced stay-green via GmNAC065; delayed senescence increases yield	Genetic modulation of hormone pathways to control leaf senescence and enhance yield in soybean	2022	Vietnam	La et al. (2022)
GWAS and natural variation analysis	Common bean	Stay-green trait; hormone signaling, antioxidant production, trehalose synthesis	Multigenic (variants near gene models related to drought/stay-green response); Chr 1, 3, 5, 7, 8, 9, 10; 29 windows covering 1.45 Mb	SNP variants identified via GWAS	Phenotyping under drought, GWAS, FST (Fixation index) population differentiation, whole-genome resequencing	Functional stay-green under terminal drought	6 SG cultivars identified; strong genetic signals on Chr 1, 5, 10; genes linked to photosynthesis and trehalose synthesis	Identification of stay-green markers for breeding drought-resilient common beans	2023	Sweden	Labastida et al. (2023)
Physiological and biochemical study	Soybean	Leaf senescence, photosynthetic efficiency	Nutrient stress physiology	No mutation (physiological variation)	Gas exchange analysis, biochemical assays	Reproductive stage	Leaf N spraying delayed senescence, improved photosynthesis, biomass accumulation, and seed yield under P deficiency	Identification of physiological markers of senescence	2025	Brazil	Laira et al. (2025)
Field ecophysiology and cropping systems analysis	Maize and Soybean	Stay-green physiological trait under cropping pattern and nitrogen interaction	Not gene-level; trait-level physiological analysis	-	Field trials, Leaf Area Index (LAI), yield measurement, nitrogen treatment, phenology monitoring	Post-anthesis to maturity (lag phase)	Extended leaf stay-green period (4–8 days), increased LAI and photosynthesis, improved yield in both maize and soybean	Optimizing cropping systems and nitrogen input for sustainable yield enhancement using physiological stay-green traits	2023	China	Li et al. (2023b)
Transgenic functional genomics study	Peanut	PDH45 helicase	Stress-responsive DNA helicase	Overexpression (transgenic)	RT-PCR, transgenic analysis, drought stress assay	Drought stress, post-flowering	Stay-green phenotype, improved productivity	Drought tolerance breeding in peanut	2014	India	Manjulatha et al. (2014)
QTL mapping and association analysis	Cowpea	Stay-green QTLs (Dro-1, Dro-3, Dro-7)	Quantitative trait loci (QTL); Chromosome regions (3.2 cM resolution)	SNP variation	Illumina GoldenGate SNP genotyping, association mapping, bi-parental QTL mapping	Seedling and post-flowering stages	Identification of 7 loci linked to stay-green, biomass, and yield under drought; positive pleiotropy of stay-green with grain yield	Identifies marker-assisted breeding targets for drought tolerance in cowpea and enables rapid screening for post-flowering drought resilience	2013	United States	Muchero et al. (2013)
Agronomic performance and palatability evaluation study	Multiple (*Aeschynomene histrix*, *Centrosema sps.*, *Chamaecrista rotundifolia*, *Stylosanthes sps.)*	-	-	-	Field trial, palatability testing, agronomic measurements	Seasonal (dry and wet season)	Ability to retain green leaves in dry season, palatability, dry matter yield	Understanding of legume-based pasture management, legume mixtures for crop/livestock systems	2000	Colombia	Peters et al. (2000)
Phenotyping and multivariate statistical analysis	Cowpea	Stay-green trait associated with drought tolerance (phenotypic level)	-	-	Wooden box technique (high-throughput phenotyping), PCA, k-mean clustering, agronomic trait scoring	Seedling stage	Identification of drought-tolerant landraces showing stay-green traits; traits like stem greenness and leaf senescence correlated with drought response	Selection of drought-tolerant cowpea landraces for breeding in drought-prone ecologies	2024	Nigeria	Sakariyahu et al. (2024)
Phenotypic screening and agronomic evaluation	Common bean	Stay-green trait (chlorophyll a and b, delayed senescence)	Physiological stay-green (delayed chlorophyll degradation)	Natural genetic variation	Visual scoring, chlorophyll assay	Senescence (75–95 DAS)	Identified genotypes with delayed senescence and higher yield	Selection of drought/disease-resilient common bean lines	2019	Brazil	Schmit et al. (2019)
Transgenic functional genomics study	Groundnut (*Arachis hypogaea*)	MuNAC4, MuWRKY3, MuMYB96	Transcription factors	Overexpression (multigene)	qRT-PCR, physiological assays, SEM, antioxidant enzyme activity	Drought stress (vegetative to reproductive)	Improved water status, chlorophyll retention, root growth	Potential targets for breeding drought resilient stay-green legumes	2022	India	Venkatesh et al. (2022)
Mutant analysis and physiological characterization	Soybean	SGR1, SGR2	Stay-green gene (SGR1 mutation); Chr1 and Chr11 | 271 aa (wild-type), 229 aa (mutant)	Single base deletion causing exon skipping and 42-aa loss	Chlorophyll fluorescence, qPCR, sequencing, physiological assays	Senescence (podding to maturity)	Delayed leaf senescence, higher photosynthetic efficiency, better yield	Soybean breeding and germplasm innovation using stay-green trait	2021	China	Wang et al. (2021a)
Functional genomics and transgenic analysis	Soybean	GmbHLH3	bHLH transcription factor; jasmonic acid (JA) signaling regulator; senescence-associated regulator	Overexpression (transgenic overexpression of GmbHLH3 in Arabidopsis)	Gene cloning, transgenic overexpression, qRT-PCR, promoter cis-element analysis, yeast two-hybrid, bimolecular fluorescence complementation (BiFC), chlorophyll and senescence gene expression analysis	Vegetative growth and leaf senescence stage	GmbHLH3 inhibits JA-induced leaf senescence by suppressing senescence-associated genes and chlorophyll degradation genes; promotes root growth and alters flowering time under long-day conditions	Provides molecular insight into JA-regulated senescence control and potential genetic targets for manipulating stay-green traits in soybean	2020	China	Wang et al. (2020a)
Physiological and molecular analysis	Soybean	GmSARK, GmSGR1, GmCYN1, GmNAC, GmFT2a, E1	Senescence-related genes, flowering genes	-	Pod removal and seed injury treatments, gene expression analysis (qRT-PCR), chlorophyll measurement (SPAD), hormone quantification	Leaf senescence stage	Delayed leaf senescence (stay-green phenotype) after pod removal/seed injury; altered gene expression and hormone levels	Understanding source-sink regulation and senescence signaling with potential for crop improvement via stay-green trait management	2016	China	Zhang et al. (2016)
Genetic analysis and phenotyping study	Groundnut (*Arachis hypogaea*)	Stay-green trait, LAUG, SPAD, leaf spot tolerance	Physiological and genetic trait (stay-green)	Single recessive gene	Field evaluation under diseased and disease-free conditions, SPAD, LAUG, correlation analysis	Reproductive stage (leaf spot disease phase)	Strong correlation between stay-green (LAUG) and leaf spot tolerance; stay-green genotypes showed disease resistance and higher yield	Selection of leaf spot tolerant ground nut using stay-green trait	2019	Ghana	Danful et al. (2019)
Gene functional analysis and molecular signaling study	Soybean	GmCRY1s, GmDELLAs, GmWRKY100	Light signaling, transcription factors, senescence regulators	Gene knockout (GmWRKY100 knockout lines)	Gene interaction assays, knockout studies, molecular analysis	Leaf senescence stage under shading (low blue light)	GmCRY1s-DELLA-WRKY100 pathway regulates senescence; knockout delayed senescence and improved yield	Target for improving yield via light-regulated senescence control	2024	China	Li et al. (2024)
Transgenic functional genomics study	Groundnut (*Arachis hypogaea*)	PG47 RNA helicase	Stress-responsive helicase gene	Overexpression (transgenic lines)	PCR, RT-PCR, Southern blot, stress assays	Drought and stress conditions (vegetative to reproductive)	Enhanced stay-green phenotype, chlorophyll stability, and yield under stress	Genetic engineering for drought tolerance and yield improvement	2018	India	Mahantesh et al. (2018)
Genetic segregation and breeding study	Soybean	Delayed leaf senescence (DLS), SPAD, canopy temperature	Physiological stay-green trait	Transgressive segregation in F2 populations	Field trials, statistical analysis	Drought stress stage	Identification of superior transgressive segregants with improved physiological traits	Breeding drought-tolerant soybean genotypes	2025	India	Nair et al. (2025)
Mutant analysis and physiological characterization	Soybean	Stay-green mutant Z1, antioxidant enzyme genes (SOD, CAT, MDHAR, DHAR)	Antioxidant system and senescence regulation	Hybrid from stay-green mutant × cultivar	qPCR, enzyme assays, ROS analysis, dark treatment	Seedling to seed-filling and dark-induced senescence	Enhanced antioxidant capacity delays senescence and maintains chlorophyll	Use of stay-green mutants for soybean breeding and stress tolerance	2021	China	Wang et al. (2021b)
Non-functional stay-green											
Physiological trait analysis	Common bean (*Phaseolus vulgaris*)	Chloroplast degradation pathway	Stay-green mutants (not molecularly identified)	Induced or natural (not detailed)	Protein profiling, SDS-PAGE, Western blot	Pod-filling stage	Retention of chloroplast proteins in mutants during senescence	Genetic variability source for improving photosynthetic duration	1994	United Kingdom	Bachmann et al. (1994)
Gene discovery and functional analysis	Pea (*Pisum sativum*)	SGRL (SGR-like)	SGR homolog	-	Transient expression, mutant analysis	Development and senescence	SGRL promotes chlorophyll recycling distinct from SGR	Target for manipulating stay-green and yield traits in pea	2015	United Kingdom	Bell et al. (2015)
Genetic, physiological and biochemical study	Soybean	Stay-green mutations cytG and Gd1d2 affecting chlorophyll breakdown	Structural genes involved in chlorophyll degradation pathways	Natural stay-green mutations (cytG and Gd1d2)	Seedling morphological analysis, chlorophyll quantification, light/dark growth experiments	Seedling and early development stage (etiolation and light response)	Inhibition of chlorophyll breakdown in cotyledons and leaves; altered chlorophyll a/b ratios; altered light-induced chlorophyll degradation	Understanding regulation of chlorophyll degradation for potential crop improvement	1995	United States	Canfield et al. (1995)
Gene discovery and functional analysis	Faba bean	VfSGR (pea SGR homolog)	SGR homolog; SNP at CDS position 513	SNP; pre-stop codon	dCAPs marker, transient expression, dark treatment	Senescence, dark-induced senescence	Green cotyledons, delayed senescence	Marker-assisted breeding of green-cotyledon faba beans	2023	China	Chen et al. (2023)
Physiological and genetic characterization	Soybean	Chlorophyll content and senescence traits	Physiological senescence trait	Natural phenotypic variation	Phenotypic analysis, physiological measurements	Seed maturation/cotyledon stage	Variation in chlorophyll retention associated with senescence behavior	Phenotypic screening for stay-green traits	2021	South Korea	Choi et al. (2021)
Genetic trait functional analysis	Snap bean (*Phaseolus vulgaris*)	Persistent color (pc) - cosmetic stay-green gene family	Cosmetic stay-green gene (pc); Pv02, Pv07	Recessive mutation	Tetrazolium test, Electrical conductivity (EC), seed anatomy, field and lab germination	Seed maturation and germination	Pc lines show reduced field germination, higher pathogen susceptibility	Breeding for improved seed vigor; pod quality enhancement	2021	United States	Cirak and Myers (2021)
Gene functional analysis	Arabidopsis, Soybean, Pea	ABI3, SGR1	Transcription factor (ABI3), Chlorophyll degradation (SGR1); SGR1: At4g22920	Loss of function (abi3-6, SGR1 mutation)	Mutant analysis, transcriptomics, misexpression	Seed maturation	Green seeds due to failure in embryo degreening	Solution to green seed problem in oilseed crops	2013	Canada	Delmas et al. (2013)
Transcriptomic and gene expression profiling	*M. truncatula*	Senescence-associated genes (SAGs), transcription factors (MYB, WRKY, bHLH, NAC)	Transcription factors and SAGs	Not mutation-based; transcriptome-level expression change	High-throughput RNA-seq, GO/KEGG enrichment analysis	Leaf senescence under alkaline stress and dark-induced conditions	Identified 2,165 DEGs, 985 of which overlaped with dark-induced senescence genes, involvement in nutrient cycling and stress response	Candidate genes for breeding stay-green, high-yield forage crops with tolerance to alkaline stress	2022	China	Dong et al. (2022)
Molecular and evolutionary analysis	Soybean	D1 and D2 (SGR homologs)	Paralogous nuclear genes	Retrotransposon insertion (GmD2IN) causing d2 mutation	Gene cloning, transcriptional profiling, mutant analysis, genome evolution analysis	Senescence (older tissues)	Double mutant (d1d1d2d2) shows chlorophyll retention (stay-green); retrotransposon insertion leads to mutation	Understanding stay-green regulation and genome evolution, supporting breeding of stay-green soybean varieties	2014	China/United States	Fang et al. (2014)
Physiological and biochemical study	Soybean	Nuclear stay-green loci (d1, d2, G genes)	Nuclear genes	Recessive d1, d2 mutations	Electron microscopy, chlorophyll assays, hormone treatments	Senescence stage	Mutants d1d1d2d2 and GGd1d1d2d2 inhibit thylakoid and plasma membrane degradation during leaf senescence; hormone treatments did not reverse stay-green phenotype	Provides a tool to study senescence biochemistry and leaf aging regulation	1994	Argentina	Guiamét and Giannibelli (1994)
Mutant analysis	Soybean	dl, d2, cytG	Nuclear and cytoplasmic STAY-GREEN genes	Recessive alleles and cytoplasmic gene	Physiological assays, protein quantification	Leaf senescence	Delayed Rubisco degradation, high protein retention	Insight into regulatory control of senescence and protein degradation	1996	Argentina	Guiamét and Giannibelli (1996)
Cytological and physiological study	Soybean	Stay-green phenotype (GGd1d1d2d2)	Nuclear stay-green genotype (d1/d2) inhibiting chloroplast degradation	GGd1d1d2d2 mutant	Microscopy, fluorescence, biochemical assays	Leaf senescence	Chloroplast degradation via plastoglobuli blebbing	New model for chlorophyll breakdown in stay-green mutants	1999	United States	Guiamét et al. (1999)
Mutant analysis and physiological characterization	Soybean	cytG (stay-green mutation)	Stay-green gene (affecting protein degradation pathways)	Maternally inherited mutation cytG	Protein analysis (LHCII, PSII, ATP synthase), oxygen evolution activity, light/dark senescence induction, lincomycin inhibition	Monocarpic and dark-induced senescence	cytG delays LHCII/chlorophyll degradation but enhances photoinhibition and PSII protein loss in light-exposed senescent leaves	Demonstrates separable pathways for thylakoid component degradation and provides insights into light-dependent senescence in stay-green mutants	2002	United States	Guiamét et al. (2002)
Population genetics/genetic diversity analysis	Soybean	green cotyledon + black seed coat	-	Natural variants/alleles (d1d2, psbM)	6K SNP genotyping; diversity/structure analyses; chlorophyll content comparison; core collection selection	Seed maturation stage	Narrow variability in Korea; d1d2 higher Chl a than psbM; 36 accession core collection	Breeding materials as a core collection	2021	South Korea	Jo et al. (2021)
Genetic and molecular study	Soybean	cytG gene encoding PsbM protein	Chloroplast gene	Frameshift mutation (5-bp insertion)	Chloroplast genome sequencing, chloroplast transformation in tobacco, biochemical assays	Leaf senescence and seed maturation	cytG mutation disrupts chlorophyll degradation causing stay-green phenotype; links photosynthesis with chlorophyll degradation during senescence	Insights into chlorophyll degradation and soybean domestication, highlighting potential breeding target for stay-green traits	2017	Japan	Kohzuma et al. (2017)
Physiological characterization	Soybean	Stay-green genotype Gd1d2 (d1d2) affecting chlorophyll retention during senescence	Nuclear stay-green loci (d1/d2) with G; pleiotropic regulation	Recessive alleles d1d1d2d2 + G (near-isogenic mutant genotype)	Photosynthetic and biochemical measurements	Post-flowering and senescence stages	Preserved machinery but photosynthesis similar; lower stomatal conductance; yield reduced 10%–20%	Shows limitations/pleiotropy of stay-green genotype for yield gains outdoors	2001	Argentina	Luquez and Guiamét (2001)
Genetic and physiological analysis	Soybean	Stay-green mutations: d1 and d2; gene G (chlorophyll preservation)	Nuclear stay-green loci (d1/d2, G) regulating chloroplast degradation and stress response	Natural mutations	Physiological measurements (leaf water potential, stomatal conductance), mutant comparison, drought stress assays	Senescence stage	Increased susceptibility to water deficit; delayed chloroplast breakdown; altered water relations during senescence	Understanding about trade-offs of the stay-green phenotype with implications for drought tolerance breeding	2002	Argentina	Luquez and Guiamét (2002)
Genetic mapping and phenotypic analysis	Pea (Pisum sativum)	Green cotyledon (seed color) trait related to chlorophyll and carotenoid retention	Seed chlorophyll retention/cotyledon pigmentation QTLs	Natural genetic variation	HPLC, QTL mapping, Genetic linkage mapping, Image analysis	Seed development and seed maturation	Multiple QTLs control green cotyledon color through differences in pigment loss and chlorophyll-carotenoid composition during seed maturation	Genetic resources and QTL information for breeding pea varieties with desired seed color and quality traits	1997	New Zealand	McCallum et al. (1997)
Genetic, genomic, mutant characterization	Soybean	Stay-green/green-cotyledon trait; GmSGR1, GmSGR2 (D1 and D2) genes	Stay-green genes from two duplicated loci; Natural mutant line Tenshin-daiseitou, RNAi transgenic soybeans	-	Whole-genome sequence analysis, gene mutation analysis, RNA interference, physiological assays	Senescence stage	Mutations in duplicated SGR genes (GmSGR1 and GmSGR2) cause stay-green phenotype; evidence of ancient whole-genome duplications generating paralogs; seed-specific chlorophyll composition differences observed	Provides molecular evidence linking stay-green genes to soybean genome duplications, aiding genome evolution studies and functional genomics for crop breeding	2014	Japan	Nakano et al. (2014)
Image-based drought stress phenotying study	Common bean	Stay-green phenotype (image-based monitoring)	-	-	High-throughput imaging, RGB sensors	Post-flowering under drought stress	Visual senescence differentiation, drought-induced stay-green	Non-invasive screening of drought responses	2019	Mexico	Padilla-Chacón et al. (2019)
Transgenic functional genomics study	Soybean	GmSGR1	SGR1; 3621 bp, 261 aa	Overexpression and transgenic analysis	Gene cloning, transgenic tobacco, GUS assay	Senescence, nitrogen deficiency	Early yellowing, lower biomass, PSII efficiency decline	Elucidates the regulatory role of GmSGR1 in chlorophyll degradation and senescence signaling	2016	China	Shi et al. (2016)
Gene functional analysis and phenotypic dissection	Chickpea (*Cicer arietinum* L.)	CaStGR1 (ortholog of Mendel’s I locus, SGR gene)	Stay-green gene, SGR protein ortholog; 5 allelic variants	Loss-of-function alleles in CaStGR1	Introgression lines, sequencing, physiological assays, pigment quantification, agronomic evaluation	Seed maturation and cotyledon development	Increased provitamin A carotenoids (2-3x), no effect on transpiration efficiency or agronomic traits	Carotenoid biofortification without compromising agronomic performance or environmental adaptation	2019	India/United States	Sivasakthi et al. (2019)
Gene expression profiling	Soybean	SGR1, SGR2, PPH2, NYC1-1	STAY-GREEN, chlorophyll degradation	-	Expression profiling (qRT-PCR), stress treatment	Mature seeds under drought and heat	Green seed phenotype linked to reduced expression of chlorophyll-degradation genes	Breeding for heat/drought-tolerant soybean with low green seed risk	2016	Brazil/Netherlands	Teixeira et al. (2016)
Functional genomics study	Soybean	G and GL (G-like)	Regulatory gene for chlorophyll degradation	Natural alleles at G and GL loci (G/g; GL/gl)	Transient expression, GFP fusion, near-isogenic lines, chlorophyll assay	Mature seeds (seed coat and cotyledons)	Chlorophyll retention (green seeds) vs. yellow seed coat	Selection of soybean cultivars with desired seed coat color	2022	Japan	Tokumitsu et al. (2022)
Physiological and molecular analysis	Pea (Pisum sativum)	Post-harvest cotyledon bleaching resistance	Seed visual quality trait	Natural genetic variation	Physiological pigment assays; carotenoid:chlorophyll ratio; oligonucleotide microarray (Ps6kOLI1); accelerated bleaching exposure	Seed development and post-harvest stage	CDC Striker shows slower chlorophyll degradation, a higher carotenoid to chlorophyll ratio, altered seed coat gene expression, and increased expression of antioxidant secondary metabolite genes	Candidate genes for future validation and marker development for bleaching resistance/seed visual quality	2013	Canada	Ubayasena et al. (2013)
Transcriptomic analysis and genetic transformation study	Soybean	GmSGRs, chlorophyll degradation pathway genes	Stay-green/chlorophyll catabolism (SGR)	Gene knockdown (RNAi)	RNA-Seq (Illumina Solexa), genetic transformation, RNA interference	Seedling, flowering, maturation	Identification of key genes (MDCase, PAO, GmSGRs) regulating stay-green; GmSGRs knockdown delays senescence	Provides foundation for genetic improvement to delay aging and improve yield	2020	China	Wang et al. (2020b)
Transcriptomic and physiological pathway analysis	Soybean	Photosynthesis, starch/sucrose metabolism, hormone signaling (GmBAM5, GmSS2, α-glucosidase, β-mannosidase)	Metabolic and hormone-regulated genes	Not mutation-based; gene expression variation due to SGS	Tissue-specific RNA-seq, chlorophyll quantification, sugar/hormone analysis	Pod and leaf development under SGS conditions	320% and 260% increase in chlorophyll a and b in SGS pods, respectively; increased sugar levels in leaves, decreased IAA and ABA in SGS pods; key starch and photosynthesis genes upregulated	Understanding and potential management of SGS to improve soybean yield	2025	China	Wang et al. (2025c)
Gene functional analysis and molecular characterization	*M. truncatula*	Non-yellow Coloring 1 (MtNYC1), chlorophyll b degradation pathway	Structural gene (chlorophyll b reductase); Gene includes 3 transmembrane domains and catalytic motif; splicing variants encode full-length and truncated proteins	Tnt1 retrotransposon insertion mutants; natural alternative splicing	Tnt1 mutant screening, gene cloning, transcript analysis (splicing variants), sequence motif analysis, physiological phenotyping	Leaf senescence; seed maturation	Stay-green phenotype in mutants (delayed chlorophyll b degradation); identified functional domains essential for activity	Targeted breeding for stay-green traits in legumes and precise molecular targets for manipulating leaf senescence	2025	China	Wang et al. (2025a)
Multi-omics plant pathology study	Soybean (*Glycine max*)	Not a specific plant gene; novel geminivirus	Viral	-	Metagenomics, microbiome sequencing, virome profiling	Whole plant including seeds, pods, stems, roots	Stay-green syndrome linked to seed dysbiosis and novel geminivirus	Potential biomarker and causal agent for SGS diagnosis and management	2022	China	Wang et al. (2022)
Genetic and molecular analysis	Soybean	Stay-green-related loci	Nuclear stay-green genes (d1, d2) controlling chlorophyll degradation	QTL-based genetic variation	QTL mapping, gene characterization	Reproductive and maturity stages	Genomic regions associated with stay-green identified	Marker-assisted selection for delayed senescence	2015	Japan	Yamanaka et al. (2015)
Gene discovery and functional analysis	*M. truncatula*, *M. sativa* (alfalfa)	MtSGR/MsSGR	SGR1-type gene	Tnt1 insertion, RNAi silencing	Mutant screening, qPCR, GUS assay, RNAi, transgenic analysis	Senescence, nodulation	Stay-green trait, increased crude protein	Genetic improvement of forage quality in alfalfa	2011	United States	Zhou et al. (2011)
Gene functional analysis and mutant characterization	*M. truncatula*	Abscisic acid Insensitive 4 (ABI4), ABI5	Transcription factor (ABA signaling); MtABI4: Chr5g0437371	Loss-of-function mutants (Mtabi4, Mtabi5), double mutants	Mutant analysis, transcriptomics, chlorophyll quantification, seed aging tests	Late seed maturation	Chlorophyll retention, impaired plastid dismantling, reduced seed longevity in mutants	Understanding ABA-mediated regulation of plastid dismantling and seed longevity	2023	France	Zinsmeister et al. (2023)
Comparative genomics and gene identification study	Pea, Arabidopsis, Festuca	Mendel’s I locus (SGR gene)	Stay-green gene (chlorophyll degradation regulator)	RNAi silencing, natural mutations	Comparative genomics, genetic mapping, RNA interference	Leaf and cotyledon senescence	SGR identified as gene underlying Mendel’s I locus; regulates chlorophyll degradation	Fundamental understanding of stay-green genetics across species	2007	United Kingdom	Armstead et al. (2007)
Gene functional and pathway analysis	Pea (*Pisum sativum*), Arabidopsis	SGR, PAO pathway	Stay-green protein (chlorophyll catabolism regulator)	Loss of function and reduced expression (Type C mutant)	Senescence assays, gene expression analysis, protein activity analysis	Senescence stage	SGR acts upstream of PAO; mutation causes chlorophyll retention independent of PAO	Clarifies molecular mechanism of chlorophyll degradation pathway	2008	Switzerland	Aubry et al. (2008)
Mutant physiological and molecular characterization	Soybean	d1d2, cyt-G1 stay-green mutations	Chlorophyll degradation pathway genes	Natural stay-green mutations (d1d2, cyt-G1)	Ultrastructural analysis, pigment analysis, gene expression profiling	Seed development and maturation	Chlorophyll retention resulting from impaired post-translational degradation processes	Insight into chlorophyll retention and degreening processes	1995	United States	Chao et al. (1995)
Gene discovery and molecular mapping study	Common bean (*Phaseolus vulgaris*)	Stay-green (SGR), persistent color (pc)	Stay-green gene family	SNP variation, gene deletion (size polymorphism)	PCR amplification, marker analysis, linkage mapping	Seed maturation and pod stage	pc trait co-segregates with SGR gene; associated with persistent green color	Marker-assisted breeding for pod quality	2010	United States	Davis et al. (2010)
Breeding and genetic analysis study	Cowpea	gt and gc genes (green testa and cotyledon)	Seed color/stay-green related traits	Additive genetic variation	Diallel crosses, genetic analysis (GCA, SCA)	Seed development	Additive gene effects predominant; green seed traits stable without affecting yield traits	Breeding of persistent green seed cowpea lines	2002	Brazil	Freire-Filho et al. (2002)
Genetic mapping and linkage analysis	Soybean	D1, D2, ms5 loci (cotyledon color and male sterility)	Stay-green loci	Mutation (fast neutron-induced ms5), linkage variation	Molecular mapping, cytological analysis	Seed development and reproductive stage	Linkage between stay-green cotyledon color and male sterility locus	Efficient selection of male sterile lines for hybrid seed production	2013	United States	Ott et al. (2013)
Gene functional and molecular characterization	Pea, Rice	SGR (I locus)	Chlorophyll degradation regulator	Natural recessive mutation (I locus)	Gene expression, mutant analysis, molecular characterization	Leaf senescence and seed maturation	SGR regulates chlorophyll degradation; loss of function leads to chlorophyll retention with reduced functionality	Target for manipulating senescence and seed traits	2007	Japan	Sato et al. (2007)
Physiological and transcriptomic analysis	Pea (*Pisum sativum*)	NYC1, PPH, chlorophyll degradation genes	Chlorophyll catabolic pathway enzymes	Natural variation (green vs. yellow seeded cultivars)	Gene expression analysis, physiological assays	Seed maturation	Green seeds due to incomplete chlorophyll degradation; reduced expression of degradation genes	Improving seed quality by targeting chlorophyll degradation	2020	Russia	Smolikova et al. (2020)

Abbreviations and terms in the table reflect different research approaches or study types, crop species, categories of genes or molecular targets, types of genetic mutations or variations, methods or tools, and developmental or stress stages relevant to stay-green research.

* The asterisk (*) in the “Country” column indicates the country of the corresponding author for each publication.

### 3.7 Three field analysis: keywords, journals and countries

**FIGURE 5:**
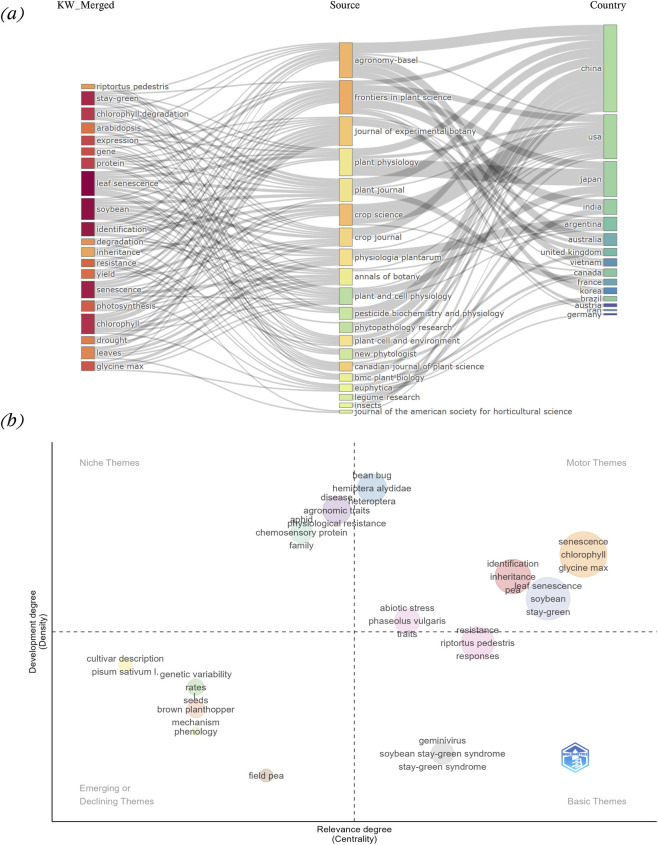
**(a)** Three field plot analysis (Sankey diagram) showing the relationships among keywords, journals, and countries in stay-green research in legumes. KW-Merged: merged keywords (left column), Source: publishing journals (center column), and Country: author affiliated countries (right column). **(b)** Thematic map based on co-occurrence analysis of author keywords and Keywords Plus in stay-green research in legumes. X-axis shows centrality that reflects the measures of importance of a theme to the field and Y-axis shows density that indicates the measures of development of the theme.

The three-field analysis was performed to explore the relationship between three factors including keywords, journals and countries in the stay-green research in legumes (Figure 5a). The left column of the Sankey diagram illustrates dominant research keywords such as chlorophyll degradation, stay-green, leaf senescence, chlorophyll, yield, soybean, and photosynthesis, indicating a strong emphasis on understanding the physiological and molecular mechanisms of senescence and productivity regulation in legumes. These keywords are commonly associated with high-impact journals including the *Journal of Experimental Botany*, *Frontiers in Plant Science*, *Plant Physiology*, and *The Plant Journal*, which form the central part of the plot. On the right, countries such as China, USA, Japan, and India emerge as leading contributors to this field. The linkage of China and USA with a wide range of journals suggests their substantial influence and diverse thematic contributions. The strong connections of the USA to plant science focused journals (such as *Crop Science, Frontiers in Plant Science*) indicate a research orientation toward applied plant improvement and breeding strategies. This tri-visualization emphasizes the global collaboration and specialization of countries in certain sub-domains. For example, senescence related keywords are heavily associated with Chinese authors, potentially reflecting the regional focus on stress adaptation and crop yield stability. This analysis reveals that research on the stay-green in legumes is concentrated in a few countries with advanced plant science infrastructure and disseminated through a mix of fundamental and applied journals. These patterns are critical for identifying potential publication outlets, international collaborators, and research gaps within the thematic space.

### 3.8 Thematic analysis

A thematic map was developed based on co-word analysis to gain insights into the cognitive structure of stay-green research in legumes (Figure 5b). The supporting conceptual structures derived from hierarchical clustering and multivariate analysis are provided in the Supplementary Material (Supplementary Figure S14; Supplementary Figure S15). This diagram categorizes themes into two dimensions: centrality, that reflects the measures of importance of a theme to the field and density, that indicates the measures of development of the theme. In the diagram, the upper-right quadrant comprises motor themes such as senescence, soybean, chlorophyll, stay-green, pea, leaf senescence, inheritance, abiotic stress, and cowpea. These themes are both central and well-developed, serving as the intellectual and methodological core of the field. The basic themes such as geminivirus, riptortus pedestris, soybean stay-green syndrome, and stay-green syndrome are foundational but underdeveloped, suggesting that while these topics are important to the field, they lack cohesive conceptual or methodological development. This presents opportunities for further investigation. The top left quadrant includes niche themes highly developed but relatively isolated within the field. This includes terms like bean bug, hemiptera alydidae, disease, agronomic traits, physiological resistance, and chemosensory protein family. These topics suggest focused research on insect-plant interactions and pest associated physiological responses. In the bottom left quadrant, emerging or declining themes include cultivar description, genetic variability, seeds, brown planthopper, mechanism, phenology, and field pea. These topics show low centrality and low density, suggesting they are either early-stage research fronts or areas where interest is decreasing. For instance, cultivar description, genetic variability, mechanism, and phenology represents topics that remain weakly integrated into the core stay-green research. These themes suggest a new window for trait characterization and phenological studies in relation to stay-green and senescence dynamics in legumes. This thematic analysis provides collective insights into the research landscape of stay-green in legumes, helping researchers and stakeholders to recognize major themes and understand their importance within stay-green research.

## 4 Future research directions

The evolving landscape of stay-green research in legumes, as revealed through bibliometric and thematic analysis, highlights both progress and critical gaps that warrant future investigation. From this, several future research directions can be identified, which are listed below:

1. Although considerable progress has been achieved in identifying stay-green associated genes like SGR, NYC1, and several transcription factors including NAC, WRKY, and bZIP (Brown and Hudson, 2017; Diallo et al., 2024; Dong et al., 2022), most of these studies are still primarily focused on model crops and cereals (Kamal et al., 2019; Sakuraba et al., 2016; Shricharan & Kumar, 2025; Thomas and Ougham, 2014). Future studies should focus on functional validation of these candidate genes specifically in legumes like chickpea, cowpea, and soybean. This can help bridge the gap between molecular characterization and agronomic application.
2. New topics like geminivirus, bean bug, soybean stay-green syndrome, hemiptera alydidae, and *Riptortus pedestris* highlight the connection between plant pathology, entomology, and plant physiology in influencing stay-green traits. With the rise of pathological conditions like soybean stay-green syndrome, there is an urgent need to clearly distinguish beneficial (functional) stay-green phenotypes from genetically non-functional stay-green traits and pathology-associated greenness. The advanced phenotyping platforms, coupled with physiological assays, should be used to differentiate chlorophyll retention due to stress tolerance from that due to viral infections or pest damage.
3. The emergence of keywords such as cultivar description, genetic variability, mechanism, and phenology suggests growing but still limited focus towards trait characterization and developmental timing in relation to stay-green expression. The future research direction points on understanding the genetic and physiological regulation of senescence timing and its interaction with key environmental cues to aid breeders in evaluating legumes varieties that align under variable climatic conditions.
4. The dominance of soybeans in recent studies indicates a species bias, with soybean accounting for most study (30 studies), whereas only limited number of studies have been conducted in other legumes such as pea (8 studies), common bean (7 studies) and cowpea and groundnut with only 4 studies each (Table 5). The future efforts must include diverse legume species such as chickpea, lentil, mung bean, pigeon pea etc. Further, a comparative analysis can help uncover species-specific and conserved mechanisms of stay-green regulation, given the independent domestication histories, genetic diversity and evolutionary divergence among legume crops (Smýkal et al., 2015; Zhang H. et al., 2022).
5. The genetic variation of stay-green traits within global legume germplasm is still insufficiently studied. The screening of core collections can help discover new alleles and structural variants linked to functional stay-green traits. This approach is especially crucial for orphan legumes and underutilized crops in regions like Africa and South Asia.
6. The current co-authorship networks reflect strong China-USA collaboration but reveal weak integration of African and East Asian countries, where legume crops are essential. The promotion of collaborative breeding networks, data sharing, and funding can support triangular cooperation enhancing the local adaptation research and capacity building.
7. Since there are only a few review articles available in this area, a significant gap remains in synthesizing the current knowledge. The future research should prioritize systematic reviews and quantitative meta-analyses that examine trait performance under different abiotic and biotic stresses, genetic regulation, and results from breeding programs.

## 5 Conclusion

This review provides the first consolidated picture of stay-green research in legumes, drawing together bibliometric trends and scientific advances. The field has grown steadily but remains smaller than its cereal counterpart, with a strong base of international collaboration and influential contributions from countries such as China, the United States, and the United Kingdom. The foundational works from the 1990s and early 2000s continue to shape current studies, while more recent efforts have focused on molecular and genomic approaches to understand the regulation of senescence and chlorophyll retention. The benefits of functional stay-green, maintaining photosynthesis during stress, improving harvest index, and stabilizing yield, highlight its promise as a breeding target. At the same time, the uneven focus on soybeans, the limited exploration of other legumes, and the lack of integrative reviews show the need for broader and deeper research. Due to the lack of associated physiological validation where molecular or phenotypic observations were not directly linked to photosynthetic performance or yield outcomes, a subset of studies could not be clearly classified as functional or non-functional stay-green. This shows the need for standardized classification frameworks that integrate molecular, physiological, and agronomic evidence. Future studies should validate stay-green genes across diverse legume species, clearly separate beneficial stay-green from pathological cases, and make use of advanced phenotyping and genomic tools to accelerate trait deployment. The expanding collaborations, particularly in Africa and East Asia where legumes are staple crops, will be crucial for translating research into practical breeding solutions. Therefore, through mapping both the scientific and collaborative landscape, this review points toward the next steps needed to fully realize the potential of the stay-green trait for sustainable legume production.

## Data Availability

The original contributions presented in the study are included in the article/Supplementary Material, further inquiries can be directed to the corresponding author.

## Supplementary material

SUPPLEMENTARY FILE 1:

SUPPLEMENTARY TABLE S1 Search strings used for accessing stay-green research in legumes on Web of Science (WoS) database.

SUPPLEMENTARY TABLE S2 Most frequent keywords in stay-green research on legume crops.

SUPPLEMENTARY TABLE S3 Influential references in stay-green research on legume crops identified through co-citation network analysis.

SUPPLEMENTARY TABLE S4 Core and peripheral journals in stay-green research on legume crops identified using Bradford’s Law.

SUPPLEMENTARY TABLE S5 Network centrality metrics of keywords in the co-word analysis of stay-green research in legume crops.

SUPPLEMENTARY TABLE S6 Keyword co-occurrence network metrics and overlay statistics derived from VOSviewer analysis.

SUPPLEMENTARY TABLE S7 Temporal trends of key research topics in stay-green studies of legume crops.

SUPPLEMENTARY TABLE S8 Network centrality metrics of authors in the collaboration network of stay-green research on legume crops.

SUPPLEMENTARY TABLE S9 Network centrality metrics of authors in the collaboration network of stay-green research on legume crops.

SUPPLEMENTARY TABLE S10 Thematic clusters identified through document coupling analysis of stay-green research in legume crops.

SUPPLEMENTARY TABLE S11 Key terms and centrality metrics of the identification theme derived from thematic map analysis in stay-green research on legume crops.

SUPPLEMENTARY TABLE S12 Author collaboration network metrics and impact indicators derived from VOSviewer analysis.

SUPPLEMENTARY TABLE S13 Institutional collaboration network metrics and scientific impact indicators derived from VOSviewer analysis.

SUPPLEMENTARY TABLE S14 Country level collaboration network metrics and scientific impact indicators derived from VOSviewer analysis.

SUPPLEMENTARY FILE 2: WOS dataset used in the study.

SUPPLEMENTARY FILE 3:

SUPPLEMENTARY FIGURE S1 Most frequent keywords associated with stay-green traits in legume research.

SUPPLEMENTARY FIGURE S2 Word cloud showing dominant research themes in stay-green trait studies of legume crops.

SUPPLEMENTARY FIGURE S3 Temporal evolution of source productivity in stay-green trait research in legumes.

SUPPLEMENTARY FIGURE S4 Reference Publication Year Spectroscopy (RPYS) highlighting the historical knowledge base of stay-green research in legumes.

SUPPLEMENTARY FIGURE S5 Co-citation network of references revealing the intellectual foundation of stay-green research in legume crops.

SUPPLEMENTARY FIGURE S6 Most relevant sources publishing research on stay-green traits in legume crops.

SUPPLEMENTARY FIGURE S7 Identification of core journals in stay-green research on legume crops based on Bradford’s Law.

SUPPLEMENTARY FIGURE S8 Most cited authors in stay-green research within the legume literature.

SUPPLEMENTARY FIGURE S9 Most productive institutional affiliations in stay-green trait research in legume crops.

SUPPLEMENTARY FIGURE S10 Temporal evolution of institutional research output in stay-green trait studies of legumes.

SUPPLEMENTARY FIGURE S11 Temporal trends in country wise scientific production related to stay-green traits in legumes.

SUPPLEMENTARY FIGURE S12 Most cited countries contributing to stay-green trait research in legume crops.

SUPPLEMENTARY FIGURE S13 Keyword co-occurrence network depicting the conceptual structure of stay-green research in legume crops.

SUPPLEMENTARY FIGURE S14 Thematic clusters of stay-green research in legumes based on document coupling analysis.

SUPPLEMENTARY FIGURE S15 Hierarchical clustering dendrogram of keywords revealing thematic relationships in stay-green research in legumes.

SUPPLEMENTARY FIGURE S16 Co-authorship network of authors working on stay-green traits in legumes based on VOSviewer analysis.

SUPPLEMENTARY FIGURE S17 Institutional collaboration network of stay-green traits in legumes based on VOSviewer analysis.

SUPPLEMENTARY FIGURE S18 Country level collaboration network in stay-green traits in legumes based on VOSviewer analysis.

SUPPLEMENTARY FIGURE S19 Temporal trends in keyword frequency reflecting the evolution of stay-green research in legumes.

SUPPLEMENTARY FIGURE S20 Local impact of journals publishing stay-green research in legumes based on H index.

SUPPLEMENTARY FIGURE S21 Local impact of authors in stay-green research on legumes based on H-index.

SUPPLEMENTARY FIGURE S22 Author collaboration network highlighting major clusters in stay-green studies of legumes.

SUPPLEMENTARY FIGURE S23 Global country collaboration map illustrating international research linkages in stay-green trait studies of legumes.

SUPPLEMENTARY FIGURE S24 Conceptual structure map of stay-green research in legumes derived using Multiple Correspondence Analysis (MCA).

SUPPLEMENTARY FIGURE S25 Distribution of author productivity in stay-green research on legumes following Lotka’s Law.

SUPPLEMENTARY FILE 4: Classification of articles based on functional and non-functional categories.

## References

[B1] Abdelrahman M. El-Sayed M. Jogaiah S. Burritt D. J. Tran L. S. P. (2017). The “stay-green” trait and phytohormone signaling networks in plants under heat stress. Plant Cell Rep. 36 (7), 1009–1025. 10.1007/s00299-017-2119-y 28484792

[B2] Abdelwahab S. I. Taha M. M. E. Jerah A. A. Aljahdali I. A. Oraibi B. Alfaifi H. A. (2024). Coffee arabica research (1932–2023): performance, thematic evolution and mapping, global landscape, and emerging trends. Heliyon 10, e36137. 10.1016/j.heliyon.2024.e36137 39224297 PMC11366912

[B3] Al-Jadi M. Myers J. R. Kawai S. Brewer L. J. (2016). Snap-bean germination rates: a comparison of white, persistent color and colored-seeded lines. Annu. Rep. Bean Improv. Coop. 59, 219–220. Available online at: http://arsftfbean.uprm.edu/bic/wp-content/uploads/2018/05/BIC-2016-Annual-Report.pdf .

[B4] Ardie S. W. Nugroho R. B. Dirpan A. Anshori M. F. (2025). Foxtail millet research in supporting climate change resilience efforts: bibliometric analysis and focused literature review. Heliyon 11, e42348. 10.1016/j.heliyon.2025.e42348 39968133 PMC11834093

[B5] Aria M. Cuccurullo C. (2017). Bibliometrix: an R-tool for comprehensive science mapping analysis. J. Informetr. 11 (4), 959–975. 10.1016/j.joi.2017.08.007

[B6] Armstead I. Donnison I. Aubry S. Harper J. Hörtensteiner S. James C. (2007). Cross-species identification of Mendel’s I locus. Science 315, 73. 10.1126/science.1132912 17204643

[B7] Aubry S. Mani J. Hörtensteiner S. (2008). Stay-green protein, defective in Mendel’s green cotyledon mutant, acts independent and upstream of pheophorbide a oxygenase in the chlorophyll catabolic pathway. Plant Mol. Biol. 67 (3), 243–256. 10.1007/s11103-008-9314-8 18301989

[B8] Bachmann A. Fernández-López J. O. S. E. Ginsburg S. Thomas H. Bouwkamp J. C. Solomos T. (1994). Stay-green genotypes of *Phaseolus vulgaris* L.: chloroplast proteins and chlorophyll catabolites during foliar senescence. New Phytol. 126 (4), 593–600. 10.1111/j.1469-8137.1994.tb02953.x

[B9] Badhan S. Ball A. S. Mantri N. (2021). First report of CRISPR/Cas9 mediated DNA-free editing of 4CL and RVE7 genes in chickpea protoplasts. Int. J. Mol. Sci. 22 (1), 1–15. 10.3390/ijms22010396 33401455 PMC7795094

[B10] Balazadeh S. (2014). Stay-green not always stays green. Mol. Plant 7 (8), 1264–1266. 10.1093/mp/ssu076 24996917

[B11] Bawa G. Chen G. Shi J. Ping C. Feng L. Pu T. (2021). Further insights into how low-light signaling delays leaf senescence in soybean under high-temperature. Environ. Exp. Bot. 188, 104516. 10.1016/j.envexpbot.2021.104516

[B12] Bell A. Moreau C. Chinoy C. Spanner R. Dalmais M. Le Signor C. (2015). SGRL can regulate chlorophyll metabolism and contributes to normal plant growth and development in *Pisum sativum* L. Plant Mol. Biol. 89 (6), 539–558. 10.1007/s11103-015-0372-4 26346777 PMC4659853

[B13] Borrell A. Hammer G. van Oosterom E. (2001). Stay-green: a consequence of the balance between supply and demand for nitrogen during grain filling? Ann. Appl. Biol. 138 (1), 91–95. 10.1111/j.1744-7348.2001.tb00088.x

[B14] Borrell A. K. Mullet J. E. George-Jaeggli B. van Oosterom E. J. Hammer G. L. Klein P. E. (2014). Drought adaptation of stay-green sorghum is associated with canopy development, leaf anatomy, root growth, and water uptake. J. Exp. Bot. 65 (21), 6251–6263. 10.1093/jxb/eru232 25381433 PMC4223986

[B15] Brown A. V. Hudson K. A. (2017). Transcriptional profiling of mechanically and genetically sink-limited soybeans. Plant Cell Environ. 40 (10), 2307–2318. 10.1111/pce.13030 28722115

[B16] Canfield M. R. Guiamét J. J. Noodén L. D. (1995). Alteration of soybean seedling development in darkness and light by the stay-green mutation cytG and Gd1d2. Ann. Bot. 75 (2), 143–150. 10.1006/anbo.1995.1005

[B17] Cao J. Liu H. Tan S. Li Z. (2023). Transcription factors-regulated leaf senescence: current knowledge, challenges and approaches. Int. J. Mol. Sci. 24 (11), 9245. 10.3390/ijms24119245 37298196 PMC10253112

[B18] Çelik Ş. (2024). Bibliometric analysis of horticultural crop secondary metabolism. Heliyon 10, e26079. 10.1016/j.heliyon.2024.e26079 38390077 PMC10881373

[B19] Chang H.-X. Tan R. Hartman G. L. Wen Z. Sang H. Domier L. L. (2019). Characterization of soybean stay-green genes in susceptibility to foliar chlorosis of sudden death syndrome. Plant Physiol. 180 (2), 711–717. 10.1104/pp.19.00046 30952683 PMC6548243

[B20] Chao W. S. Liu V. Thomson W. W. Platt K. Walling L. L. (1995). The impact of chlorophyll-retention mutations, d1d2 and cyt-G1, during embryogeny in soybean. Plant Physiol. 107 (1), 253–262. 10.1104/pp.107.1.253 12228359 PMC161196

[B21] Chen J. Zhou H. Yuan X. He Y. Yan Q. Lin Y. (2023). Homolog of pea SGR controls stay-green in faba bean (*Vicia faba* L.). Genes 14 (5), 1030. 10.3390/genes14051030 37239389 PMC10218623

[B22] Choi S. W. Kang J. E. Lee S. K. Ly S. Chung J. I. (2021). Breeding of black soybean with green cotyledon and four recessive alleles for lipoxygenase, Kunitz trypsin inhibitor, lectin, and stachyose. Agronomy 11 (2), 309. 10.3390/agronomy11020309

[B23] Christopher J. T. Christopher M. J. Borrell A. K. Fletcher S. Chenu K. (2016). Stay-green traits to improve wheat adaptation in well-watered and water-limited environments. J. Exp. Bot. 67 (17), 5159–5172. 10.1093/jxb/erw276 27443279 PMC5014159

[B24] Cirak M. Myers J. R. (2021). Cosmetic stay-green trait in snap bean and the event cascade that reduces seed germination and emergence. J. Am. Soc. Hortic. Sci. 146 (5), 329–338. 10.21273/jashs05038-20

[B25] Clifton-Brown J. C. Lewandowski I. Bangerth F. Jones M. B. (2002). Comparative responses to water stress in stay-green, rapid- and slow senescing genotypes of the biomass crop. Miscanthus. New Phytol. 154 (2), 335–345. 10.1046/j.1469-8137.2002.00381.x 33873423

[B26] Danful R. Kassim Y. B. Puozaa D. K. Oteng-Frimpong R. Rasheed M. A. Wireko-Kena A. (2019). Genetics of stay-green trait and its association with leaf spot tolerance and pod yield in groundnut. Int. J. Agron. 2019, 3064026. 10.1155/2019/3064026

[B27] Dasgupta U. Bharath S. Dutta S. (2025). “Abiotic stress and legume production,” in Marker-assisted breeding in legumes for drought tolerance. Editors Nadeem M. A. Baloch F. S. Bantis F. Fiaz S. Aasim M. (Singapore: Springer Nature), 1–21. 10.1007/978-981-96-4112-3_1

[B28] Davis J. Myers J. R. McClean P. Lee R. (2010). Staygreen (sgr), a candidate gene for the persistent color phenotype in common bean. Acta Hortic. 859, 99–102. 10.17660/ActaHortic.2010.859.10

[B29] De Luche H. S. da Silva J. A. G. da Maia L. C. de Oliveira A. C. (2015). Stay-green: a potentiality in plant breeding. Cienc. Rural. 45 (10), 1755–1760. 10.1590/0103-8478cr20140662

[B30] Delmas F. Sankaranarayanan S. Deb S. Widdup E. Bournonville C. Bollier N. (2013). ABI3 controls embryo degreening through Mendel’s I locus. Proc. Natl. Acad. Sci. U.S.A. 110 (40), E3888–E3894. 10.1073/pnas.1308114110 24043799 PMC3791760

[B31] Delprato M. L. Krapp A. R. Carrillo N. (2015). Green light to plant responses to pathogens: the role of chloroplast light-dependent signaling in biotic stress. Photochem. Photobiol. 91 (5), 1004–1011. 10.1111/php.12466 25989185

[B32] Diallo S. Badiane F. A. Diédhiou I. Diouf M. Ngom M. Diouf D. (2024). Development of cowpea (*Vigna unguiculata*) mutant lines for dissecting resilience to drought through physiological and molecular crosstalk analysis. Plant Mol. Biol. Rep. 43 (2), 428–446. 10.1007/s11105-024-01473-2

[B33] Diatta A. A. Abaye O. Battaglia M. L. Leme J. F. D. C. Seleiman M. Babur E. (2024). Mungbean (*Vigna radiata* (L.) Wilczek) and its potential for crop diversification and sustainable food production in Sub-Saharan Africa: a review. Technol. Agron. 4, e031. 10.48130/tia-0024-0030

[B34] Dolferus R. Ji X. Richards R. A. (2011). Abiotic stress and control of grain number in cereals. Plant Sci. 181 (4), 331–341. 10.1016/j.plantsci.2011.05.015 21889038

[B35] Dong S. Pang W. Liu Z. Li H. Zhang K. Cong L. (2022). Transcriptome analysis of leaf senescence regulation under alkaline stress in *Medicago truncatula* . Front. Plant Sci. 13, 881456. 10.3389/fpls.2022.881456 35574123 PMC9096890

[B36] Fang C. Li C. Li W. Wang Z. Zhou Z. Shen Y. (2014). Concerted evolution of D1 and D2 to regulate chlorophyll degradation in soybean. Plant J. 77 (5), 700–712. 10.1111/tpj.12419 24372721

[B37] Farooq M. Gogoi N. Barthakur S. Baroowa B. Bharadwaj N. Alghamdi S. S. (2017). Drought stress in grain legumes during reproduction and grain filling. J. Agron. Crop Sci. 203 (2), 81–102. 10.1111/jac.12169

[B38] Faye J. M. Akata E. A. Sine B. Diatta C. Cisse N. Fonceka D. (2022). Quantitative and population genomics suggest a broad role of stay-green loci in the drought adaptation of sorghum. Plant Genome 15 (1), e20176. 10.1002/tpg2.20176 34817118 PMC12806886

[B39] Fernández-Marín B. García-Plazaola J. I. Hernández A. Esteban R. (2018). “Plant photosynthetic pigments: methods and tricks for correct quantification and identification,” in Advances in plant ecophysiology techniques. Editors Sánchez-Moreiras, A. M. Reigosa M. J. (Cham: Springer International Publishing), 29–50. 10.1007/978-3-319-93233-0_3

[B148] Food and Agriculture Organization of the United Nations (2021). FAOSTAT statistical database. Available online at: https://www.fao.org/faostat/en/#data/QCL .

[B40] Freire-Filho F. R. Chambliss O. L. Hunter A. G. (2002). Crossing potential in the production of persistent green seeds in cowpea using gt and gc genes. Crop Breed. Appl. Biotechnol. 2 (2), 205–212. Available online at: https://cbab.sbmp.org.br/2023/09/26/article-crossing-potential-in-the-production-of-persistent-green-seeds-in-cowpea-using-gtand-gc-genes/ .

[B41] Guiamét J. J. Giannibelli M. C. (1994). Inhibition of the degradation of chloroplast membranes during senescence in nuclear “stay-green” mutants of soybean. Physiol. Plant. 91 (3), 395–402. 10.1111/j.1399-3054.1994.tb02966.x

[B42] Guiamét J. J. Giannibelli M. C. (1996). Nuclear and cytoplasmic “stay-green” mutations of soybean alter the loss of leaf soluble proteins during senescence. Physiol. Plant. 96 (4), 655–661. 10.1111/j.1399-3054.1996.tb00239.x

[B43] Guiamét J. J. Pichersky E. Nooden L. D. (1999). Mass exodus from senescing soybean chloroplasts. Plant Cell Physiol. 40 (9), 986–992. 10.1093/oxfordjournals.pcp.a029632

[B44] Guiamét J. J. Tyystjärvi E. Tyystjärvi T. John I. Kairavuo M. Pichersky E. (2002). Photoinhibition and loss of photosystem II reaction centre proteins during senescence of soybean leaves: enhancement of photoinhibition by the “stay-green” mutation cytG. Physiol. Plant. 115 (3), 468–478. 10.1034/j.1399-3054.2002.1150317.x 12081540

[B45] Gurumurthy S. Sanjay U. N. Amaregouda A. Apoorva A. Kruthika S. Durga G. (2024). Understanding the impact of combined heat and drought stress on the reproductive process of chickpea (*Cicer arietinum* L.). Plant Physiol. Rep. 29 (1), 76–87. 10.1007/s40502-023-00749-1

[B46] Haddad N. I. Salkini A. B. Jagatheeswaran P. Snobar B. A. (1988). “Methods of harvesting pulse crops,” in World crops: cool season food legumes: a global perspective of the problems and prospects for crop improvement in pea, lentil, faba bean and chickpea. Editor Summerfield R. J. (Dordrecht: Springer Netherlands), 341–350. 10.1007/978-94-009-2764-3_31

[B47] Hall A. E. Cisse N. Thiaw S. Elawad H. O. Ehlers J. D. Ismail A. M. (2003). Development of cowpea cultivars and germplasm by the Bean/Cowpea CRSP. Field Crops Res. 82 (2–3), 103–134. 10.1016/s0378-4290(03)00033-9

[B48] Hamza M. Waheed A. Shehzadi I. Tufail U. Hassan A. Hussain T. (2024). Global impact of soybean production: a review. AJBGMB 16 (2), 12–20. 10.9734/AJBGMB/2024/v16i2357

[B49] He H. Li H. Wang Y. Xu Y. Cui X. Zhou X. (2024). Soybean stay-green associated geminivirus: a serious threat to soybean production in China. Virology 602, 110312. 10.1016/j.virol.2024.110312 39586207

[B50] Hörtensteiner S. (2009). Stay-green regulates chlorophyll and chlorophyll-binding protein degradation during senescence. Trends Plant Sci. 14 (3), 155–162. 10.1016/j.tplants.2009.01.002 19237309

[B51] Hu B. Feng X. Xu M. Huang Y. Guo C. Yuan R. (2025). A pentatomomorpha-specific salivary protein activates plant immunity and is critical for insect feeding. Proc. Natl. Acad. Sci. U.S.A. 122 (5), e2425190122. 10.1073/pnas.2425190122 39888915 PMC11804711

[B52] Huang Y. Hu B. Wei Z. Shan S. Guo C. Zhang H. (2023). A secreted salivary effector from *Riptortus pedestris* impairs soybean defense through modulating phytohormone signaling pathways. Insect Sci. 30 (6), 1637–1647. 10.1111/1744-7917.13207 37144452

[B53] Ismail A. M. Hall A. E. Ehlers J. D. (2000). Delayed-leaf-senescence and heat-tolerance traits mainly are independently expressed in cowpea. Crop Sci. 40 (4), 1049–1055. 10.2135/cropsci2000.4041049x

[B54] Jacobs T. B. LaFayette P. R. Schmitz R. J. Parrott W. A. (2015). Targeted genome modifications in soybean with CRISPR/Cas9. BMC Biotechnol. 15 (1), 1–10. 10.1186/s12896-015-0131-2 25879861 PMC4365529

[B55] Jagadish S. V. K. Murty M. V. R. Quick W. P. (2015). Rice responses to rising temperatures: challenges, perspectives and future directions. Plant Cell Environ. 38 (9), 1686–1698. 10.1111/pce.12430 25142172

[B56] Ji X. Dong B. Shiran B. Talbot M. J. Edlington J. E. Hughes T. (2011). Control of abscisic acid catabolism and abscisic acid homeostasis is important for reproductive stage stress tolerance in cereals. Plant Physiol. 156 (2), 647–662. 10.1104/pp.111.176164 21502188 PMC3177265

[B57] Ji J. Zhang C. Sun Z. Wang L. Duanmu D. Fan Q. (2019). Genome editing in cowpea (*Vigna unguiculata*) using CRISPR-Cas9. Int. J. Mol. Sci. 20 (10), 2471. 10.3390/ijms20102471 31109137 PMC6566367

[B58] Jiang G. H. He Y. Q. Xu C. G. Li X. H. Zhang Q. (2004). The genetic basis of stay-green in rice analyzed in a population of doubled haploid lines derived from an indica by japonica cross. Theor. Appl. Genet. 108 (4), 688–698. 10.1007/s00122-003-1465-z 14564397

[B59] Jiao B. Meng Q. Lv W. (2020). Roles of stay-green (SGR) homologs during chlorophyll degradation in green plants. Bot. Stud. 61 (1), 1–12. 10.1186/s40529-020-00302-5 32965575 PMC7511501

[B60] Jo H. Lee J. Y. Cho H. Choi H. J. Son C. K. Bae J. S. (2021). Genetic diversity of soybeans (*Glycine max* (L.) Merr.) with black seed coats and green cotyledons in Korean germplasm. Agronomy 11 (3), 581. 10.3390/agronomy11030581

[B61] Kamal N. M. Alnor Gorafi Y. S. Abdelrahman M. Abdellatef E. Tsujimoto H. (2019). Stay-green trait: a prospective approach for yield potential, and drought and heat stress adaptation in globally important cereals. Int. J. Mol. Sci. 20 (23), 5837. 10.3390/ijms20235837 31757070 PMC6928793

[B62] Kohzuma K. Sato Y. Ito H. Okuzaki A. Watanabe M. Kobayashi H. (2017). The Non-Mendelian green cotyledon gene in soybean encodes a small subunit of photosystem II. Plant Physiol. 173 (4), 2138–2147. 10.1104/pp.16.01589 28235890 PMC5373049

[B63] Krismawati A. Yustisia Y. Arifin Z. Purbiati T. Rachmawati D. Latifah E. (2024). A bibliometric analysis of biopesticides in corn pest management: current trends and future prospects. Heliyon 10, e40196. 10.1016/j.heliyon.2024.e40196 39748967 PMC11693921

[B64] Kumar M. Govindasamy V. Rane J. Singh A. K. Choudhary R. L. Raina S. K. (2017). Canopy temperature depression (CTD) and canopy greenness associated with variation in seed yield of soybean genotypes grown in semi-arid environment. S. Afr. J. Bot. 113, 230–238. 10.1016/j.sajb.2017.08.016

[B65] Kumari S. Khatoon G. Lal S. K. Kumar S. Naskar S. Kumar M. (2024). Nutritional significance of legume crops. Agritecg Today 2, 63–67. Available online at: https://agritechpublication.com/vol-2%2C-issue-5-aug-24-1#b9adeccd-f302-4623-ae8f-07233d3c9a0f .

[B66] Kusaba M. Ito H. Morita R. Iida S. Sato Y. Fujimoto M. (2007). Rice non-yellow coloring1 is involved in light-harvesting complex II and grana degradation during leaf senescence. Plant Cell 19 (4), 1362–1375. 10.1105/tpc.106.042911 17416733 PMC1913755

[B67] Kusaba M. Tanaka A. Tanaka R. (2013). Stay-green plants: what do they tell us about the molecular mechanism of leaf senescence. Photosynth. Res. 117 (1–3), 221–234. 10.1007/s11120-013-9862-x 23771643

[B68] La V. H. Nguyen T. H. A. Ngo X. B. Tran V. D. Khuat H. T. Bui T. T. (2022). At-ORE1 gene induces distinct novel H_2_O_2_-NACs signaling in regulating leaf senescence in soybeans (*Glycine max* L.). Agronomy 12 (9), 2110. 10.3390/agronomy12092110

[B69] Labastida D. Ingvarsson P. K. Rendon-Anaya M. (2023). Dissecting the genetic basis of drought responses in common bean using natural variation. Front. Plant Sci. 14, 1143873. 10.3389/fpls.2023.1143873 37780498 PMC10538545

[B70] Laira M. D. Martins T. S. Almeida R. L. de Moraes-Neto V. F. Pallone J. A. Zambrosi F. C. (2025). Leaf nitrogen spraying improves photosynthesis, seed quality and yield of soybean plants facing post-flowering phosphorus deficiency. J. Soil Sci. Plant Nutr. 25, 1–18. 10.1007/s42729-025-02707-2

[B71] Latif S. Wang L. Khan J. Ali Z. Sehgal S. K. Ali Babar M. (2020). Deciphering the role of stay-green trait to mitigate terminal heat stress in bread wheat. Agronomy 10 (7), 1001. 10.3390/agronomy10071001

[B72] Li K. Zhang X. Guo J. Penn H. Wu T. Li L. (2019). Feeding of *Riptortus pedestris* on soybean plants, the primary cause of soybean stay-green syndrome in the huang-huai-hai river basin. Crop J. 7 (3), 360–367. 10.1016/j.cj.2018.07.008

[B73] Li G. Liu R. Xu R. Varshney R. K. Ding H. Li M. (2023a). Development of an Agrobacterium-mediated CRISPR/Cas9 system in pea (*Pisum sativum* L.). Crop J. 11 (1), 132–139. 10.1016/j.cj.2022.04.011

[B74] Li Y. Chen P. Fu Z. Luo K. Lin P. Gao C. (2023b). Maize–soybean relay cropping increases soybean yield synergistically by extending the post-anthesis leaf stay-green period and accelerating grain filling. Crop J. 11 (6), 1921–1930. 10.1016/j.cj.2023.05.011

[B75] Li Z. Lyu X. Li H. Tu Q. Zhao T. Liu J. (2024). The mechanism of low blue light-induced leaf senescence mediated by GmCRY1s in soybean. Nat. Commun. 15 (1), 798. 10.1038/s41467-024-45086-5 38280892 PMC10821915

[B76] Luquez V. M. Guiamét J. J. (2001). Effects of the “stay-green” genotype GGd1d1d2d2 on leaf gas exchange, dry matter accumulation and seed yield in soybean (*Glycine max* L. Merr.). Ann. Bot. 87 (3), 313–318. 10.1006/anbo.2000.1324

[B77] Luquez V. M. Guiamét J. J. (2002). The stay-green mutations d1 and d2 increase water stress susceptibility in soybeans. J. Exp. Bot. 53 (373), 1421–1428. 10.1093/jexbot/53.373.1421 12021289

[B78] Mahantesh S. Babu R. H. N. Ghanti K. Prasad T. G. Reddy M. K. Raddy P. C. (2018). Over expression of stress responsive *Pennisetum glaucum 47 helicase (PG47)* improves stress tolerance in groundnut (*Arachis hypogaea* L.). Indian J. Agric. Res. 52 (5). 489–496. 10.18805/IJARe.A-5038

[B79] Manjulatha M. Sreevathsa R. Kumar A. M. Sudhakar C. Prasad T. G. Tuteja N. (2014). Overexpression of a pea DNA helicase (PDH45) in peanut (*Arachis hypogaea* L.) confers improvement of cellular level tolerance and productivity under drought stress. Mol. Biotechnol. 56 (2), 111–125. 10.1007/s12033-013-9687-z 23881361

[B80] Manschadi A. M. Christopher J. Devoil P. Hammer G. L. (2006). The role of root architectural traits in adaptation of wheat to water-limited environments. Funct. Plant Biol. 33 (9), 823–837. 10.1071/FP06055 32689293

[B81] Martín-Cabrejas M. A. (2019). “Legumes: an overview,” in Legumes: nutritional quality, processing and potential health benefits (Cambridge: Royal Society of Chemistry). 10.1039/9781788015721-00001

[B82] Martínez-Saldarriaga J. Henao-Rojas J. C. Flórez-Martínez D. H. Cadena-Chamorro E. M. Yepes-Betancur D. P. (2025). Methodological framework for supporting phytochemical bioprospecting research: a case study on carrot (*Daucus carota* L.) crop by-products. Heliyon 11, e41822. 10.1016/j.heliyon.2025.e41822 39916821 PMC11799957

[B83] McCallum J. Timmerman-Vaughan G. Frew T. Russell A. (1997). Biochemical and genetic linkage analysis of green seed color in field pea. J. Am. Soc. Hortic. Sci. 122 (2), 218–225. 10.21273/jashs.122.2.218

[B84] Muchero W. Ehlers J. D. Close T. J. Roberts P. A. (2009). Mapping QTL for drought stress-induced premature senescence and maturity in cowpea [*Vigna unguiculata* (L.) Walp.]. Theor. Appl. Genet. 118 (5), 849–863. 10.1007/s00122-008-0944-7 19130034

[B85] Muchero W. Roberts P. A. Diop N. N. Drabo I. Cisse N. Close T. J. (2013). Genetic architecture of delayed senescence, biomass, and grain yield under drought stress in cowpea. PLoS One 8 (7), e70041. 10.1371/journal.pone.0070041 23936140 PMC3728364

[B86] Munaiz E. D. Martínez S. Kumar A. Caicedo M. Ordás B. (2020). The senescence (stay-green)—An important trait to exploit crop residuals for bioenergy. Energies 13 (4), 790. 10.3390/en13040790

[B87] Myers J. R. Aljadi M. Brewer L. (2018). “The importance of cosmetic stay-green in specialty crops,” Plant Breeding Reviews 42. 219–256. 10.1002/9781119521358.ch6

[B88] Nair C. J. Chimote R. V. P. Chimote V. P. Aher A. R. Shinde S. D. (2025). Transgressive segregation studies in F_2_ progenies under drought condition in soybean (*Glycine max* (L.) Merrill). J. Agric. Res. Technol. 50 (3), 322–329. 10.56228/JART.2025.50312

[B89] Nakano M. Yamada T. Masuda Y. Sato Y. Kobayashi H. Ueda H. (2014). A green-cotyledon/stay-green mutant exemplifies the ancient whole-genome duplications in soybean. Plant Cell Physiol. 55 (10), 1763–1771. 10.1093/pcp/pcu107 25108243

[B90] Naruoka Y. Sherman J. D. Lanning S. P. Blake N. K. Martin J. M. Talbert L. E. (2012). Genetic analysis of green leaf duration in spring wheat. Crop Sci. 52, 99–110. 10.2135/cropsci2011.05.0269

[B91] Nunes-Nesi A. Fernie A. R. Stitt M. (2010). Metabolic and signaling aspects underpinning the regulation of plant carbon nitrogen interactions. Mol. Plant 3 (6), 973–996. 10.1093/mp/ssq049 20926550

[B92] Ott A. Yang Y. Bhattacharyya M. Horner H. T. Palmer R. G. Sandhu D. (2013). Molecular mapping of D1, D2 and ms5 revealed linkage between the cotyledon color locus D2 and the male-sterile locus ms5 in soybean. Plants 2 (3), 441–454. 10.3390/plants2030441 27137386 PMC4844383

[B93] Padilla-Chacón D. Valdivia C. P. García-Esteva A. Cayetano-Marcial M. I. Shibata J. K. (2019). Phenotypic variation and biomass partitioning during post-flowering in two common bean cultivars (*Phaseolus vulgaris* L.) under water restriction. S. Afr. J. Bot. 121, 98–104. 10.1016/j.sajb.2018.10.031

[B94] Page M. J. McKenzie J. E. Bossuyt P. M. Boutron I. Hoffmann T. C. Mulrow C. D. (2021). The PRISMA 2020 statement: an updated guideline for reporting systematic reviews. BMJ 372, n71. 10.1136/bmj.n71 33782057 PMC8005924

[B95] Pandiyan M. Sivakumar P. Krishnaveni A. Sivakumar C. Radhakrishnan V. Vaithiyalingam M. (2021). “Adzuki bean,” in The beans and the peas (Elsevier), 1–15. 10.1016/B978-0-12-821450-3.00006-8

[B96] Peters M. Tarawali S. A. Schultze-Kraft R. (2000). Relative palatability and seasonal agronomic performance of selected pasture legumes for species mixtures in dry-subhumid West Africa. Exp. Agric. 36 (3), 353–368. 10.1017/s0014479700003070

[B97] Rama Reddy N. R. Ragimasalawada M. Sabbavarapu M. M. Nadoor S. Patil J. V. (2014). Detection and validation of stay-green QTL in post-rainy sorghum involving widely adapted cultivar, M35-1 and a popular stay-green genotype B35. BMC Genomics 15, 1–16. 10.1186/1471-2164-15-909 25326366 PMC4219115

[B98] Rolando G. Moraglio S. T. Caratti A. Cordero C. Borreani G. Tavella L. (2025). Quantitative and qualitative damage caused by *Halyomorpha halys* (Hemiptera: pentatomidae) on soybean crop at different growth stages. Crop Prot. 187, 106987. 10.1016/j.cropro.2024.106987

[B99] Ruan Y. L. (2014). Sucrose metabolism: gateway to diverse carbon use and sugar signaling. Annu. Rev. Plant Biol. 65, 33–67. 10.1146/annurev-arplant-050213-040251 24579990

[B100] Sadras V. O. Richards R. A. (2014). Improvement of crop yield in dry environments: benchmarks, levels of organisation and the role of nitrogen. J. Exp. Bot. 65 (8), 1981–1995. 10.1093/jxb/eru061 24638898

[B101] Sakariyahu S. K. Indabo S. S. Aliyu A. Muhammad H. U. Ahmed H. O. Mohammed S. B. (2024). Cowpea landraces in northern Nigeria: overview of seedling drought tolerance. Biologia 79 (2), 381–392. 10.1007/s11756-023-01577-2

[B102] Sakuraba Y. Schelbert S. Park S. Y. Han S. H. Lee B. D. Andres C. B. (2012). STAY-GREEN and chlorophyll catabolic enzymes interact at light-harvesting complex II for chlorophyll detoxification during leaf senescence in *Arabidopsis* . Plant Cell 24 (2), 507–518. 10.1105/tpc.111.089474 22366162 PMC3315229

[B103] Sakuraba Y. Han S.-H. Lee S.-H. Hörtensteiner S. Paek N.-C. (2016). *Arabidopsis* NAC016 promotes chlorophyll breakdown by directly upregulating STAYGREEN1 transcription. Plant Cell Rep. 35 (1), 155–166. 10.1007/s00299-015-1876-8 26441053

[B104] Sato Y. Morita R. Nishimura M. Yamaguchi H. Kusaba M. (2007). Mendel’s green cotyledon gene encodes a positive regulator of the chlorophyll-degrading pathway. Proc. Natl. Acad. Sci. U.S.A. 104 (35), 14169–14174. 10.1073/pnas.0705521104 17709752 PMC1955798

[B105] Schmit R. Melo R. C. D. Trevisani N. Guidolin A. F. Coimbra J. L. M. (2019). Screening and agronomic benefits of the stay-green trait in common bean genotypes. Biosci. J. 35, 869–877. 10.14393/bj-v35n3a2019-42022

[B106] Shan S. Huang Y. Guo C. Hu B. Zhang H. Li Y. (2023). A salivary secretory protein from *Riptortus pedestris* facilitates pest infestation and soybean staygreen syndrome. Mol. Plant Pathol. 24 (6), 560–569. 10.1111/mpp.13323 36916884 PMC10189764

[B107] Shi S. Miao H. Du X. Gu J. Xiao K. (2016). GmSGR1, a stay-green gene in soybean (*Glycine max* L.), plays an important role in regulating early leaf-yellowing phenotype and plant productivity under nitrogen deprivation. Acta Physiol. Plant. 38 (4), 101. 10.1007/s11738-016-2105-y

[B108] Shricharan S. Kumar P. (2025). Stay green to stay alive: hormonal regulation of stay green trait under heat stress in crop plants. Plant Physiol. Rep. 30 (3), 480–490. 10.1007/s40502-025-00887-8

[B109] Sivasakthi K. Marques E. Kalungwana N. Carrasquilla-Garcia N. Chang P. L. Bergmann E. M. (2019). Functional dissection of the chickpea (*Cicer arietinum* L.) stay-green phenotype associated with molecular variation at an ortholog of Mendel’s I gene for cotyledon color: implications for crop production and carotenoid biofortification. Int. J. Mol. Sci. 20 (22), 5562. 10.3390/ijms20225562 31703441 PMC6888616

[B110] Smolikova G. Dolgikh E. Vikhnina M. Frolov A. Medvedev S. (2017). Genetic and hormonal regulation of chlorophyll degradation during maturation of seeds with green embryos. Int. J. Mol. Sci. 18 (9), 1993. 10.3390/ijms18091993 28926960 PMC5618642

[B111] Smolikova G. Shiroglazova O. Vinogradova G. Leppyanen I. Dinastiya E. Yakovleva O. (2020). Comparative analysis of plastid conversion, photochemical activity and chlorophyll degradation in developing embryos of green-seeded and yellow-seeded pea (*Pisum sativum*) cultivars. Funct. Plant Biol. 47 (5), 409–424. 10.1071/FP19270 32209205

[B112] Smýkal P. Coyne C. J. Ambrose M. J. Maxted N. Schaefer H. Blair M. W. (2015). Legume crops phylogeny and genetic diversity for science and breeding. Crit. Rev. Plant Sci. 34 (1–3), 43–104. 10.1080/07352689.2014.897904

[B113] Stoddard F. L. (2017). “Grain legumes: an overview,” in Legumes in cropping systems, 70–87. 10.1079/9781780644981.0070

[B114] Ta C. T. Weiland R. T. (1992). Nitrogen partitioning in maize during ear development. Crop Sci. 32 (2), 443–451. 10.2135/cropsci1992.0011183x003200020032x

[B115] Teixeira R. N. Ligterink W. França-Neto J. D. B. Hilhorst H. W. da Silva E. A. (2016). Gene expression profiling of the green seed problem in soybean. BMC Plant Biol. 16 (1), 37. 10.1186/s12870-016-0729-0 26829931 PMC4736698

[B116] Thomas H. Howarth C. J. (2000). Five ways to stay green. J. Exp. Bot. 51 (Suppl. 1), 329–337. 10.1093/jexbot/51.suppl_1.329 10938840

[B117] Thomas H. Ougham H. (2014). The stay-green trait. J. Exp. Bot. 65 (14), 3889–3900. 10.1093/jxb/eru037 24600017

[B118] Thomas H. Smart C. M. (1993). Crops that stay green. Ann. Appl. Biol. 123 (1), 193–219. 10.1111/j.1744-7348.1993.tb04086.x

[B119] Thomas H. Ougham H. Canter P. Donnison I. (2002). What stay-green mutants tell us about nitrogen remobilization in leaf senescence. J. Exp. Bot. 53 (370), 801–808. 10.1093/jexbot/53.370.801 11912223

[B120] Tian F. X. Gong J. F. Wang G. P. Wang G. K. Fan Z. Y. Wang W. (2012). Improved drought resistance in a wheat stay-green mutant tasg1 under field conditions. Biol. Plant. 56 (3), 509–515. 10.1007/s10535-012-0049-7

[B121] Tokumitsu Y. Kozu T. Yamatani H. Ito T. Nakano H. Hase A. (2022). Functional divergence of G and its homologous genes for green pigmentation in soybean seeds. Front. Plant Sci. 12, 796981. 10.3389/fpls.2021.796981 35069653 PMC8766641

[B122] Ubayasena L. Vijayan P. Bett K. E. Gray G. R. Küster H. Warkentin T. D. (2013). Gene expression profiles of seed coats and biochemical properties of seed coats and cotyledons of two field pea (*Pisum sativum*) cultivars contrasting in green cotyledon bleaching resistance. Euphytica 193 (1), 49–65. 10.1007/s10681-013-0914-2

[B123] Van Eck N. Waltman L. (2010). Software survey: vosviewer, a computer program for bibliometric mapping. Scientometrics 84 (2), 523–538. 10.1007/s11192-009-0146-3 20585380 PMC2883932

[B124] Vaz Patto M. C. Amarowicz R. Aryee A. N. A. Boye J. I. Chung H.-J. Martín-Cabrejas M. A. (2015). Achievements and challenges in improving the nutritional quality of food legumes. Crit. Rev. Plant Sci. 34 (1–3), 105–143. 10.1080/07352689.2014.897907

[B125] Venkatesh B. Vennapusa A. R. Kumar N. J. Jayamma N. Reddy B. M. Johnson A. M. (2022). Co-expression of stress-responsive regulatory genes, MuNAC4, MuWRKY3 and MuMYB96 associated with resistant traits improves drought adaptation in transgenic groundnut (*Arachis hypogaea* L.) plants. Front. Plant Sci. 13, 1055851. 10.3389/fpls.2022.1055851 36466254 PMC9709484

[B126] Wang M. Li W. Fang C. Xu F. Liu Y. Wang Z. (2018). Parallel selection on a dormancy gene during domestication of crops from multiple families. Nat. Genet. 50 (10), 1435–1441. 10.1038/s41588-018-0229-2 30250128

[B127] Wang Y. Tan J. Wu Z. Vandenlangenberg K. Wehner T. C. Wen C. (2019). STAYGREEN, STAY HEALTHY: a loss-of-susceptibility mutation in the STAYGREEN gene provides durable, broad-spectrum disease resistances for over 50 years of US cucumber production. New Phytol. 221 (1), 415–430. 10.1111/nph.15353 30022503

[B128] Wang T. Wang S. Wang Y. Li J. Yan F. Liu Y. (2020a). Jasmonic acid-induced inhibition of root growth and leaf senescence is reduced by GmbHLH3, a soybean bHLH transcription factor. Can. J. Plant Sci. 100 (5), 477–487. 10.1139/cjps-2019-0250

[B129] Wang C. Gao L. Li R. Wang Y. Liu Y. Zhang X. (2020b). High-throughput sequencing reveals the molecular mechanisms determining the stay-green characteristic in soybeans. J. Biosci. 45 (1), 1–14. 10.1007/s12038-020-00074-x 32975230

[B130] Wang P. Hou S. Y. Wen H. W. Wang Q. Z. Li G. Q. (2021a). Chlorophyll retention caused by STAY-GREEN (SGR) gene mutation enhances photosynthetic efficiency and yield in soybean hybrid Z1. Photosynthetica 59 (1), 37–48. 10.32615/ps.2020.076

[B131] Wang P. Hou S. Wen H. Wang Q. Li G. (2021b). The stay-green mutation contributes to enhanced antioxidative competence and delays leaf senescence in soybean hybrid Z1. Int. J. Agric. Biol. 25 (2), 361–372. 10.17957/IJAB/15.1676

[B132] Wang X. Wang M. Wang L. Feng H. He X. Chang S. (2022). Whole-plant microbiome profiling reveals a novel geminivirus associated with soybean stay-green disease. Plant Biotechnol. J. 20 (11), 2159–2173. 10.1111/pbi.13896 35869670 PMC9616524

[B133] Wang M. Hong L. Zhang W. Xu Y. Yuan F. Zhou C. (2025a). Functional characterization of chlorophyll b reductase NON-YELLOW COLORING 1 in *Medicago truncatula* . Plant Sci. 350, 112307. 10.1016/j.plantsci.2024.112307 39461562

[B134] Wang G. Hu B. Yao X. Wei Z. Chen J. Sun Z. (2025b). A stinkbug salivary protein is indispensable for insect feeding and activates plant immunity. Plant Cell Environ. 48 (3), 2329–2342. 10.1111/pce.15308 39593264

[B135] Wang D. Wang Y. Sun R. Yang Y. Zhao W. Yu G. (2025c). Transcriptomics and physiological analyses of soybean stay-green syndrome. Agronomy 15 (1), 82. 10.3390/agronomy15010082

[B136] Wei Z. Guo W. Jiang S. Yan D. Shi Y. Wu B. (2023). Transcriptional profiling reveals a critical role of GmFT2a in soybean staygreen syndrome caused by the pest *Riptortus pedestris* . New Phytol. 237 (5), 1876–1890. 10.1111/nph.18628 36404128

[B137] Wolabu T. W. Cong L. Park J. J. Bao Q. Chen M. Sun J. (2020). Development of a highly efficient multiplex genome editing system in outcrossing tetraploid alfalfa (*Medicago sativa*). Front. Plant Sci. 11, 1063. 10.3389/fpls.2020.01063 32765553 PMC7380066

[B138] Yamanaka N. Lemos N. G. Jara R. C. Hossain M. M. Suenaga K. Yamaoka Y. (2015). Prevention of leaf yellowing in Asian soybean rust infected plants is associated with green cotyledon color and the infection index. Euphytica 205 (2), 475–482. 10.1007/s10681-015-1414-3

[B139] Yang J. Carena M. J. Uphaus J. (2010). Area under the dry down curve (AUDDC): a method to evaluate rate of dry down in maize. Crop Sci. 50, 2347–2354. 10.2135/cropsci2010.02.0098

[B140] Zhang X. Wang M. Wu T. Wu C. Jiang B. Guo C. (2016). Physiological and molecular studies of staygreen caused by pod removal and seed injury in soybean. Crop J. 4 (6), 435–443. 10.1016/j.cj.2016.04.002

[B141] Zhang P. Du H. Wang J. Pu Y. Yang C. Yan R. (2020). Multiplex CRISPR/Cas9-mediated metabolic engineering increases soya bean isoflavone content and resistance to soya bean mosaic virus. Plant Biotechnol. J. 18 (6), 1384–1395. 10.1111/pbi.13302 31769589 PMC7206993

[B142] Zhang M. Liu S. Wang Z. Yuan Y. Zhang Z. Liang Q. (2022a). Progress in soybean functional genomics over the past decade. Plant Biotechnol. J. 20 (2), 256–282. 10.1111/pbi.13682 34388296 PMC8753368

[B143] Zhang H. Mascher M. Abbo S. Jayakodi M. (2022b). Advancing grain legumes domestication and evolution studies with genomics. Plant Cell Physiol. 63 (11), 1540–1553. 10.1093/pcp/pcac062 35534441 PMC9680859

[B144] Zhao W. Zhao H. Wang H. He Y. (2022). Research progress on the relationship between leaf senescence and quality, yield and stress resistance in horticultural plants. Front. Plant Sci. 13, 1044500. 10.3389/fpls.2022.1044500 36352873 PMC9638160

[B145] Zheng Y. P. (2024). Global characteristics and trends of researches on watermelon: based on bibliometric and visualized analysis. Heliyon 10, e26824. 10.1016/j.heliyon.2024.e26824 38434322 PMC10907791

[B146] Zhou C. Han L. Pislariu C. Nakashima J. Fu C. Jiang Q. (2011). From model to crop: functional analysis of a stay-green gene in the model legume *Medicago truncatula* and effective use of the gene for alfalfa improvement. Plant Physiol. 157 (3), 1483–1496. 10.1104/pp.111.185140 21957014 PMC3252161

[B147] Zinsmeister J. Lalanne D. Ly Vu B. Schoefs B. Marchand J. Dang T. T. (2023). ABSCISIC ACID INSENSITIVE 4 coordinates eoplast formation to ensure acquisition of seed longevity during maturation in *Medicago truncatula* . Plant J. 113 (5), 934–953. 10.1111/tpj.16091 36582182

