# Supplementary material for: Development and research trends of stay-green biology in legumes: a bibliometric and visual analysis over three decades
Source: Front Genet. 2026 Apr 24;17:1800699. doi: 10.3389/fgene.2026.1800699 (PMC13152256; doi:10.3389/fgene.2026.1800699)
Supplement: Supplementary file 4 [file Supplementaryfile3.pdf]

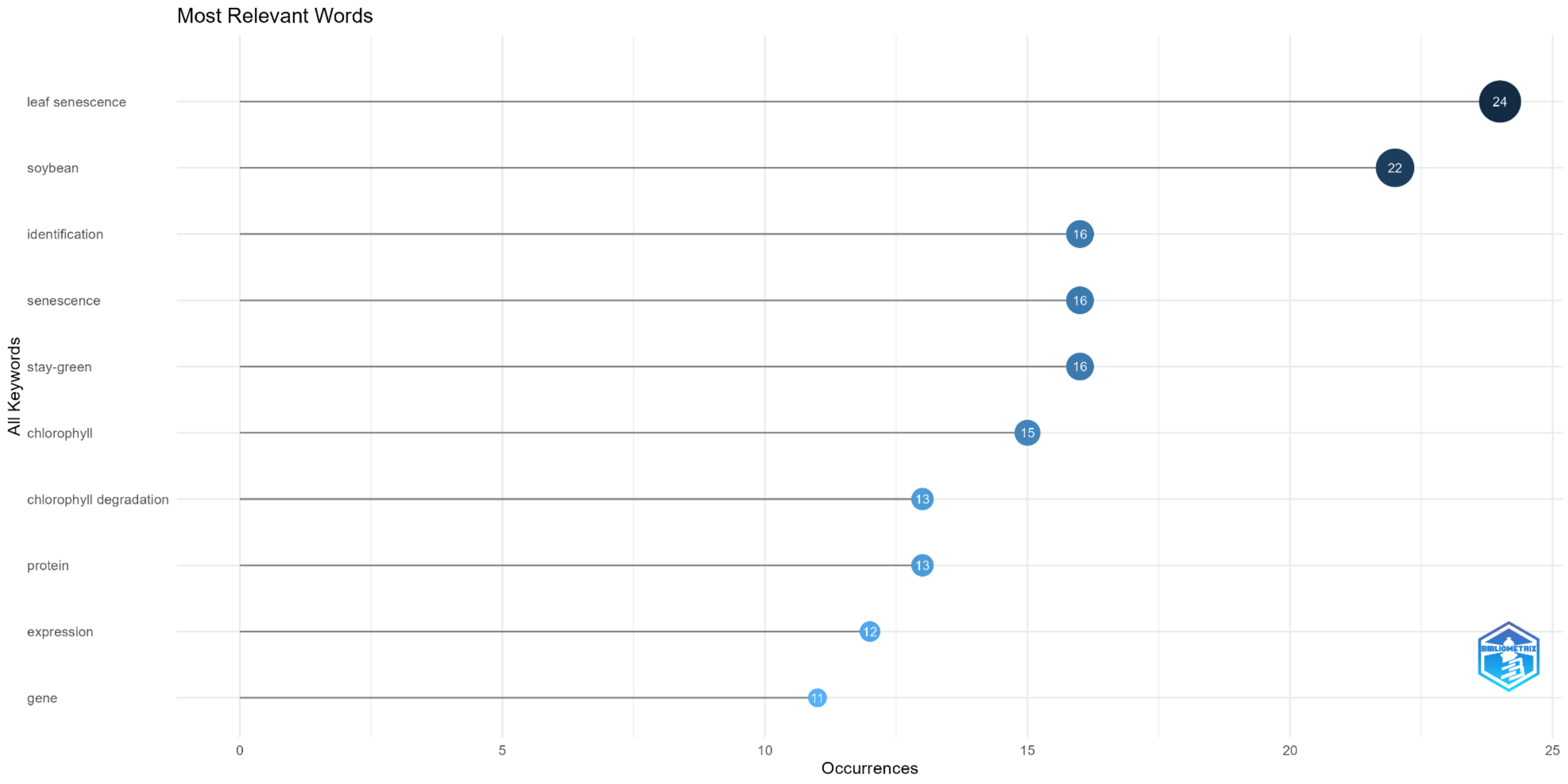

Figure S1: Most frequent keywords associated with stay-green traits in legume research.

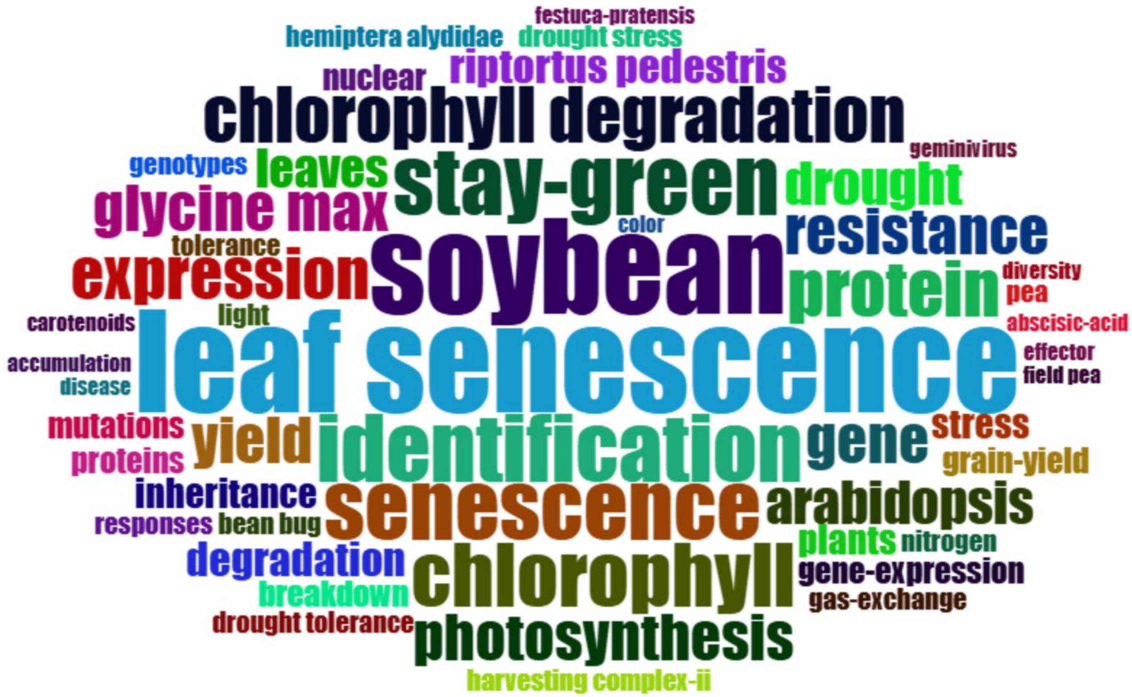

Figure S2: Word cloud showing dominant research themes in stay-green trait studies of legume crops.

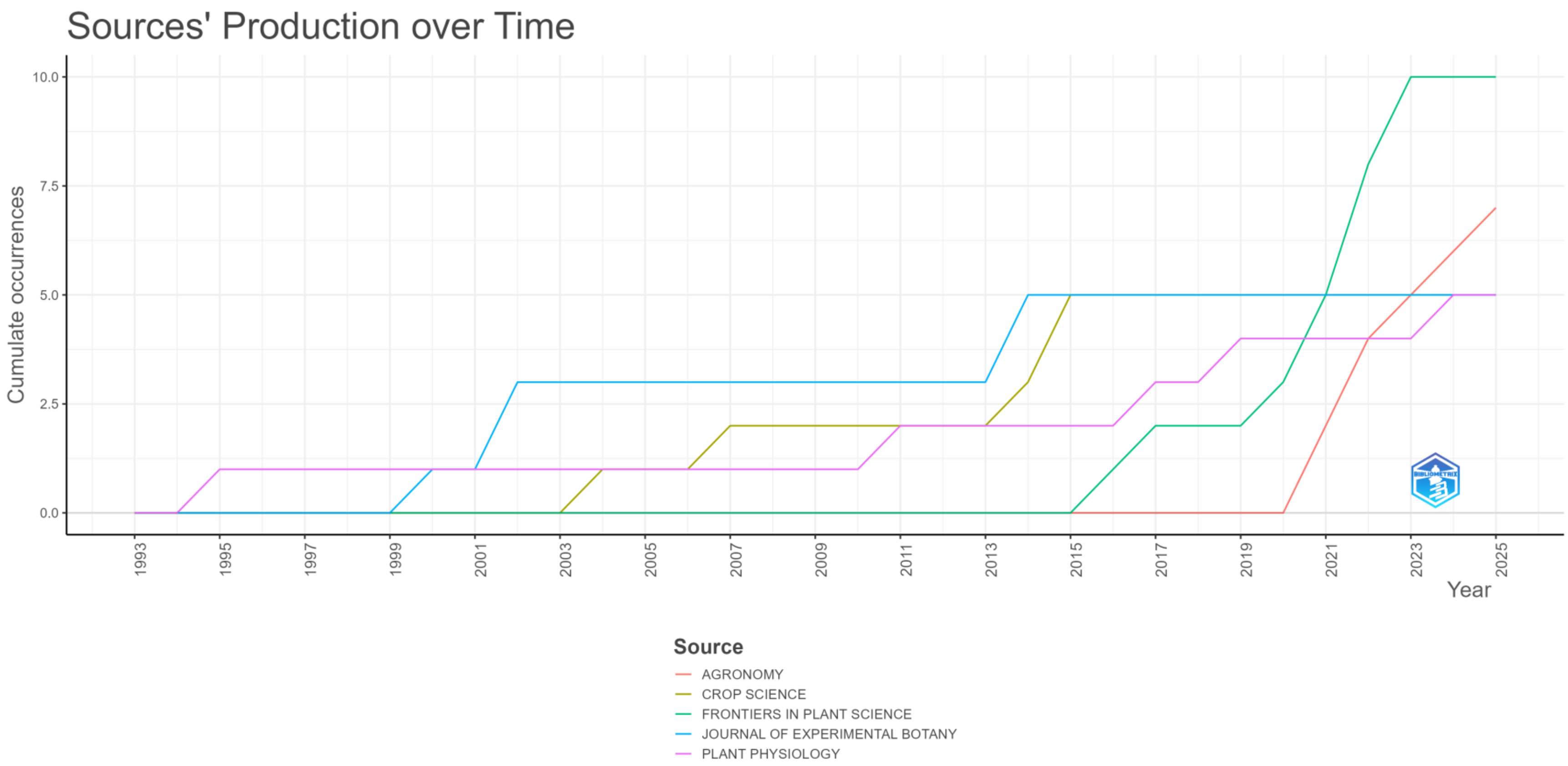

Figure S3: Temporal evolution of source productivity in stay-green trait research in legumes.

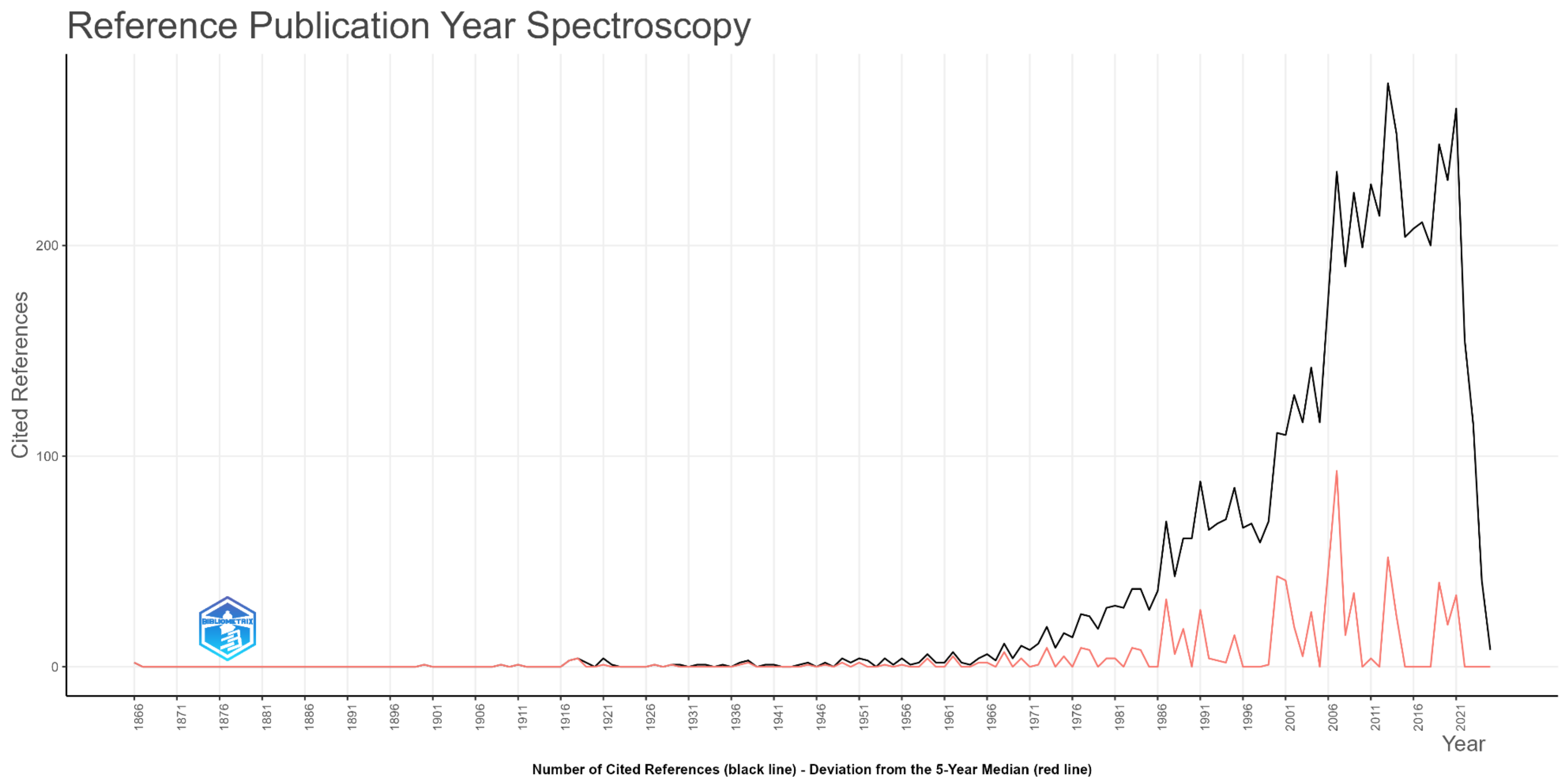

Figure S4: Reference Publication Year Spectroscopy (RPYS) highlighting the historical knowledge base of stay-green research in legumes.

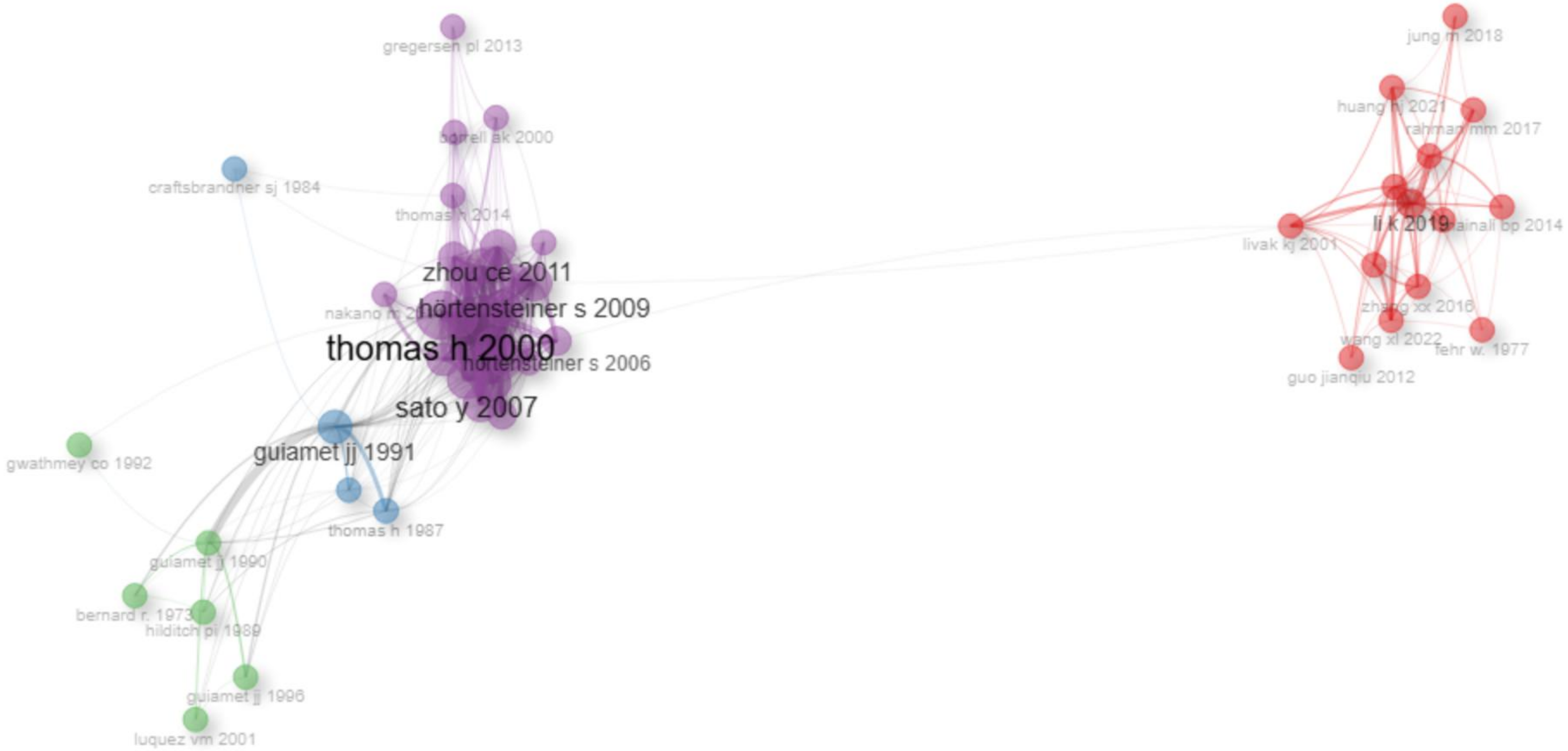

Figure S5: Co-citation network of references revealing the intellectual foundation of stay-green research in legume crops.

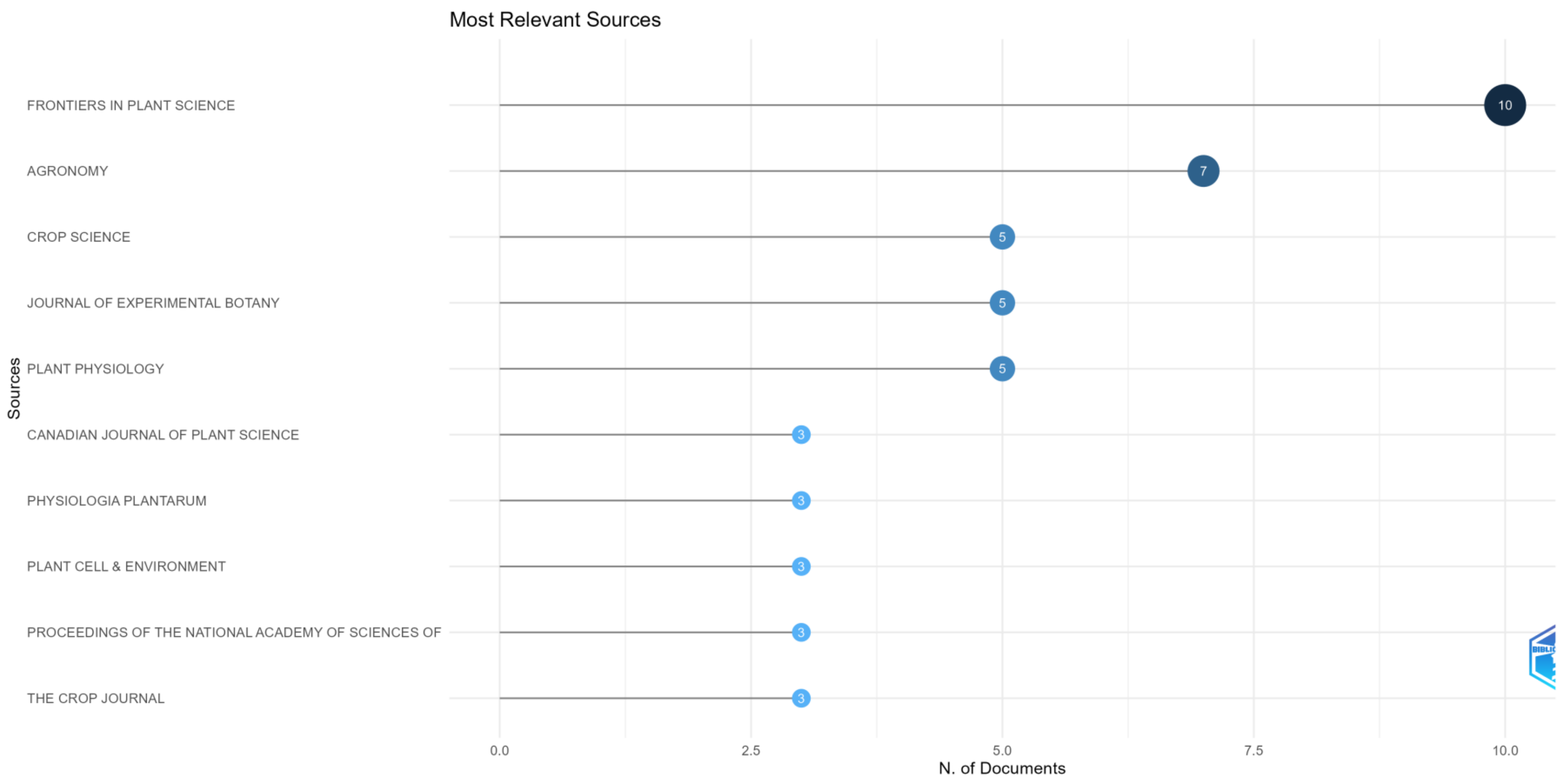

Figure S6: Most relevant sources publishing research on stay-green traits in legume crops.

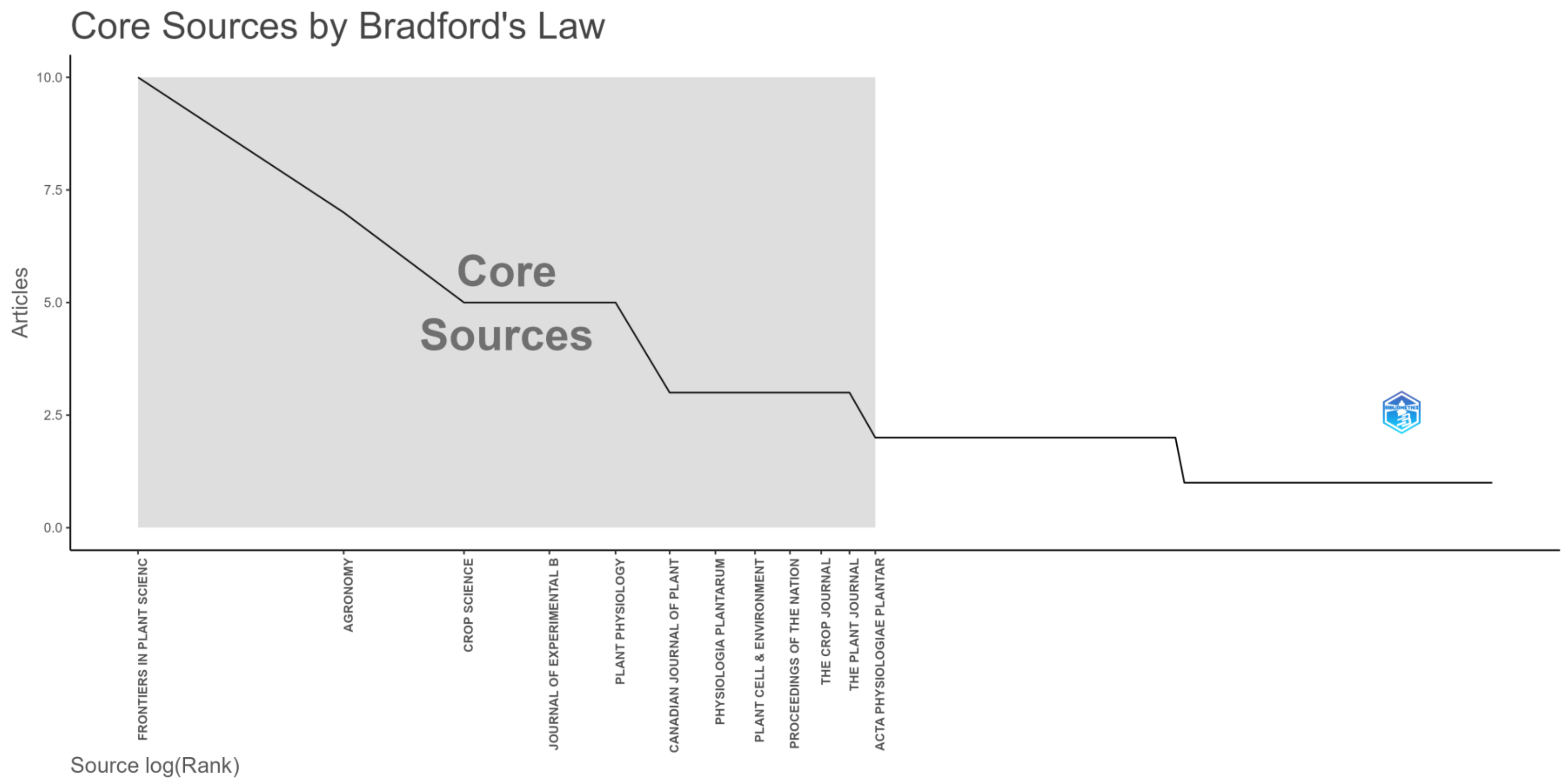

Figure S7: Identification of core journals in stay-green research on legume crops based on Bradford’s Law.

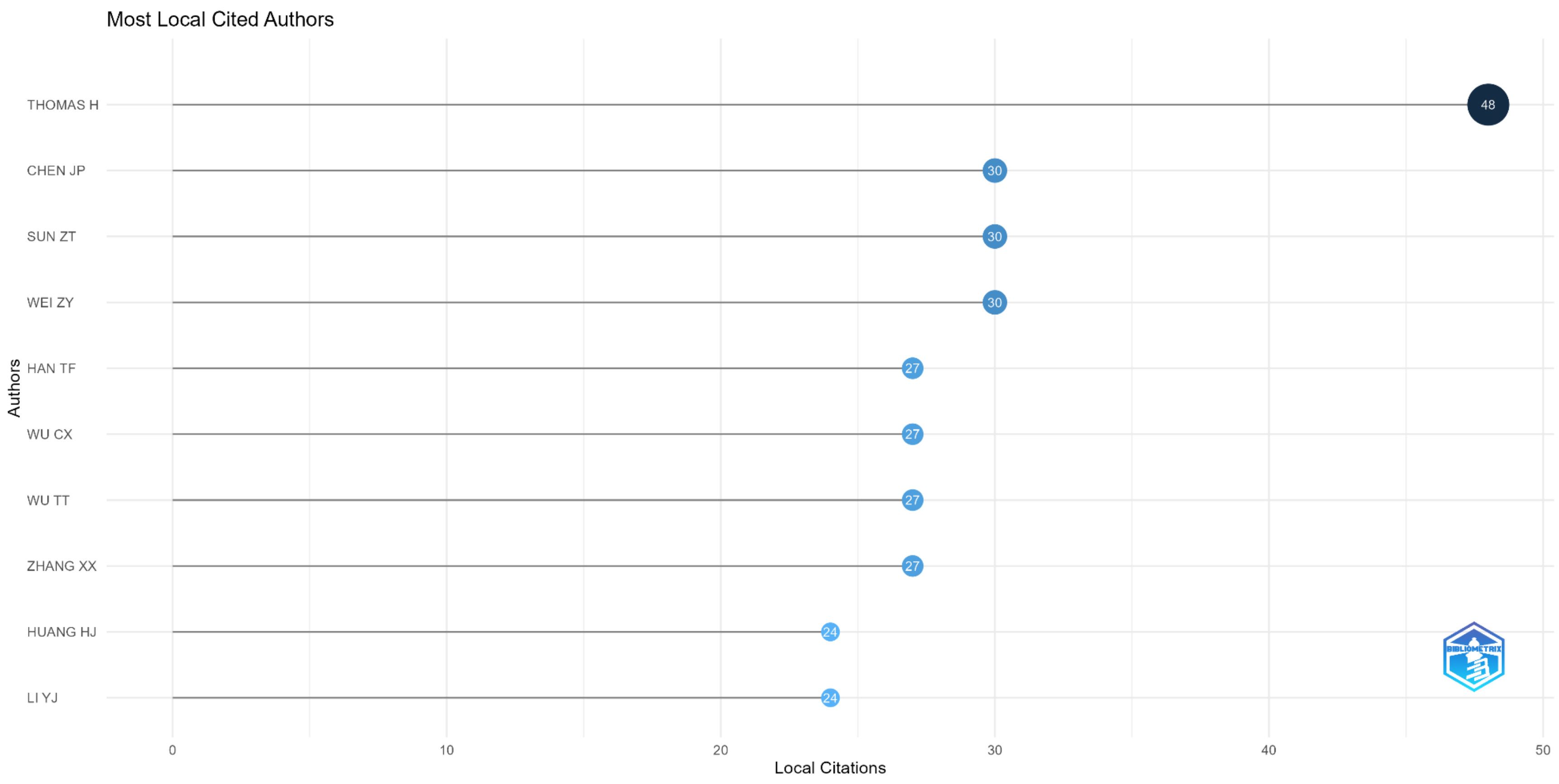

Figure S8: Most cited authors in stay green research within the legume literature.

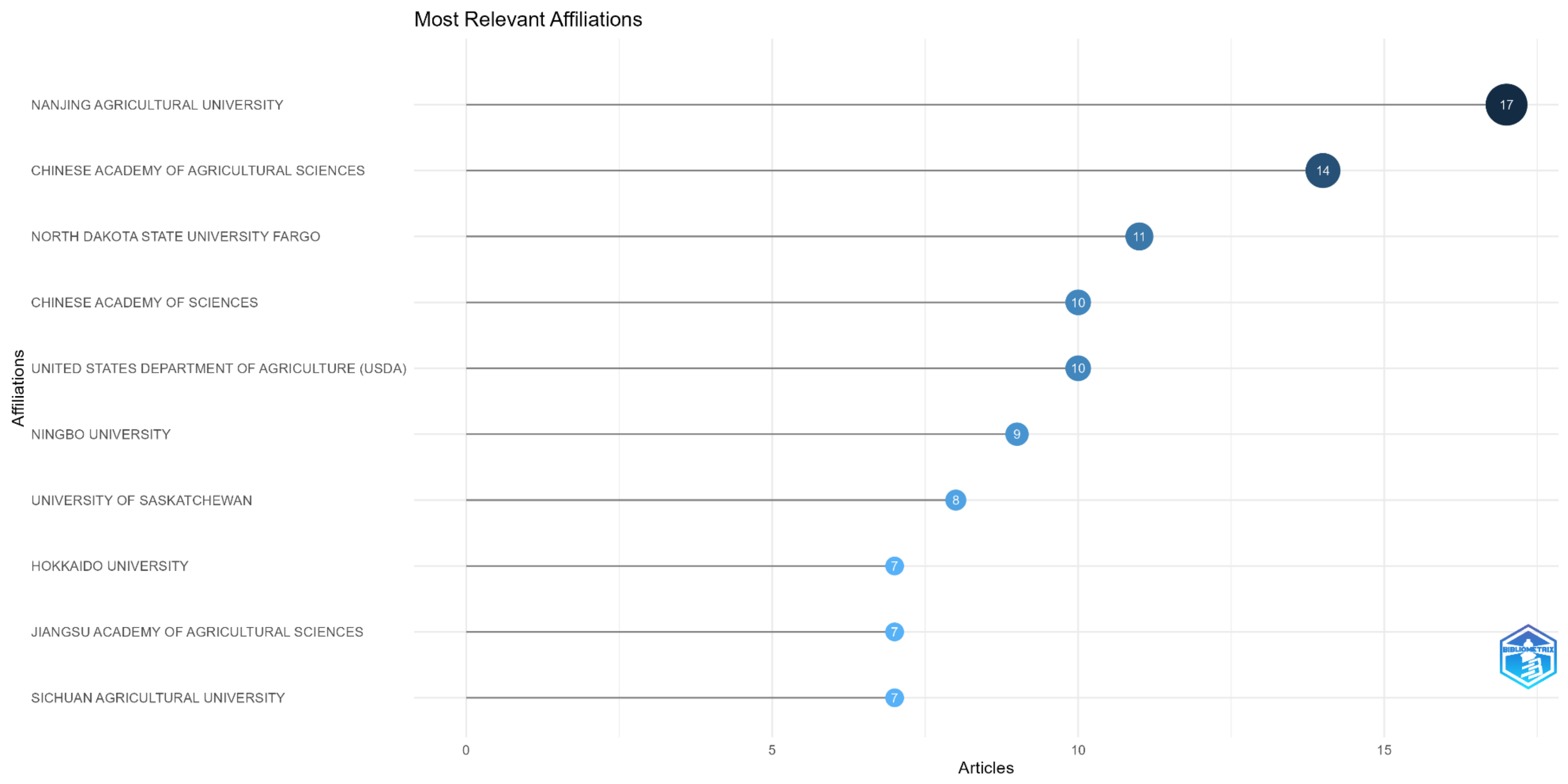

Figure S9: Most productive institutional affiliations in stay-green trait research in legume crops.

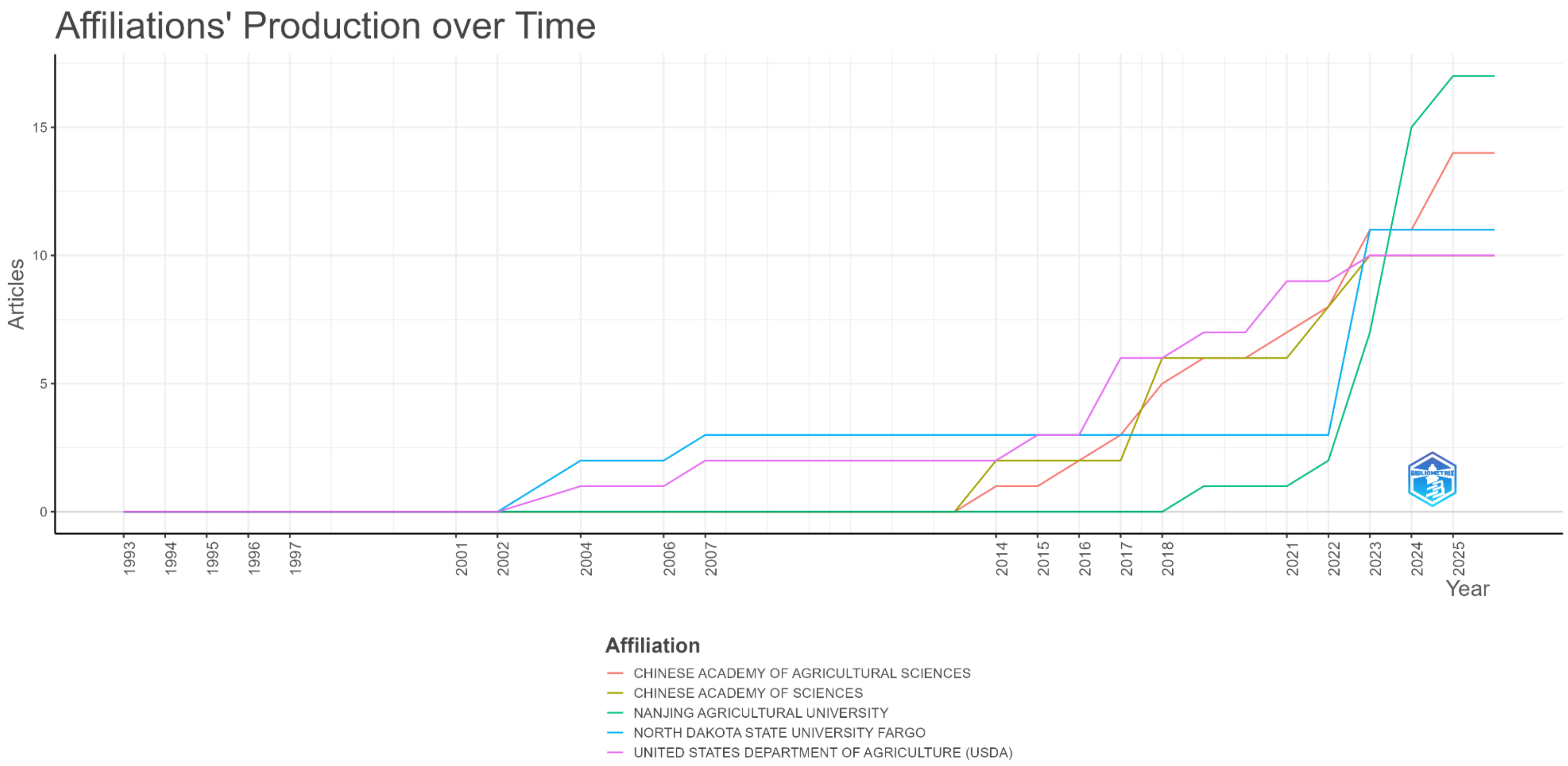

Figure S10: Temporal evolution of institutional research output in stay-green trait studies of legumes

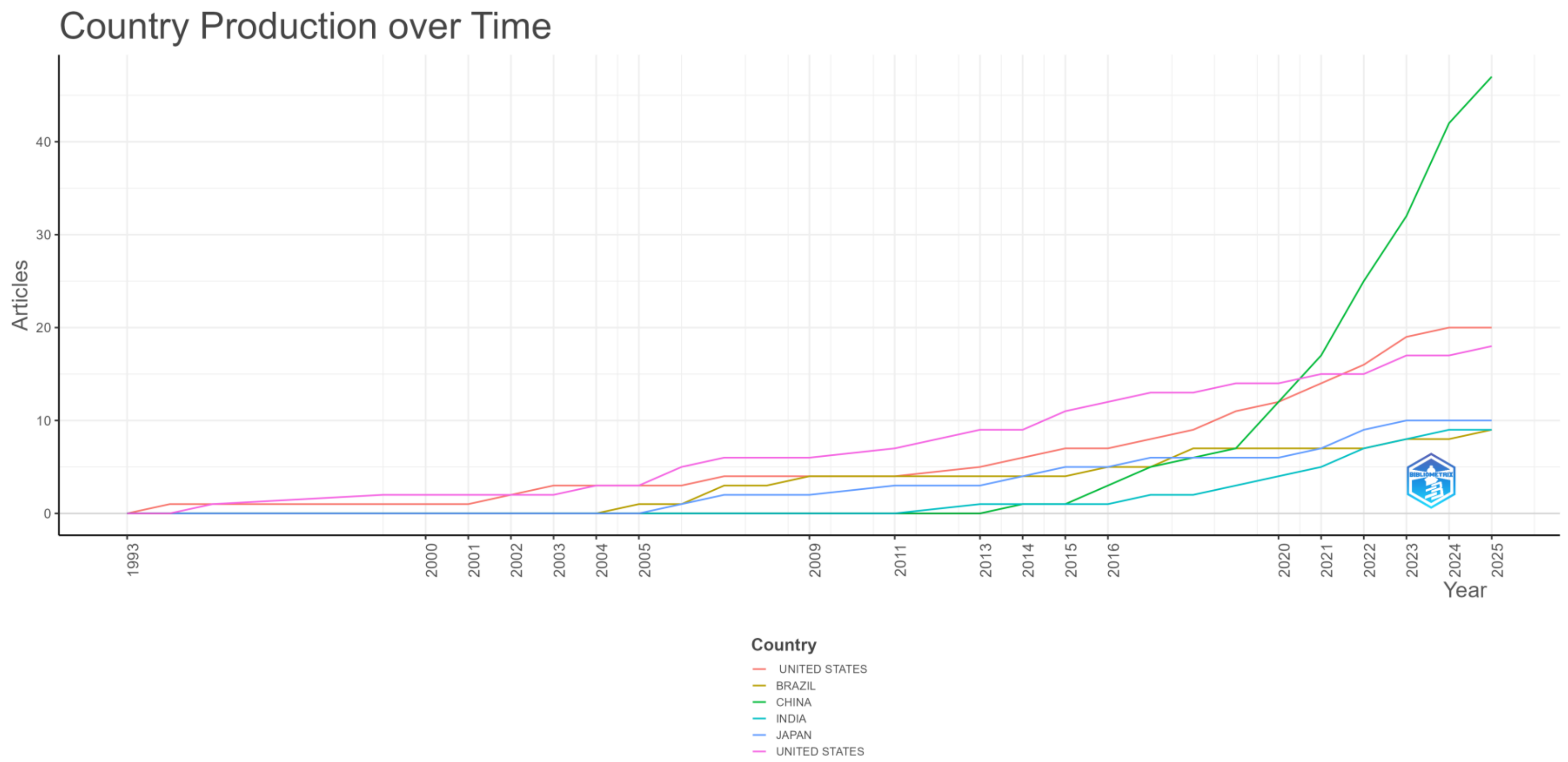

Figure S11: Temporal trends in country wise scientific production related to stay-green traits in legumes.

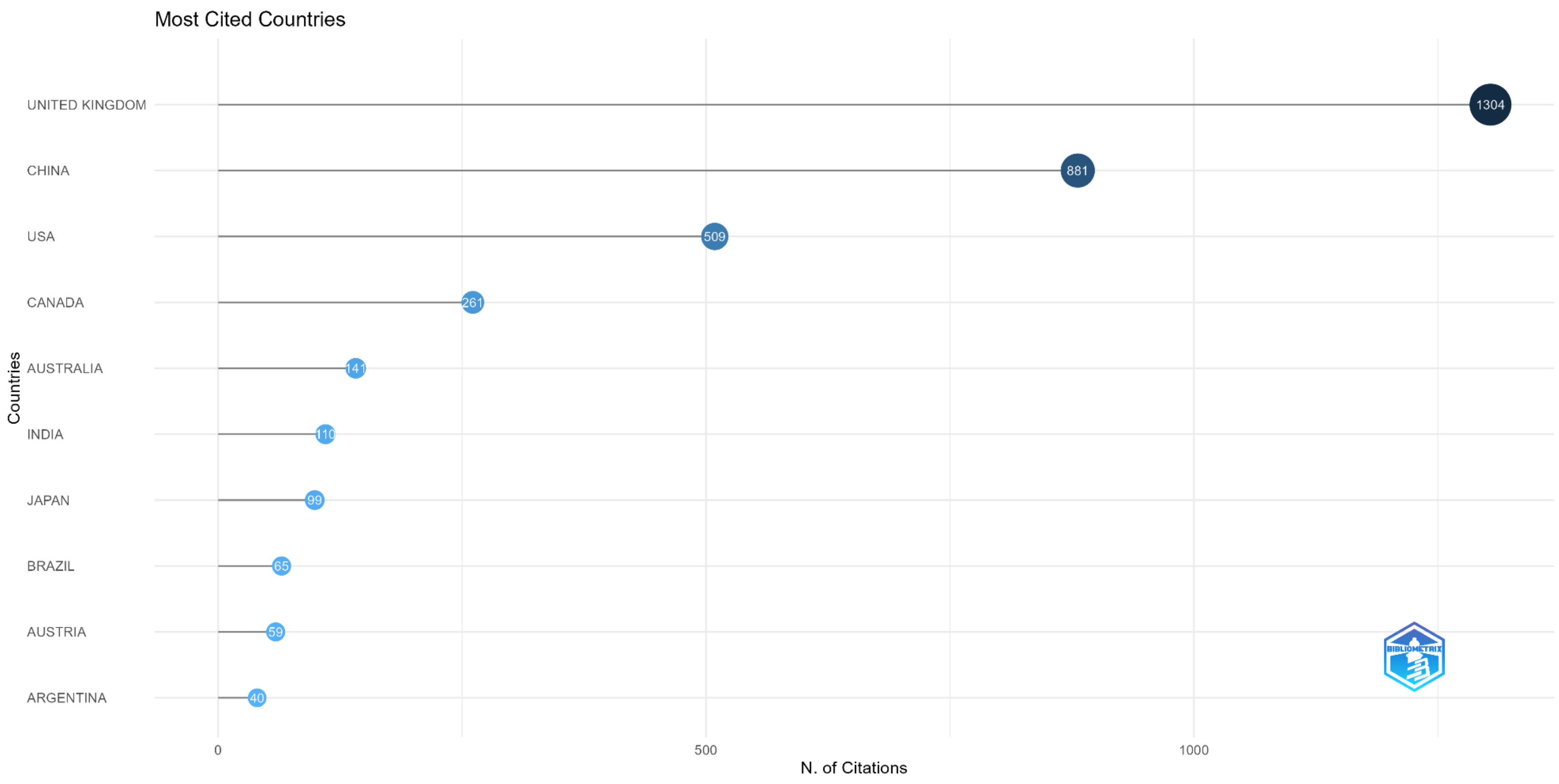

Figure S12: Most cited countries contributing to stay green trait research in legume crops.

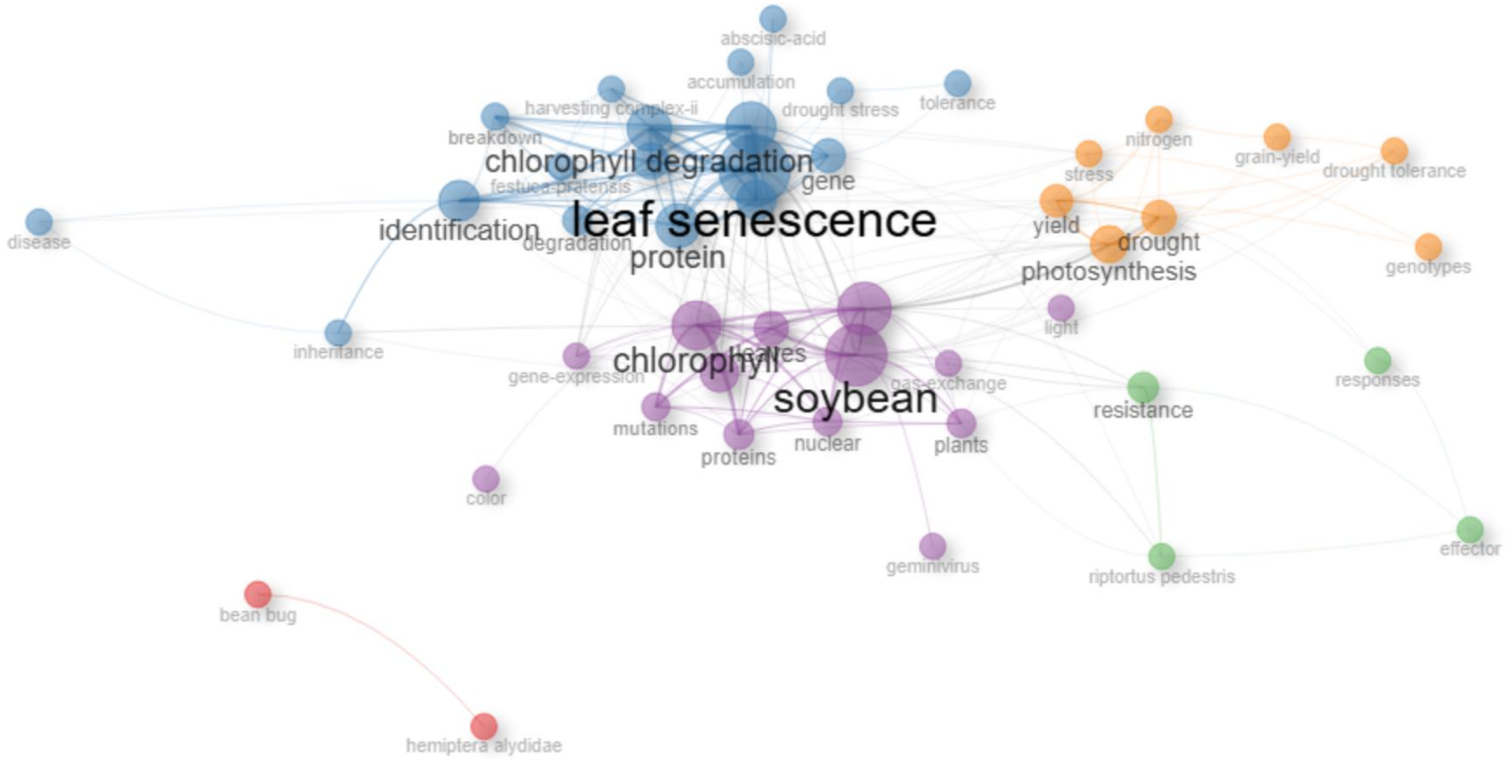

Figure S13: Keyword co-occurrence network depicting the conceptual structure of stay-green research in legume crops.

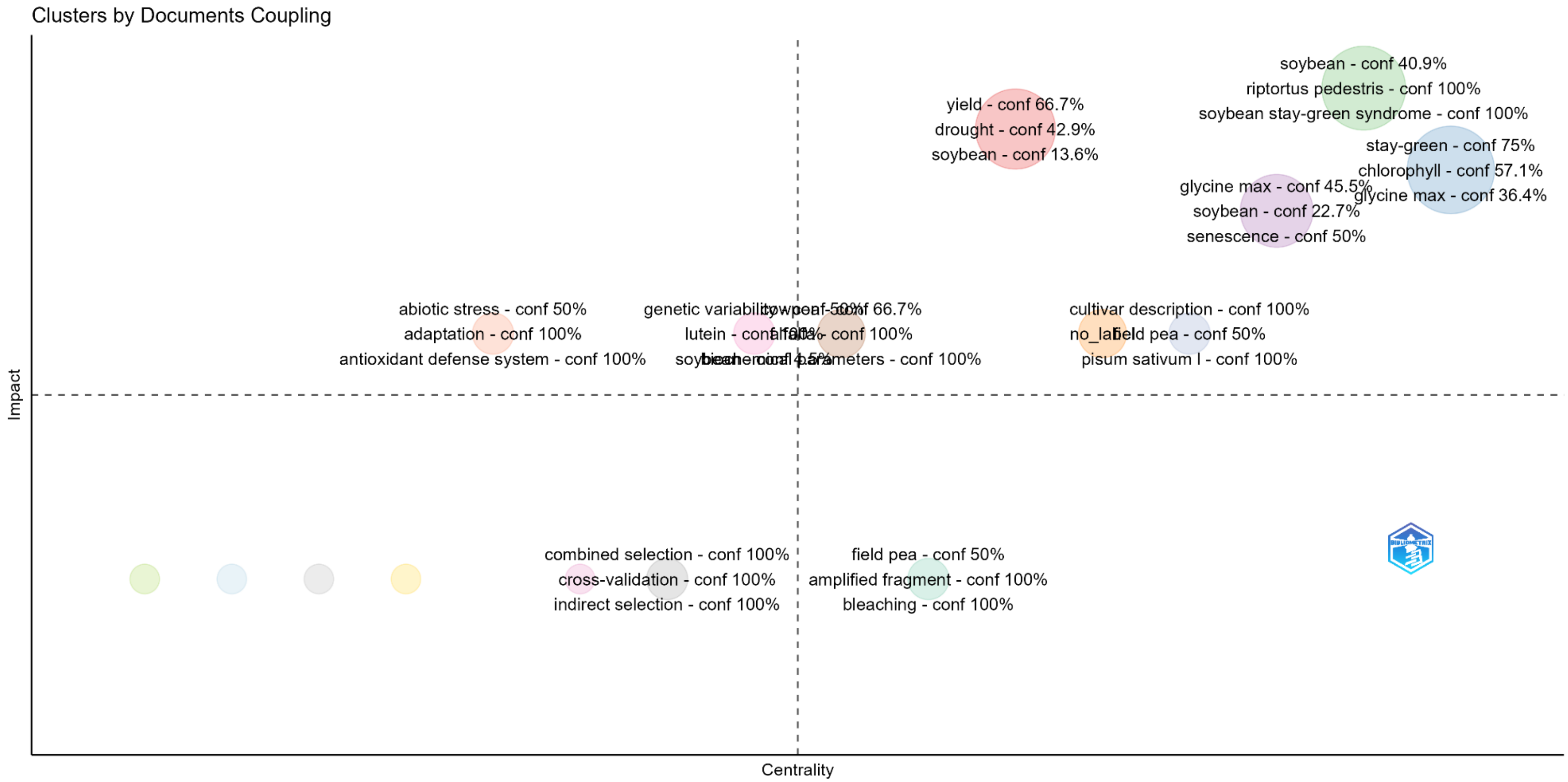

Figure S14: Thematic clusters of stay-green research in legumes based on document coupling analysis.

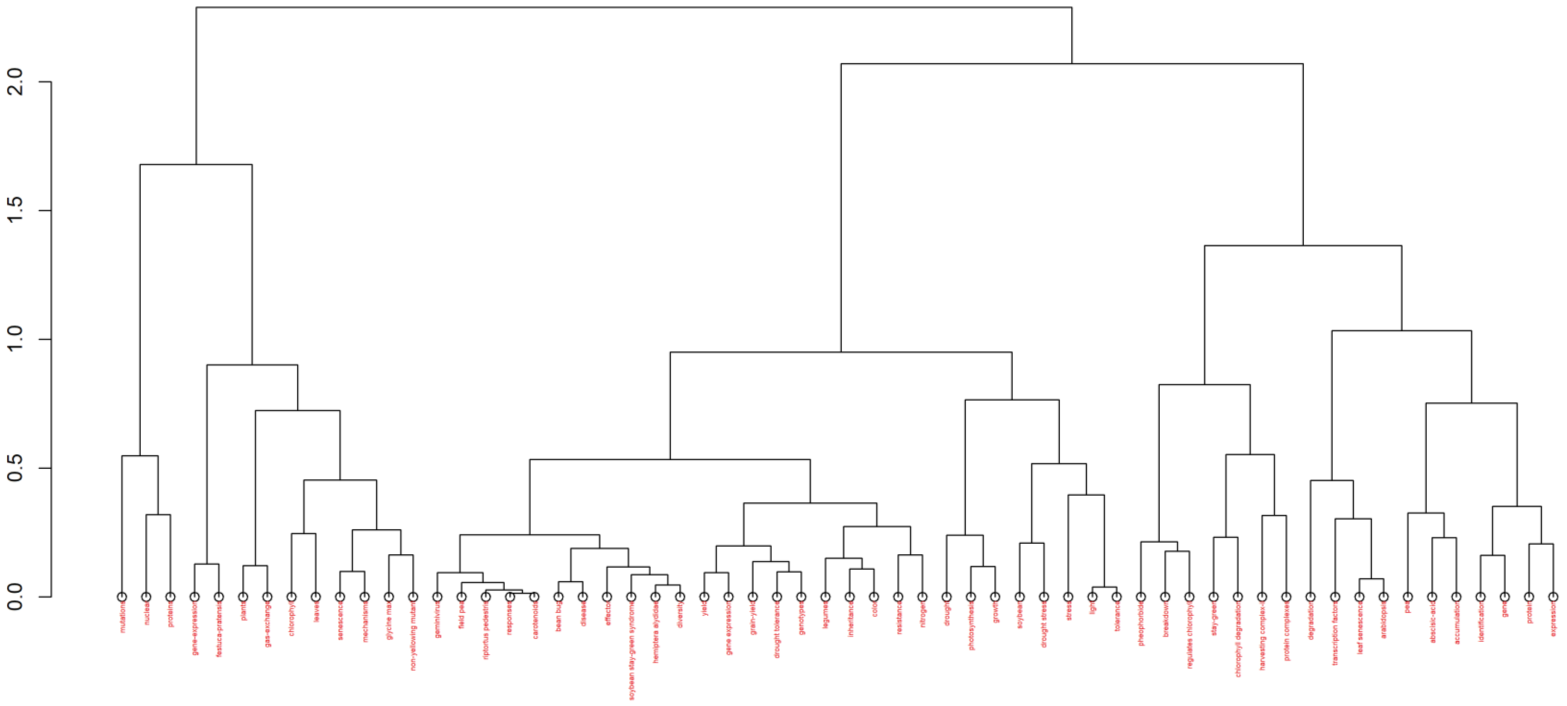

Figure S15: Hierarchical clustering dendrogram of keywords revealing thematic relationships in stay-green research in legumes.

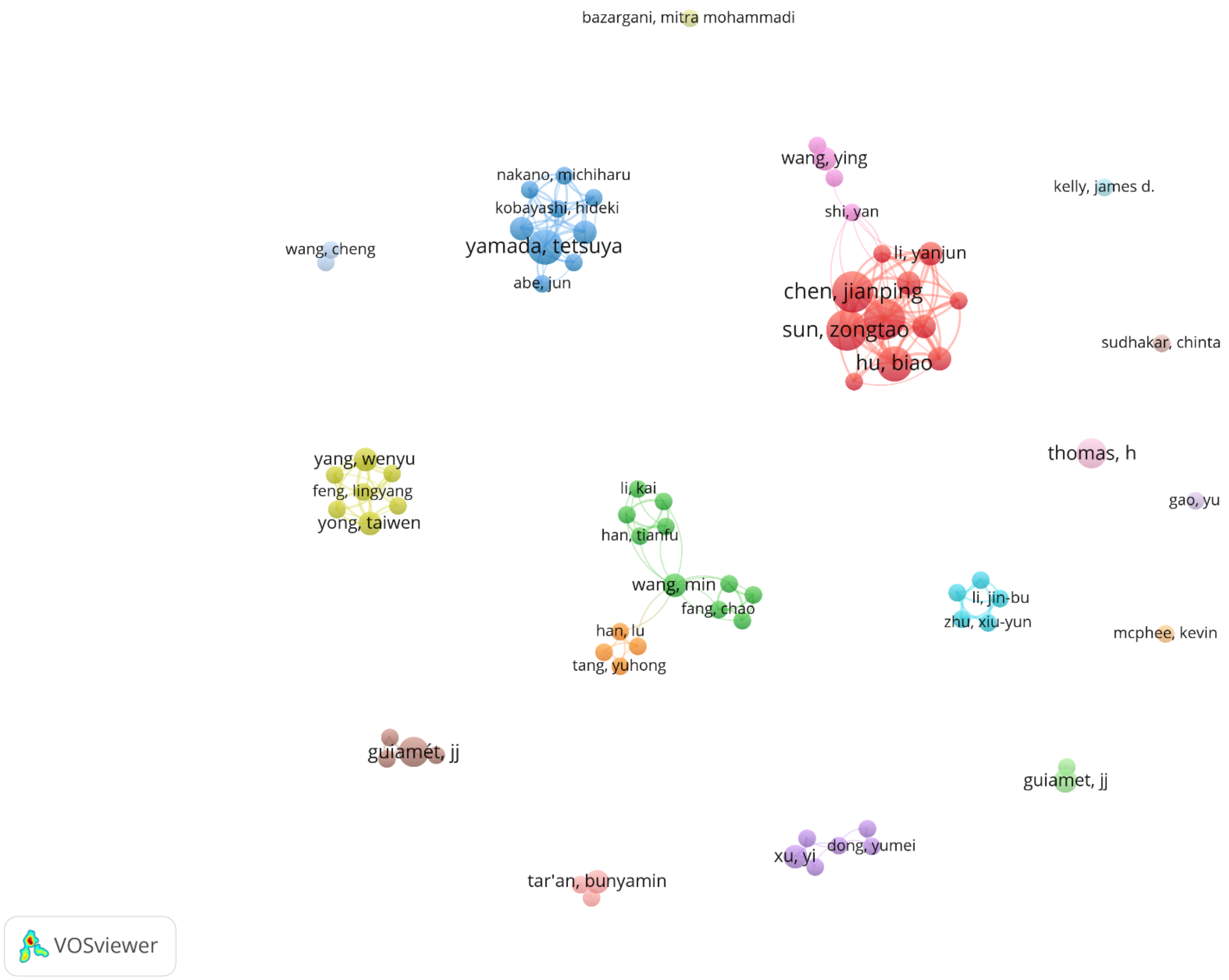

Figure S16: Co-authorship network of authors working on stay green traits in legumes based on VOSviewer analysis.

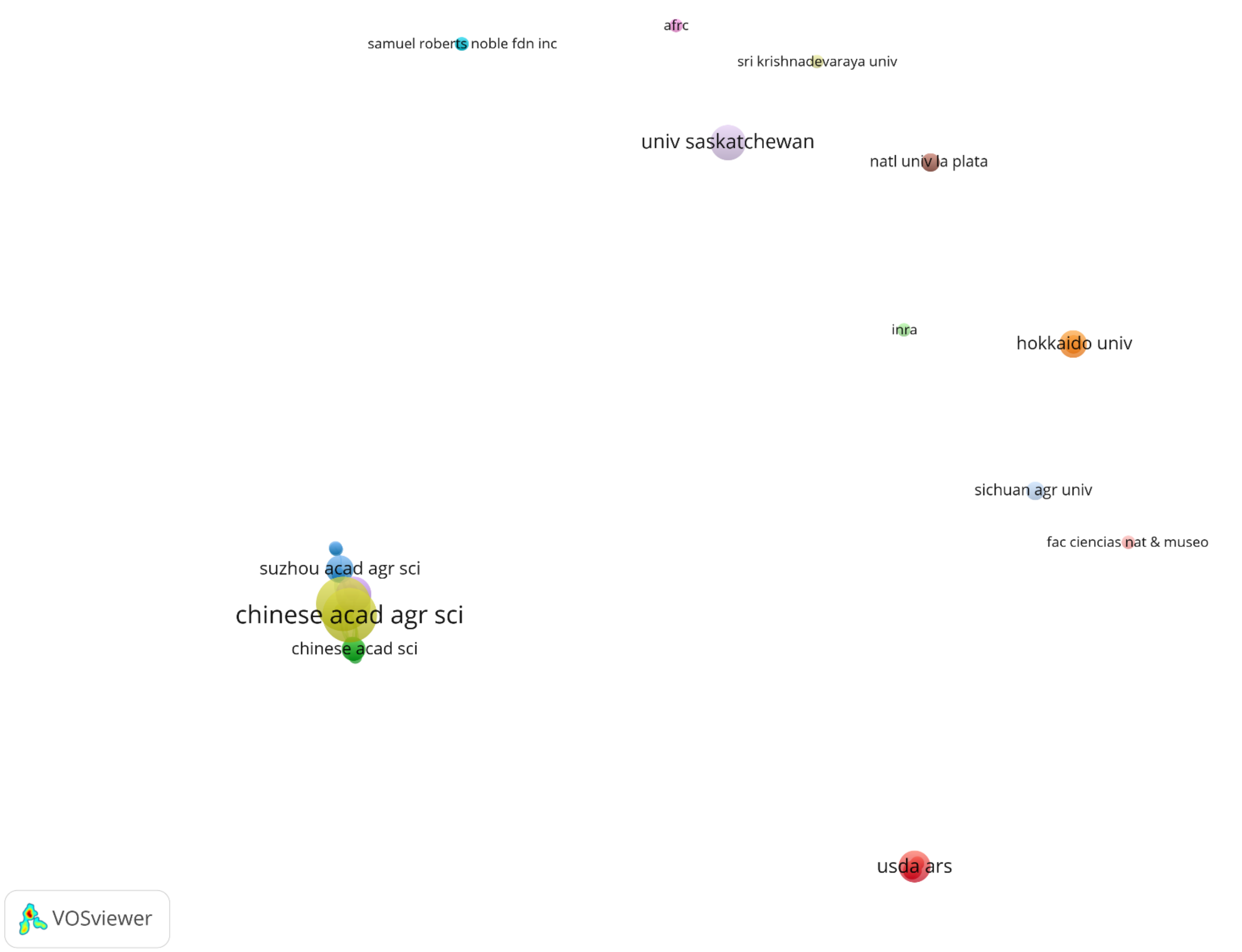

Figure S17: Institutional collaboration network of stay green traits in legumes based on VOSviewer analysis.

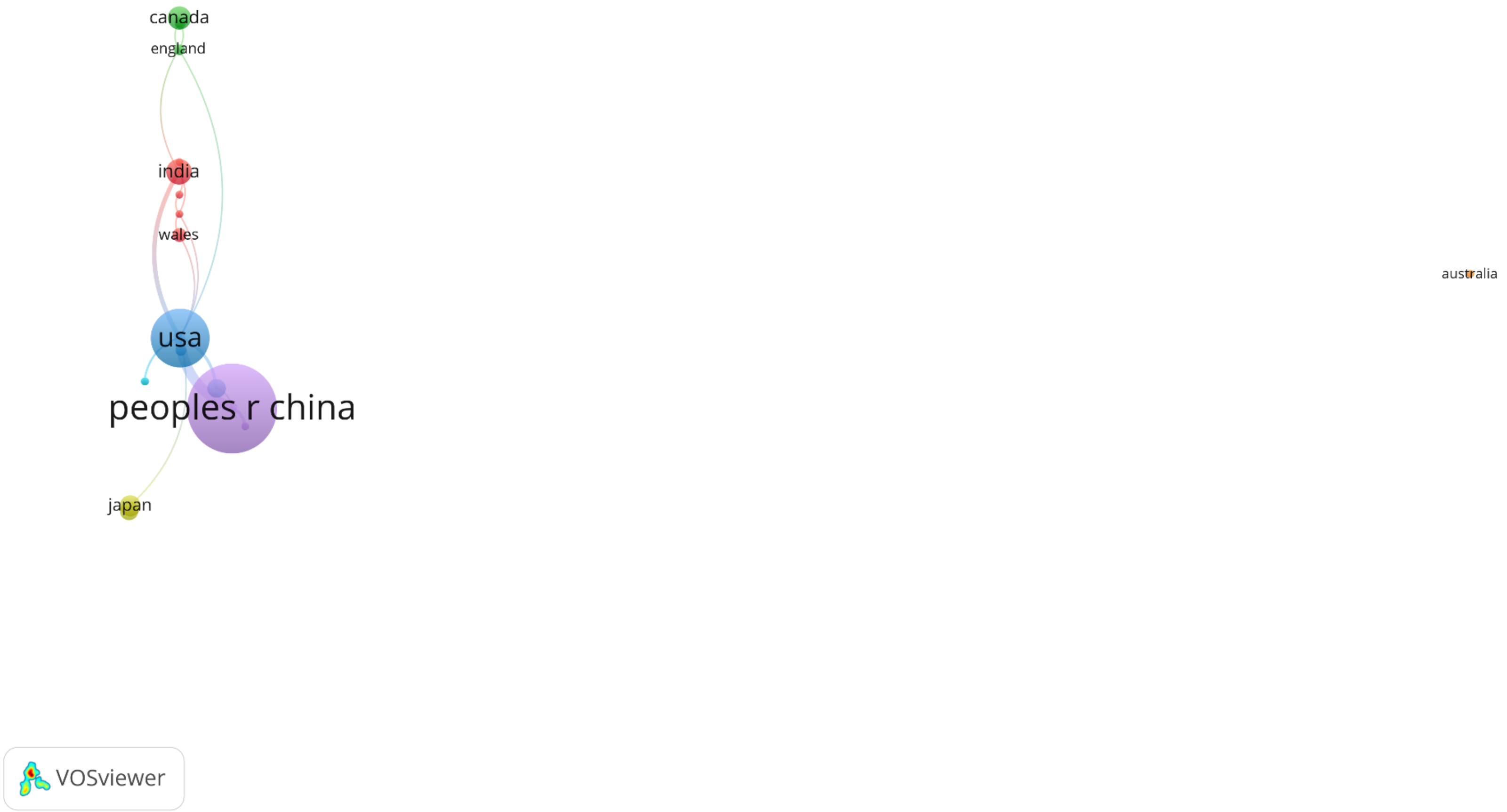

Figure S18: Country level collaboration network in stay green traits in legumes based on VOSviewer analysis.

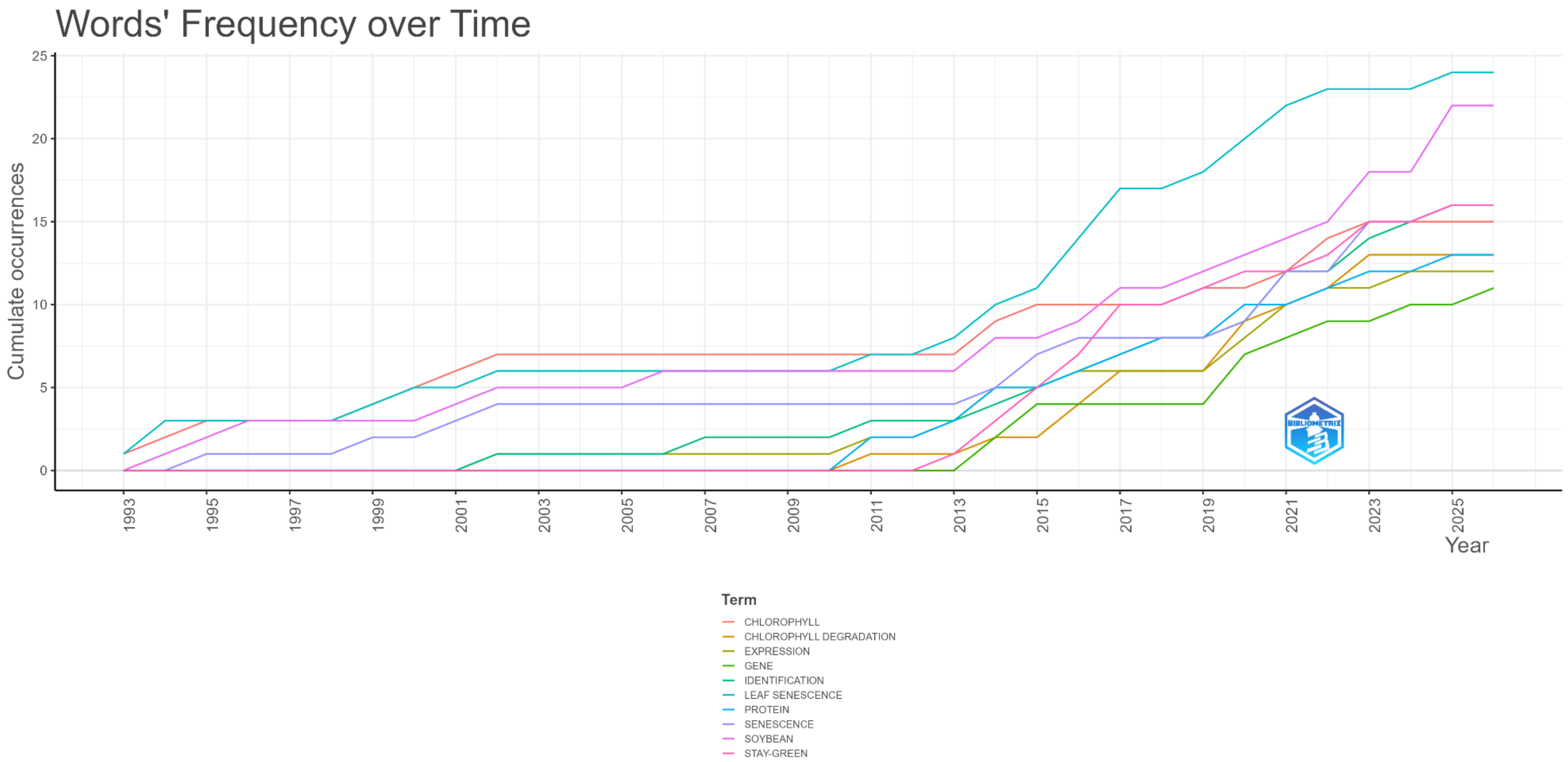

Figure S19: Temporal trends in keyword frequency reflecting the evolution of stay-green research in legumes.

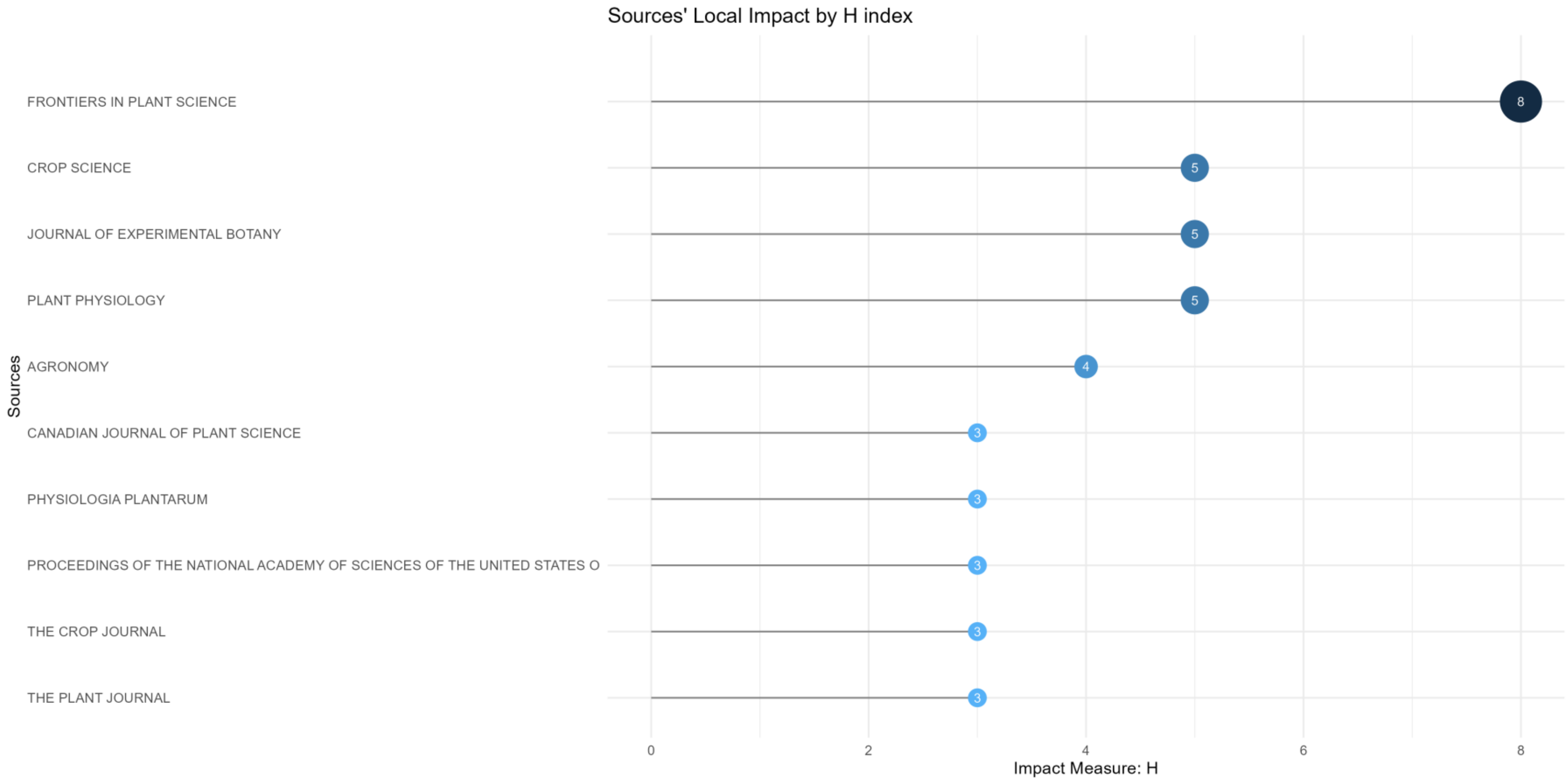

Figure S20: Local impact of journals publishing stay-green research in legumes based on H index.

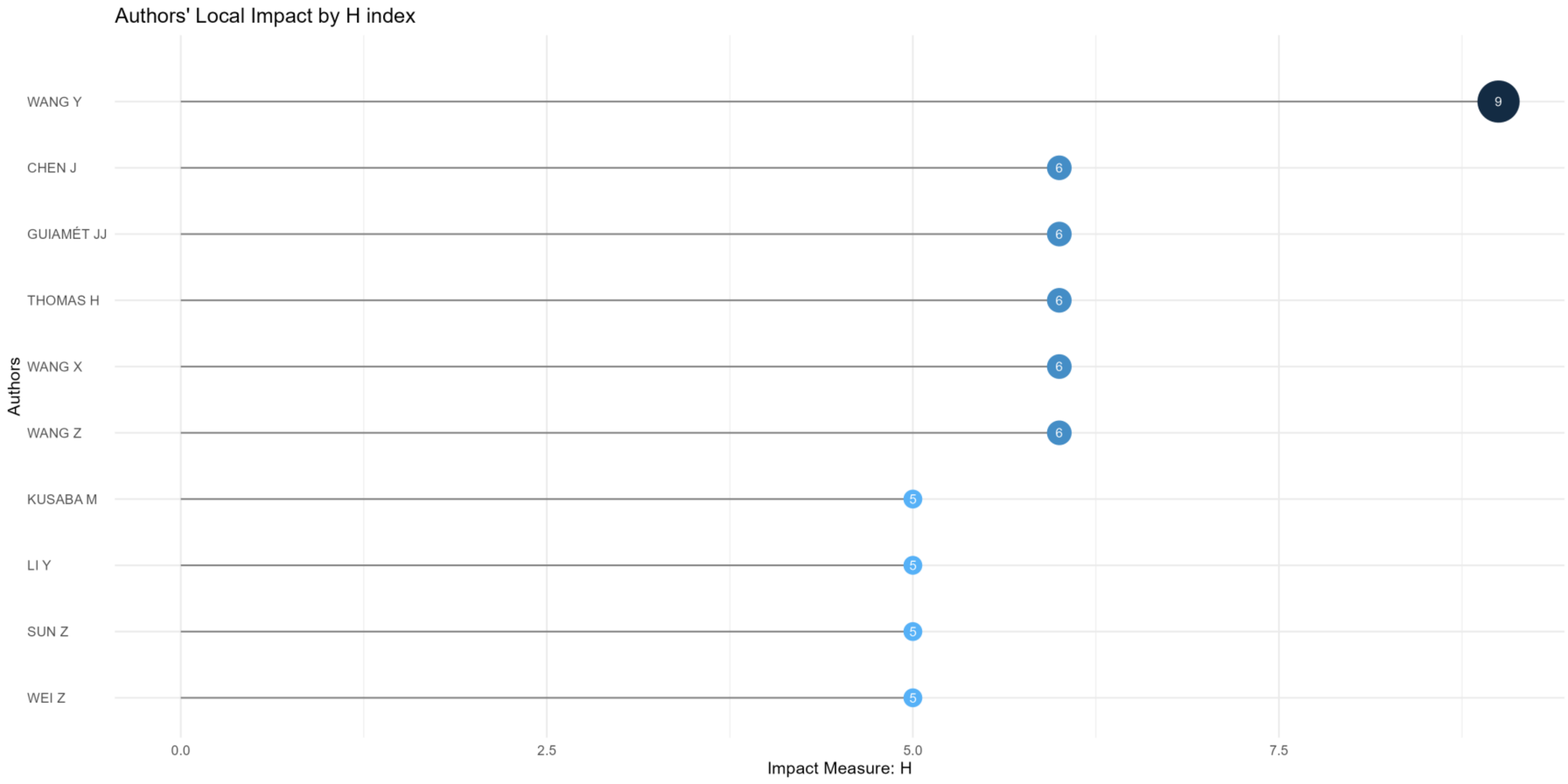

Figure S21: Local impact of authors in stay-green research on legumes based on H-index.

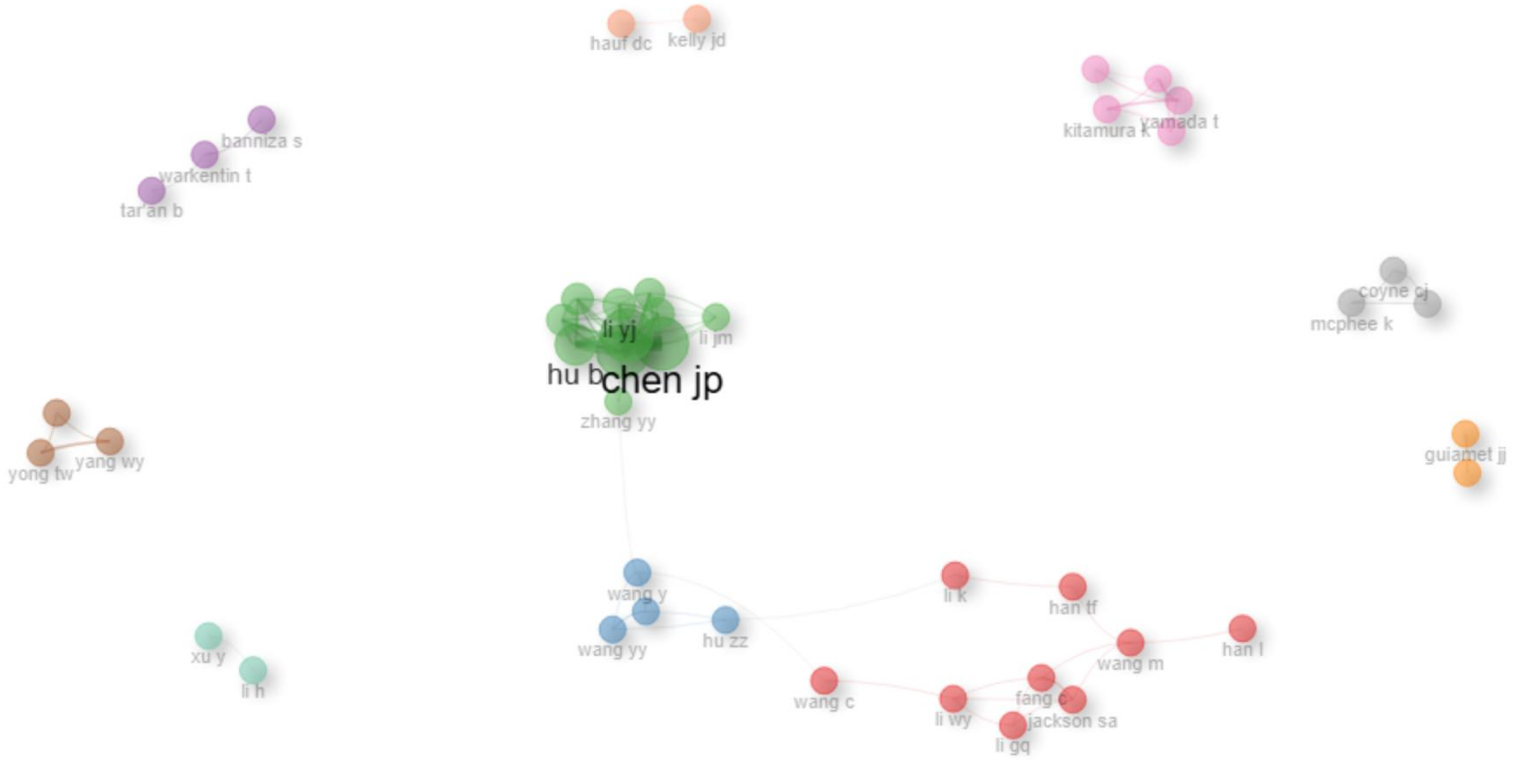

Figure S22: Author collaboration network highlighting major clusters in stay-green studies of legumes

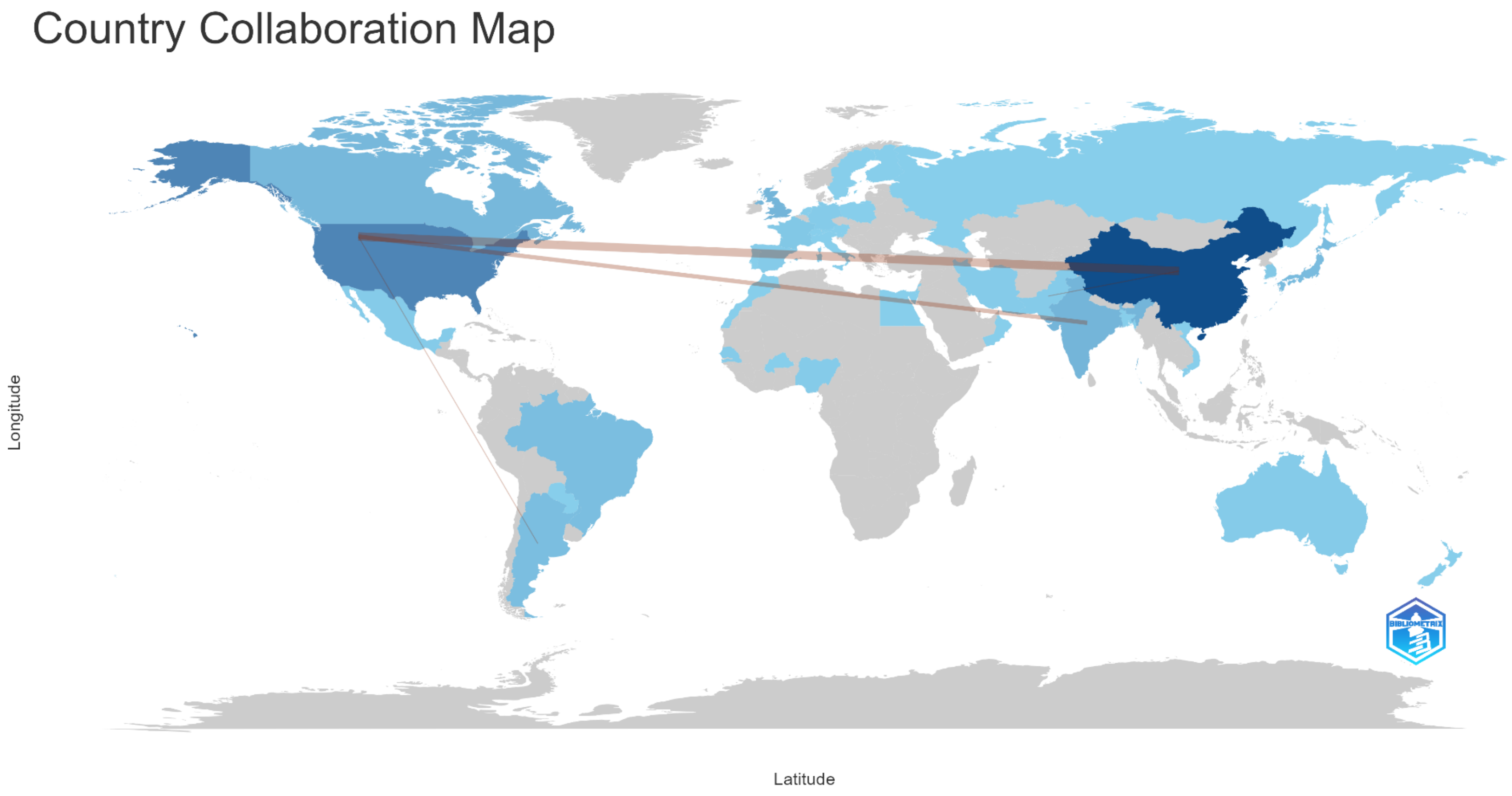

Figure S23: Global country collaboration map illustrating international research linkages in stay-green trait studies of legumes.

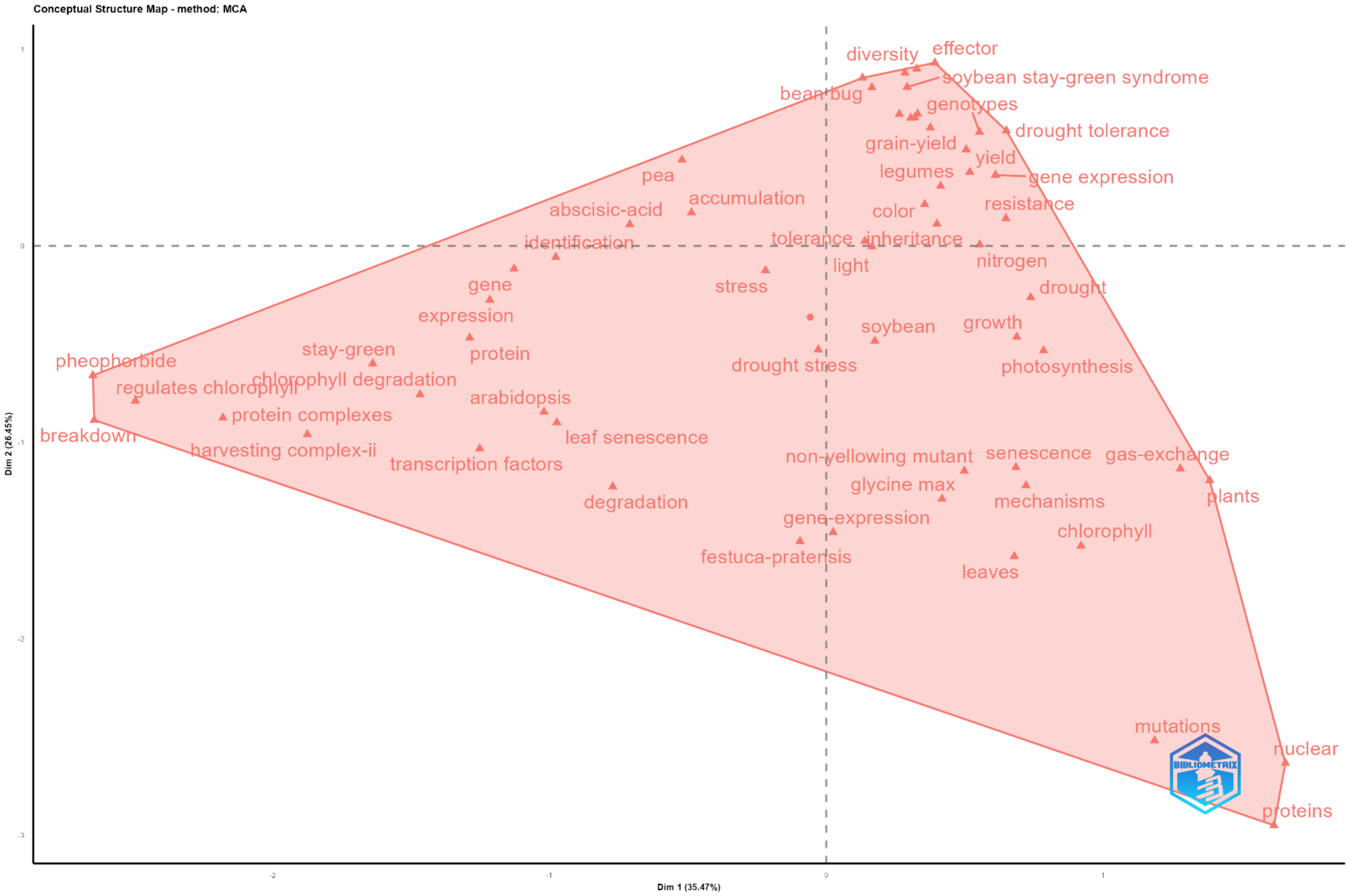

Figure S24: Conceptual structure map of stay-green research in legumes derived using Multiple Correspondence Analysis (MCA).

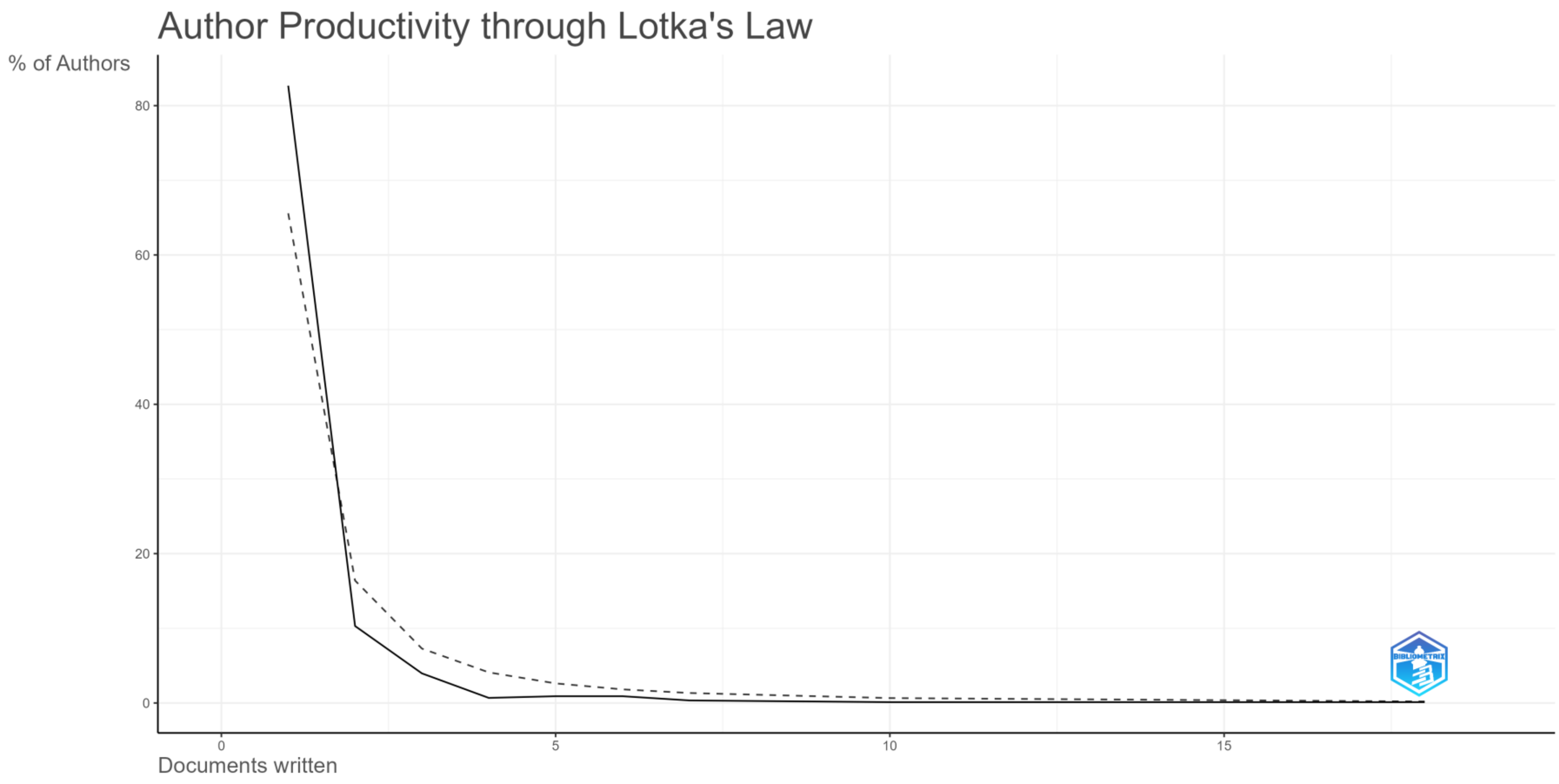

Figure S25: Distribution of author productivity in stay-green research on legumes following Lotka’s Law.
